# Cost comparison of the ShangRing device versus Mogen clamp for early infant male circumcision in Sub-Saharan Africa

**DOI:** 10.1371/journal.pone.0360356

**Published:** 2026-10-13

**Authors:** Nahid Punjani, Tenny R. Zhang, Spyridon P. Basourakos, Mark A. Barone, Alice Christensen, Godfrey Kigozi, Marc Goldstein, Philip S. Li, Alvin I. Mushlin, John Stover, Richard K. Lee

**Affiliations:** 1 Department of Urology, NewYork-Presbyterian/Weill Cornell Medicine, New York, New York, United States of America; 2 Department of Urology, Mayo Clinic Arizona, Phoenix, Arizona, United States of America; 3 Department of Urology, Boston Children’s Hospital, Boston, Massachusetts, United States of America; 4 Department of Urology, Case Western Reserve University School of Medicine, Cleveland, Ohio, United States of America; 5 The Population Council, New York, New York, United States of America; 6 Jhpiego, Dar es Salaam, Tanzania; 7 Rakai Health Sciences Program, Entebbe, Uganda; 8 Department of Population Health Sciences, Weill Cornell Medicine, New York, New York, United States of America; 9 Avenir Health, Glastonbury, Connecticut, United States of America; IAVI, UNITED STATES OF AMERICA

## Abstract

**Introduction:**

The World Health Organization (WHO) endorses male circumcision as an effective human immunodeficiency virus (HIV) prevention intervention in settings where the expected impact is greatest, including Sub-Saharan Africa. Data on cost implications of early infant male circumcision (EIMC) are limited. The ShangRing (SR) is a WHO pre-qualified device for circumcision. We sought to perform a comprehensive cost assessment alongside a randomized controlled trial (RCT) of the SR versus the standard of care Mogen clamp (MoC) for EIMC to enable future scale-up towards a cost-effective HIV prevention strategy.

**Methods:**

We collected resource utilization and economic data as part of an RCT of infants aged 0–60 days old undergoing EIMC in Kenya, Tanzania, and Uganda. Cost data for both the SR and MoC included baseline facility costs, direct costs, and indirect costs. The Decision Makers Program Planning Tool v1.0 was used to perform a direct cost comparison of these two devices. The Goals model was used to compare the costs of EIMC in 2023 using the present value of money to circumcise an adolescent male 15 years from now.

**Results:**

A total of 1,378 infant males underwent circumcision, 689 via SR and 689 via MoC. The overall cost of a single EIMC in Kenya, Tanzania, and Uganda were 61.30 United States Dollars ($), $47.14, and $64.59 respectively for the SR, and $56.38, $50.43, and $58.57 respectively for the MoC. In Kenya, EIMC would be less expensive based on nominal present costs in 2023 compared to waiting for adolescent circumcision at age 15 years in 2038. This finding is reversed in Tanzania and Uganda.

**Conclusions:**

The overall cost of EIMC via SR versus MoC is comparable in an RCT. With scale-up utilizing lower level personnel, the cost of SR may be further reduced. The SR is an attractive alternative for future EIMC initiatives in Sub-Saharan Africa to further reduce HIV burden.

## Introduction

Sexual transmission remains the most common means for human immunodeficiency virus (HIV) spread, and significant evidence exists demonstrating the protective effect of male circumcision against HIV infection [1]. The male foreskin is composed of a non-keratinized mucosal layer lined with Langerhans cells which can host the virus and increase disease transmission [1]. Both observational studies and randomized controlled trials (RCTs) in Africa have demonstrated that voluntary medical male circumcision (VMMC) is associated with reduction in male HIV acquisition by up to 60% [2–6]. Consequently, the World Health Organization (WHO) and the Joint United Nations Program on HIV/AIDS (UNAIDS) recommend VMMC as a prevention strategy for countries with low rates of male circumcision and high rates of heterosexually transmitted HIV [7]. Early infant male circumcision (EIMC) is one form of VMMC being studied due to its procedural simplicity, rapid wound healing, and similar long-term benefits compared to both adolescent and adult male circumcision [8,9].

While there are numerous devices that may be used for EIMC including the Plastibell device, Gomco clamp, Mogen clamp (MoC), and AccuCirc, the ShangRing (SR) is currently the only WHO pre-qualified device which meets its rigorous global standards of quality, safety, and efficacy, while ensuring availability and affordability [10,11]. The SR, developed in 2003, is a disposable device designed to be safe, quick, and easy to learn by healthcare providers, especially as no additional hemostasis is required [12]. The device consists of an internal ring placed directly around the head or glans of the penis (under the foreskin) and an outer ring placed around the foreskin allowing for passive device removal [10]. While the cost effectiveness of MoC has been clearly demonstrated in certain target populations, given the many comparative advantages of SR for male circumcision (e.g., single-use, disposable, short procedure time, better cosmesis, no need for hemostasis, and reasonable learning curve), it is potentially a more cost-effective device for VMMC scale-up towards achieving the WHO’s goal of increasing male circumcision in priority countries as a method of partial lifelong protection and global HIV prevention [13–15].

To date, there have been limited data quantifying the cost implications of EIMC using different devices. We therefore performed an economic evaluation alongside an RCT in three Sub-Saharan African countries (Kenya, Tanzania, and Uganda) at 11 different community and referral hospitals to study the cost of the SR compared to the standard of care MoC. We focus this report on our comparative cost analysis as a follow-up to the initial feasibility study [16].

## Materials and methods

### Trial design

We performed an RCT with healthy neonates aged 0−60 days old with a gestational age ≥ 37 weeks and birth weight ≥2.5 kg from 11 hospitals across Kenya (Homa Bay County Teaching and Referral Hospital, Kisumu Referral Hospital), Tanzania (Ngome Health Center, Iringa Regional Referral Hospital, Mafinga District Hospital, Ilula Hospital), and Uganda (Kakuuto Health Centre IV, Kalisizo General Hospital, Lyantonde General Hospital, Masaka Regional Referral Hospital, Rakai General Hospital) from September 17, 2018 to December 20, 2019 [16]. Participants were randomly assigned 1:1 through a text-message service to undergo circumcision with SR or MoC and were followed until complete wound healing. Infants with perinatal illness requiring treatment, penile abnormalities, allergies to anesthesia, or family history of bleeding diathesis were excluded. The primary trial endpoints were adverse events, safety, and efficacy. The trial was reviewed and approved by the Weill Cornell Medical College Institutional Review Board (IRB) [submission number 1611017762−14] as well as competent IRBs and medical councils from each collaborating organization and participating country. The RCT was also registered on ClinicalTrials.gov (NCT03338699). Written informed consent was obtained from infants’ parents or legally authorized representatives. All consent forms and written material were provided in both English and Swahili.

### Cost data collection and management

Data collection surveys completed by each study site within 1 year of the RCT were derived from the Decision Makers Program Planning Tool (DMPPT) v1.0, a standard tool utilized to assess cost related to male circumcision in Sub-Saharan Africa [14,15]. DMPPT is a Microsoft Excel-based modeling tool that calculates the cost of the intervention based on locally derived information of staff time, salaries, supplies, equipment, and shared facility costs. It was developed by the United States Agency for International Development (USAID) Health Policy Initiative in conjunction with UNAIDS to document and understand the economic consequences of various options for scaling up VMMC [14,15]. The model output provides total procedural cost for male circumcision using each device, facilitating a direct cost comparison. Because the DMPPT also includes projections of relevant health outcomes including HIV cases averted, it has been used to calculate the cost effectiveness of the alternative policy options including the choice of various devices. Further information can be found in the DMPPT manual (see S1 File).

Data were collected from all 11 community and referral hospitals in Kenya, Tanzania, and Uganda using forms based on the required DMPPT inputs. The data were separated into four forms and included: (i) baseline institutional circumcision cost assessments, (ii) direct procedural costs using the SR and (iii) MoC devices, and (iv) indirect costs needed to complete these procedures at the respective hospitals/institutions. The forms were distributed to each country through the study site coordinators and/or representatives. While baseline forms were completed at the beginning of the trial, the remaining forms were completed afterwards based on their actual experience performing circumcision during the trial. Of note, sites were aware of the intent to collect cost information and instructed to prospectively record these cost data throughout the trial. The forms were reviewed and data analyzed based on input from each of the trial sites. There were no reading level requirements or restrictions as to who was permitted to complete the forms, but all were filled out electronically and completed in English by the local trial study staff. All data were transferred to the main study location in the United States for analysis by team members not involved in data collection.

The baseline form recorded facility data including number of outpatient visits and inpatient days over the past year related to circumcision, as well as the number of circumcisions performed at the site in the year prior to the trial. Information about male circumcision complications prior to initiation of the trial of infant circumcision was also collected. The direct costs form for both SR and MoC circumcisions during the trial collected data on the number of procedures completed and device costs. Personnel costs (including salary, benefits, and number of hours performing circumcision), consumable supplies, and drug-related costs and quantities used were recorded before, during, and after circumcision. Information regarding costs of training were also tabulated. Despite many personnel (including clinical officers and nurses) having previous knowledge of the standard MoC procedure, everyone was trained to complete both the SR and MoC procedures. The indirect cost forms focused on costs related to the facility including the item purchase costs and amortization, utility costs, as well as costs related to maintenance. Facility support personnel were also listed including their salary and time/amount of effort devoted to circumcision. These indirect costs were assumed to apply equally to both devices. The data from these four forms were compiled into an analytic database to allow for both inter-site and inter-country comparison.

### Cost analysis and assumptions

Cost performance analysis was performed in two separate stages. All cost calculations were performed from the health system perspective. The first utilized the DMPPT model. Total per-procedure item cost was based on the frequency of use, the number of items used, and the actual purchase cost of each item required. For non-consumable items when the number of uses before being discarded or used-up were unavailable, the total purchase cost was divided by the number of procedures performed during the trial. If any site used a centralized supply chain, the governmental records and ministries of health were queried for costs. For SR, costs were based on the number of devices used and wasted for the trial, in addition to the number of devices used for training. The personnel costs for each procedure were derived from the time required by each type of personnel for each procedure in minutes either performing or assisting, divided by the total number of procedures performed. When the amortization period for indirect costs were not available, a standard assumption of 7 years was applied.

The second analysis compared the costs of EIMC in 2023 (year of analysis) using the present value of money to circumcise an adolescent male 15 years from now (e.g., in 2038) in order to gain a more forward-thinking view on cost in the context of longer-term goals for HIV prevention. A structured Excel-based Goals simulation model (see S2 File) was used to estimate the effects of interventions and resource allocation on HIV outcomes using Spectrum software (Avenir Health) [17]. These calculations were performed for each country factoring in the total cost of EIMC with SR in each location, the estimated proportion of the population surviving to age 15 years, a standard 3% discount rate, and the “preferred cost” of adolescent VMMC determined by expert consensus. In doing so, we arrived at a projected EIMC cost per person surviving to age 15 years for each country, as well as a “present value” of the amount of money needed to compound at 3% interest and arrive at the preferred cost of VMMC 15 years from now.

### Statistical analysis

Cost analysis was done using the DMPPT model, which is an ingredients-based model that accounts for the cost of commodities, supplies, and health personnel time (see S1 File). Cost projections were performed using the Goals model, a spreadsheet-based mathematical simulation tool implemented in Microsoft Excel (see S2 File). The Goals model estimates the number of HIV infections averted by circumcising infants versus 15-year-olds by taking into account mortality by age and the future impact of male circumcision on new infections throughout the lifetime of those circumcised in a 15 year period. Finally, sensitivity analyses for SR were performed by varying the unit device cost (5, 10, and 15 United States Dollars [$]) and by assessing alternative costing scenarios: used devices only, used devices plus wastage, and used devices plus wastage and training costs. For MoC, sensitivity analyses were conducted by varying the amortization of the device over the number of cases performed during the trial, as well as over assumed lifetimes of 500 and 1,000 uses.

## Results

Overall, 1,378 infants underwent EIMC with either SR (n = 689) or MoC (n = 689) at the 11 trial sites. A total of 229 (33.2%) SR circumcisions were completed in Kenya, 230 (33.4%) in Tanzania, and 230 (33.4%) in Uganda. Similarly, 230 (33.4%) MoC circumcisions were completed in Kenya, 229 (33.2%) in Tanzania, and 230 (33.4%) in Uganda. As previously reported, the trial demonstrated that the SR is safe, with comparable postoperative pain scores, cosmesis, and high parent/legal authorized representative satisfaction [16]. All participants demonstrated complete wound healing by postoperative day 42, with similar rates of adverse events in both arms (<10%). Mean duration of procedures was similar overall with 12.3 ± 8.0 minutes for SR EIMC and 12.6 ± 9.1 minutes for the MoC (p = 0.577).

EIMC costs using either the MoC or SR in each country and subsequent sites are shown in Table 1. Using the SR, the average cost in Kenya was $61.30, in Tanzania $47.14, and in Uganda $64.59. For the MoC, the average cost in Kenya was $56.38, in Tanzania $50.43, and finally in Uganda $58.57. In the majority of sites, these costs were mainly driven by direct costs.

**Table 1:** Summary of direct and indirect costs (USD).

	Direct Cost		Indirect Cost		TOTAL (country averages)	Direct Cost		Indirect Cost		TOTAL (country averages)
	Cost	% of Total	Cost	% of Total		Cost	% of Total	Cost	% of Total	
ShangRing						Mogen Clamp				
**Kenya**					**(61.30)**					**(56.38)**
Kisumu	49.38	100	0.05	0	49.44	46.51	100	0.05	0	46.57
Homa Bay	73.03	100	0.14	0	73.16	66.05	100	0.14	0	66.19
**Tanzania**					**(47.14)**					**(50.43)**
Iringa	40.24	98.5	0.62	1.5	40.85	44.90	98.7	0.62	1.3	45.51
Ilula	39.55	97.9	0.85	2.1	40.40	47.18	98.2	0.85	1.8	48.03
Mafinga	40.14	92.7	3.17	7.3	43.31	39.63	92.5	3.17	6.5	42.80
Ngome	49.53	77.4	14.45	22.6	63.98	50.93	77.9	14.45	22.1	65.38
**Uganda**					**(64.59)**					**(58.57)**
Rakai	64.49	100	0.14	0	64.64	58.48	100	0.14	0	58.62
Kalisizo	64.49	100	0.03	0	64.52	58.48	100	0.03	0	58.51
Kakuuto	64.49	100	0.23	0	64.72	58.48	100	0.23	0	58.71
Lyantonde	64.49	100	0.06	0	64.55	58.48	100	0.06	0	58.54
Masaka	64.49	100	0.01	0	64.50	58.48	100	0.01	0	58.49

The proportion of cases completed by the type of lead operator is shown in Table 2. For all three countries, the lead operators were similar between sites with the vast majority of cases performed by nurses. However, a higher proportion of cases were completed by clinical officers in Uganda.

**Table 2:** Proportion of cases completed by the type of lead operator at each site.

	ShangRing		Mogen Clamp	
	Lead Operator Type	Proportion of Cases Completed	Lead Operator Type	Proportion of Cases Completed
**Kenya**				
Kisumu	Nurse	100%	Nurse	100%
Homa Bay	Nurse	66.9%	Nurse	45.0%
	Clinical Officer	33.1%	Clinical Officer	55.0%
**Tanzania**				
Iringa	Registered Nurse	81.8%	Registered Nurse	96.6%
	Enrolled Nurse	18.2%	Enrolled Nurse	3.4%
Ilula	Registered Nurse	81.2%	Registered Nurse	75.0%
	Enrolled Nurse	18.8%	Enrolled Nurse	25.0%
Mafinga	Registered Nurse	41.2%	Registered Nurse	46.1%
	Enrolled Nurse	58.8%	Enrolled Nurse	53.9%
Ngome	Registered Nurse	73.9%	Registered Nurse	67.3%
	Enrolled Nurse	15.2%	Enrolled Nurse	21.8%
	Clinical Officer	10.9%	Clinical Officer	10.9%
**Uganda**				
Rakai	Nurse	62.6%	Nurse	70.0%
Kalisizo	Clinical Officer	37.4%	Clinical Officer	30.0%
Kakuuto				
Lyantonde				
Masaka				

Sensitivity analyses based on the costs of the SR device are shown in Table 3. The costs attributable to devices either wasted or used in training were minimal (as low as $0.71 difference in Tanzania or as high as $3.20 in Uganda per case). Cost variability was greater when the device cost was increased incrementally by $5. We performed sensitivity analyses for MoC costs when number of circumcisions is scaled up. As shown in Table 4, when comparing the cost of using a single device to perform the number of cases in the actual trial at each site versus the ability to use a MoC device in either 500 or 1,000 cases, the cost drops at 500 uses, but when scaled up to 1,000 uses, further cost and variation reductions are minimal.

**Table 3:** Sensitivity analyses for ShangRing (total cost in USD).

	Used	Used + Wasted	Used + Wasted + Training	$5	$10	$15
**Kenya**						
Kisumu	49.44	49.52	51.75	42.68	47.64	52.60
Homa Bay	73.16	74.65	75.80	68.63	73.59	78.55
**Tanzania**						
Iringa	40.85	41.23	41.43	35.29	40.29	45.29
Ilula	40.40	41.23	41.65	34.84	39.84	44.84
Mafinga	43.31	43.59	43.88	37.75	42.75	47.75
Ngome	63.98	64.21	64.44	58.42	63.42	68.42
**Uganda**						
Rakai	64.64	64.54	67.70	59.44	63.73	68.02
Kalisizo	64.52	64.53	67.69	61.47	65.76	70.04
Kakuuto	64.72	64.73	67.89	61.67	65.96	70.24
Lyantonde	64.55	64.56	67.72	61.50	65.79	70.07
Masaka	64.50	64.51	67.67	61.45	65.74	70.02

**Table 4:** Sensitivity analyses for Mogen Clamp (total cost in USD).

	Y Uses	500 Uses	1000 Uses
**Kenya**			
Kisumu	46.57	37.38	36.51
Homa Bay	66.19	44.21	43.90
**Tanzania**			
Iringa	45.51	37.74	37.22
Ilula	48.03	36.07	35.55
Mafinga	42.80	37.00	36.48
Ngome	65.38	56.97	56.45
**Uganda**			
Rakai	58.62	58.15	58.12
Kalisizo	58.51	58.04	58.01
Kakuuto	58.71	58.24	58.21
Lyantonde	58.54	58.07	58.04
Masaka	58.49	58.02	57.99

In terms of the estimated costs of present-day EIMC versus adolescent VMMC in 15 years, the results varied by country and are summarized in Fig 1 and Table 5. In Kenya, EIMC would be less expensive based on nominal present costs in 2023 compared to waiting for adolescent circumcision at age 15 years in 2038. In this example, the calculated EIMC cost per person surviving to age 15 years in Kenya is $74.73, compared to the projected cost of $100.34 in today’s money to produce the circumcised male in 2038 through adolescent circumcision. In Tanzania and Uganda, this result comparison is reversed, with the projected cost to produce a circumcised male in 2038 being lower than the calculated cost of EIMC per person surviving to 15 years old. However, one should note that the range of possible low to high cost estimates for adolescent VMMC varied widely in each country compared to the nominal cost, such that EIMC could be more cost efficient in Tanzania and Uganda depending on the impact of inflation and interest rates on expected cost of adolescent circumcision.

**Table 5:** Estimated cost of EIMC versus estimated present value of adolescent VMMC in 15 years (USD).

	EIMC	Estimated Present Value of Adolescent VMMC in 15 Years (nominal)	Estimated Present Value of Adolescent VMMC in 15 Years (low)	Estimated Present Value of Adolescent VMMC in 15 Years (high)
**Kenya**	74.73	100.34	21.34	100.34
**Tanzania**	68.18	33.92	31.98	82.54
**Uganda**	50.97	33.38	12.97	54.19

**Fig 1:**
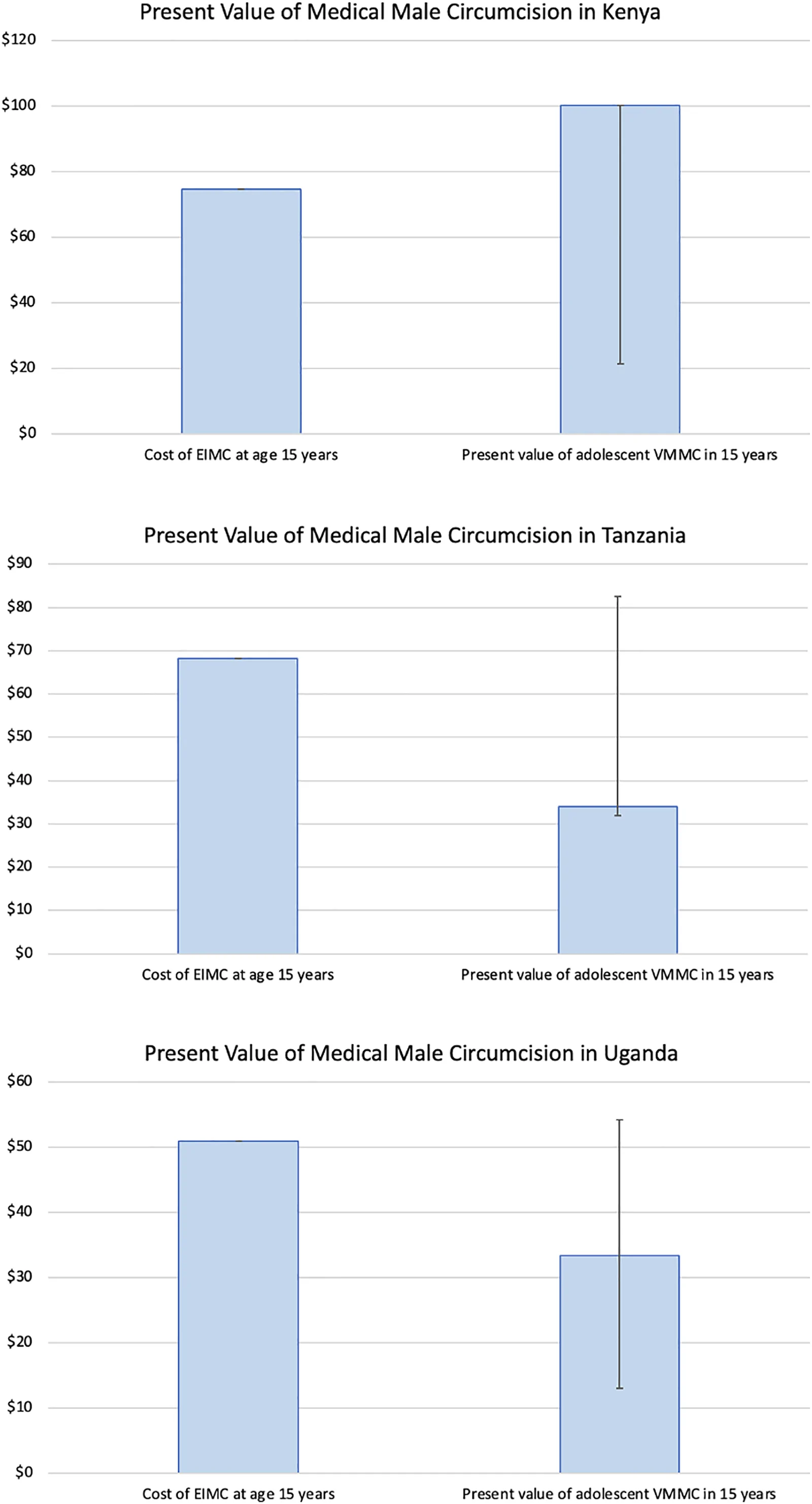
Present value of medical male circumcision in Kenya, Tanzania, and Uganda (USD).

## Discussion

The SR device was developed as a single-use, disposable, and easy to learn device for male circumcision. In the first multi-country RCT comparing it to the MoC, we demonstrate that the cost burden is comparable between devices. The majority of costs are driven by direct costs of supplies, as well as the cost of personnel for the procedure. The projected present-day monetary value of EIMC versus adolescent VMMC in 15 years varied by site.

The overall average costs of EIMC in our study was between $45–65 depending on the site and device used. This is comparable to the literature which reports a wide range of costs for circumcision, depending on the device and region/country where it was performed. A South African study suggested costs of over $100 for circumcision but used a now-discontinued disposable device for male circumcision, PrePex, which itself costs around $60 [18,19]. Alternatively, costs as low as $20 have been reported in countries such as Zimbabwe and Mozambique [20].

While the overall costs for the SR and MoC EIMC were fairly similar in the majority of sites, MoC was slightly less costly in Kenya and Uganda. One possible explanation is related to personnel costs. As our cost analysis was completed as part of an RCT focused primarily on the safety and efficacy of the SR as a new device, many factors were importantly standardized including the personnel performing the procedure. For example, some of the cost differences we observed were driven by differing personnel costs (hourly wages and benefits), as well as by practice variations as seen in Uganda where a higher proportion of the cases were performed by a clinical officer compared to the other countries [21]. Similarly, personnel were generally more familiar and proficient with MoC for EIMC. While not captured in our data, additional costs associated with longer procedural times due to learning curve effect for SR EIMC in the earlier part of the study may also have increased personnel costs. Finally, of note, the study sites used in Kenya and Uganda reported 0% indirect costs compared to Tanzania, implying different VMMC service delivery models which could affect its overall cost performance.

One reason for the increased cost of the SR EIMC in Kenya and Uganda could be traced the cost of the device itself. Scale-up and large volume production of the device could therefore be a key strategy for widespread adoption and use of the SR, as was seen in a study comparing PrePex to conventional surgery in Africa [20]. For example, sensitivity analysis did show improvement in SR cost performance as case count exceeded 500. SR unit cost would likely also be reduced by higher purchase volume discounts if implemented on a widescale basis. Similarly, the costs for reusable devices such as the MoC may also be variable as in some cases the device is used for a large volume of circumcisions and in other circumstances the device may break, wear out, or malfunction after fewer uses. This scenario is especially relevant when an extra MoC device may need to be on hand to avoid unnecessary delays or complications if a device were to break [22]. Additionally, as seen in other surgical literature, the implementation of single-use devices may be economically advantageous, especially considering the time delays or additional indirect costs such as autoclave sterilization to obtain cleaned reusable devices, which were not included in our present analysis [23].

Cost differences were also noted among sites within a country secondary to the amount of facility share currently devoted to circumcision and the patient volume of individual hospitals. Higher volume institutions had smaller indirect costs, which helps reduce MoC costs. On the other hand, smaller centers such as Ngome in Tanzania had a much higher indirect cost compared to almost every other site in the study. This finding is similar to data exploring VMMC at a fixed center versus those done in a mobile clinic which may have less ability to minimize its indirect costs [24]. These results suggest that high-volume EIMC may favor SR. Of note, our experience with pediatric SR circumcision in China suggests that over 200 circumcisions may be performed in 4 hours.

Finally, we performed cost comparison calculations for EIMC versus an alternative of delaying circumcision until adolescent VMMC in 15 years given evidence of declining HIV incidence in Sub-Saharan Africa over the last decade [25], which implies the cost effectiveness of EIMC may change over time. To make more plain the interpretation of present value, take Kenya as an example—if we provide EIMC now, our estimated cost is $61.30 per circumcision. But only 82% will survive to age 15 years, so the cost per person surviving to 15 years is $61.30/0.82 = $74.73. In contrast, one would need $100.34 in a bank now and let it compound at 3% interest annually so that in 2038 we will have $156.33 required to pay for one adolescent circumcision. In this example it costs $100.34 in today’s money to produce the circumcised male in 2038 through adolescent circumcision compared to $74.73 though EIMC. In other words, EIMC is less expensive and thereby has a superior “present value.”

If we take into account the uncertainty around the cost of adolescent circumcision, however, then we can see that the decision becomes more nuanced. For Uganda and Tanzania, adolescent circumcision would be less expensive but there is some probability (given the uncertainties) that EIMC could be less expensive. This caveat is critical as our cost comparison calculations use expert consensus estimates of the nominal cost of adolescent VMMC. In Fig 1 and Table 5, we show the low-end and high-end estimated costs and their associated projected present value, as well as that of the consensus “nominal” adolescent male circumcision cost. Indeed, EIMC may be more cost efficient than adolescent VMMC depending on one’s expectations for the cost of adolescent circumcision in 15 years. Put another way, these nominal VMMC costs are likely to be further modified by significant changes in inflation as well as prevailing interest rates, both factors of which have been significant and not commensurate with recent historical trends in the past few years. Furthermore, from a cost perspective, there is variation driven by multiple factors including secondary and ancillary costs that may not be captured, including heterogeneity in personnel costs, demand, easy of entry, and vertically versus horizontally integrated care.

Our study has several limitations. While this cost analysis was conducted as part of an RCT, and the sites were asked to acquire information in a prospective manner, some of the data were obtained retrospectively. We were limited by the inherent heterogeneity between sites and countries which have variable facilities, personnel, and resources that may lead to cost variability. However, this may mean that our findings are more generalizable to real-world implementation. We were not able to be capture some costs such as the cost of equipment storage, sterilization of reusable items, communication with parents (phone call or text messaging costs), or for the personnel needed for such activities. Costs associated with adverse events such as return clinic visits were not captured by the forms. Also, we were not able to discern the actual amortization periods for products and devices. Finally, by design of the RCT itself, the same providers were performing both the MoC and SR procedures which did not allow us to analyze the effects of ease of use of the SR device by less trained and expensive personnel, e.g., the learning curve effect.

We note that the implications of this study may serve to influence downstream health policy decisions. Cost-effectiveness studies seek to assess the efficiency of a unit dollar to garner a given benefit, in this case an averted HIV infection. However, it is important to remember that this does not capture or account for the effect of externalities. For instance, it may be easier and therefore cheaper to attract expectant mothers for EIMC during prenatal care than it would be to attract adolescents for VMMC. This would improve the cost effectiveness of EIMC and correspondingly degrade the same for adolescent VMMC. In addition, the use of a novel device such as SR that excluded the need for sutures, provides excellent cosmesis, and avoids areas of concern such as uncommon but extremely challenging urethral fistulae reported with other male circumcision techniques may attract a greater portion of the population that would be otherwise be “VMMC-hesitant.”

## Conclusions

The SR is a safe and low-cost alternative device for EIMC. With scale-up and task shifting to personnel requiring less training, the costs of the SR may be further reduced. EIMC using disposable devices such as SR should continue to be part of the armamentarium for HIV prevention initiatives in Sub-Saharan Africa. In an era of declining HIV incidence in this region of the world, it is challenging but critical to understand device-related costs and to compare the cost effectiveness of EIMC to adolescent VMMC in the future.

## Supporting information

S1 File DMPPT manual. (PDF)

S2 File Goals manual. (PDF)

S3 File Study dataset. (XLSX)
