# Supplementary material for: Cost comparison of the ShangRing device versus Mogen clamp for early infant male circumcision in Sub-Saharan Africa
Source: PLoS One. 2026 Oct 13;21(10):e0360356. doi: 10.1371/journal.pone.0360356 (PMC13660899; doi:10.1371/journal.pone.0360356)
Supplement: Supplementary file 1 [file pone.0360356.s001.pdf]

Decision Makers' Program Planning Tool (DMPPT)

Calculating the Costs and Impacts of a Male Circumcision Program

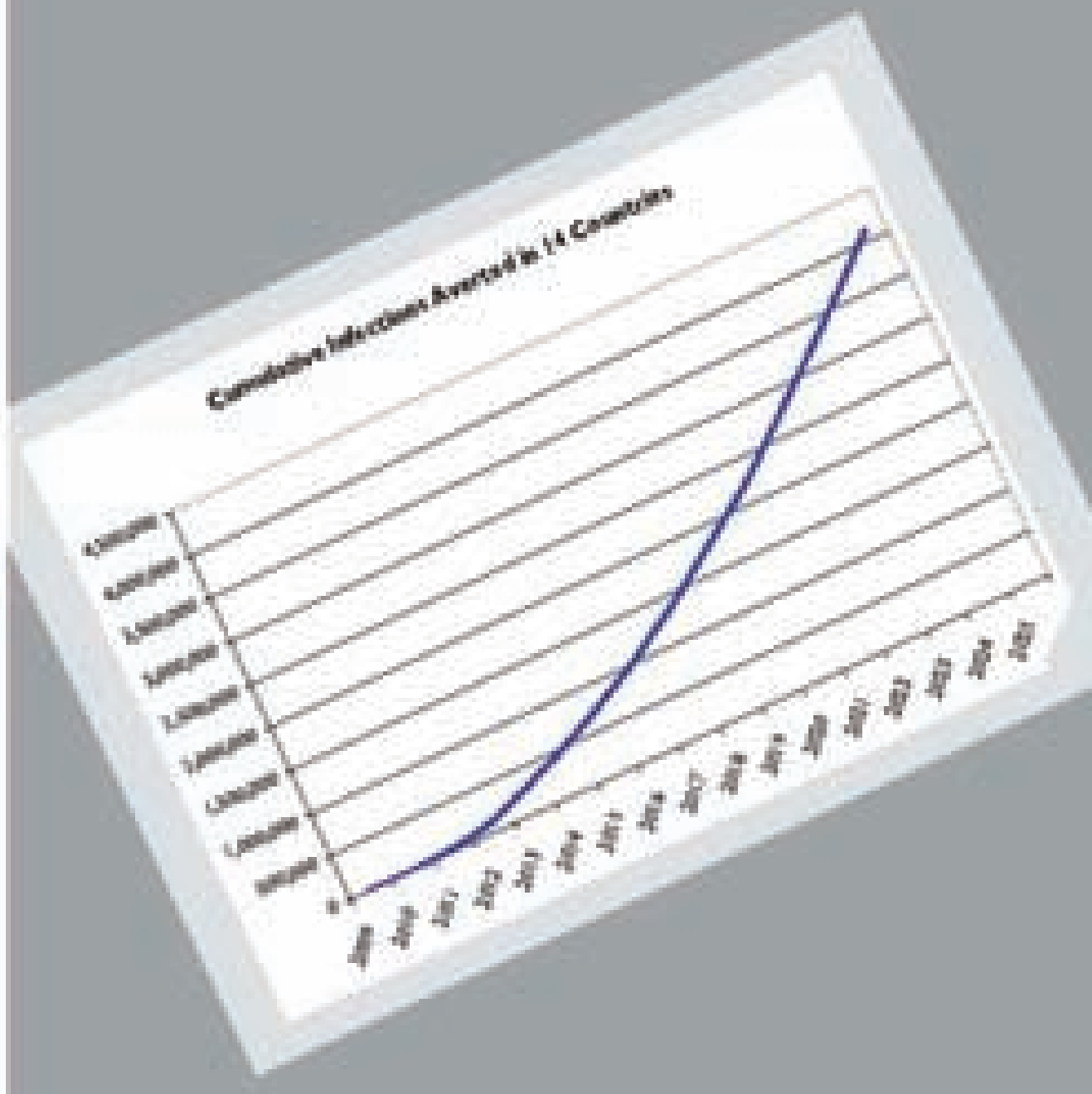

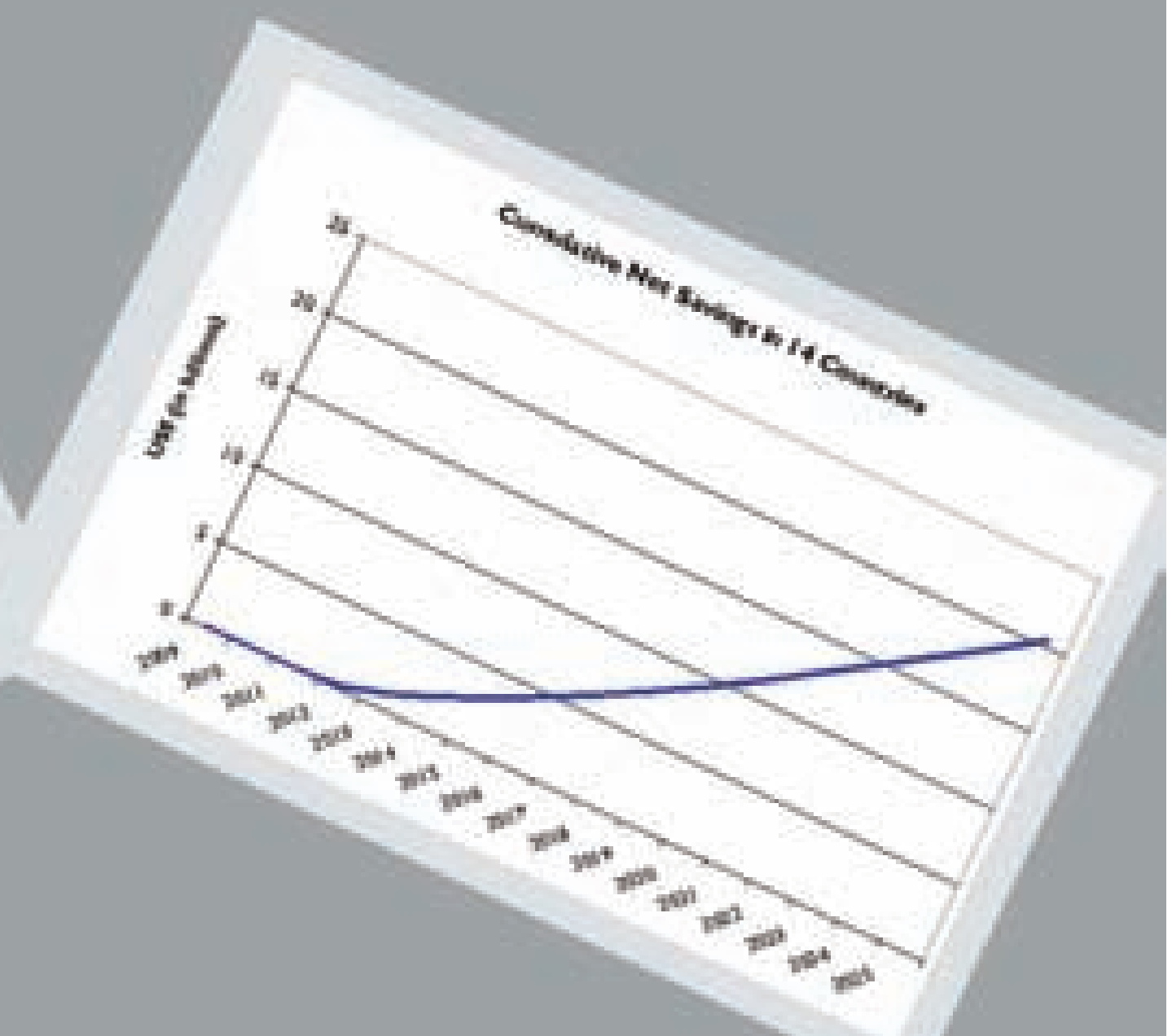

USAID FROM THE AMERICAN PEOPLE | HEALTH POLICY INITIATIVE

UNAIDS
JOINT UNITED NATIONS PROGRAMME ON HIV/AIDS
UNHCR
UNICEF
WFP
UNDP
UNFPA
UNODC
ILO
UNESCO
WHO
WORLD BANK

Suggested citation: Bollinger, L., W. DeCormier Plosky, and J. Stover. 2009. *Male Circumcision: Decision Makers' Program Planning Tool, Calculating the Costs and Impacts of a Male Circumcision Program*. Washington, DC: Futures Group, Health Policy Initiative, Task Order 1.

The USAID | Health Policy Initiative, Task Order 1, is funded by the U.S. Agency for International Development under Contract No. GPO-I-01-05-00040-00, beginning September 30, 2005. The Project's HIV activities are supported by the U.S. President's Emergency Plan for AIDS Relief (PEPFAR). Task Order 1 is implemented by Futures Group, in collaboration with the Centre for Development and Population Activities (CEDPA), White Ribbon Alliance for Safe Motherhood (WRA), and Futures Institute.

UNAIDS is an innovative joint venture of the United Nations, bringing together the efforts and resources of the UNAIDS Secretariat and ten UN system organizations to respond to AIDS. The Secretariat headquarters is in Geneva, Switzerland with staff on the ground in more than 80 countries. Coherent action on AIDS by the UN system is coordinated in countries through the UN theme groups, and the joint programmes on AIDS. Cosponsors include UNHCR, UNICEF, WFP, UNDP, UNFPA, UNODC, ILO, UNESCO, WHO and the World Bank. Visit the UNAIDS web site at http://www.unaids.org

Note:
The graphs on the cover represent calculations made in September 2009, using the DMPPT across 14 countries in Southern and Eastern Africa with standardized cost data and scale-up assumptions.

Male Circumcision: Decision Makers' Program Planning Tool

November 2009

Acknowledgments

The USAID | Health Policy Initiative, Task Order 1, would like to acknowledge John Stover and Lori Bollinger of Futures Institute for developing the Male Circumcision: Decision Makers' Program Planning Tool. The project also expresses its appreciation to Catherine Hankins of the Joint United Nations Programme on HIV/AIDS (UNAIDS), who coordinated a working group (comprising UNAIDS/World Health Organization/South African Centre for Epidemiological Modelling and Analysis) to review the model (*PLoS Med* 6(9): e10000109) and who, with Nicolas Lohse of UNAIDS, organized model applications. The project also thanks Emmanuel Njeuhmeli of USAID, who provided guidance on the application of the model to prepare country briefs.

For questions or further information, please contact:

LBollinger@FuturesInstitute.org, WDeCormier@FuturesInstitute.org, or JStover@FuturesInstitute.org

Futures Institute
41-A New London Turnpike
Glastonbury, CT 06033
USA
(860) 657-5300
http://www.futuresinstitute.org

Table of Contents

1. INTRODUCTION **1**

1.1 STARTING THE PROGRAM 1
1.2 NOTES ON THE MODEL 1
1.3 NOTES ON DATA COLLECTION 2

2. CALCULATING COSTS **3**

2.1 MENU 5
2.2 COUNTRY INFORMATION 5
2.3 FACILITY INFORMATION 6
2.4 SHARE OF FACILITY TIME 6
2.5 COST INPUTS—PERSONNEL 7
2.6 DIRECT COST—PERSONNEL 8
2.7 DIRECT COST—DRUGS & SUPPLIES 9
2.9 DIRECT COST—TRAINING 11
2.11 INDIRECT COSTS—CAPITAL 13
2.12 INDIRECT COSTS—OVERHEADS 14
2.13 UNIT COST CALCULATION 15

3. CALCULATING IMPACTS **17**

3.1 MENU 19
3.2 DEMOGRAPHY 20
3.3 SEXUAL BEHAVIOR 21
3.4 HIV PREVALENCE TRENDS 22
3.5 EFFECTIVENESS OF MALE CIRCUMCISION 23
3.6 EPIDEMIOLOGICAL ASSUMPTIONS 24
3.7 ECONOMIC ASSUMPTIONS 25
3.8 FIT THE MODEL 25
3.9 SPECIFY PRIORITY POPULATION GROUPS AND TARGET COVERAGE LEVELS 26
3.10 SPECIFY SERVICE DELIVERY OPTIONS 27
3.11 VIEW RESULTS 28
3.12 METHODOLOGY 29

1. Introduction

The Male Circumcision: Decision Makers' Program Planning Tool is designed to support policy development and planning for scaling up the provision of safe male circumcision (MC) services. It enables analysts and decision makers to understand the costs and impacts of policy options. It is a part of a toolkit developed by the United Nations Program on HIV/AIDS (UNAIDS)/World Health Organization (WHO) that provides guidelines on comprehensive approaches to male circumcision, including types of surgical procedures and key policy and cultural issues. The main policy areas addressed by the model are the following:

- Priority populations: all male adults, young adults, adolescents, newborns, and most-at-risk groups
- Target coverage levels and rates of scale-up
- Service delivery modes: hospital, clinic, mobile van; public, private, nongovernmental organization, and "other"
- Task shifting

This training manual is designed to introduce participants to the basics of the MC Model, how to set it up, and how to interpret the results.

The MC Model contains two sub-models:

1. The costing model
2. The impact model

The costing part of the model is described first, as the impact model draws on the costing outputs to determine some of the impact outputs.

1.1 Starting the Program

To start the MC Model, open Excel and then select "File," "Open," and "MC Model."

A message similar to the following might appear: "Macros in this workbook are disabled because the security level is high...."

This means that the menu will not work because macros have been disabled. The program can still be used without the menu. However, it is better to allow the menu to operate. To do this, follow these steps:

1. Select "Tools" from the Excel menu.
2. Select "Macros" from the "Tools" menu.
3. Select "Security" from the "Macros" menu.
4. In the tab "Security level," select "Medium."
5. Press "Ok." This will change the security level to allow Excel to run macros that are embedded within Excel spreadsheets.
6. Close the model by selecting "File" and "Close."
7. Open the model again by selecting "File" and "Open."

The menu should now run correctly.

1.2 Notes on the Model

When using the worksheets, note that the cells highlighted in light blue are essential and should be completed. The cells in dark blue contain default data, which can be changed if more accurate information is available. The cells in white cannot be changed.

Also, for display purposes, digits past the hundredth decimal place are not shown for most cells. However, the full number is used in making the calculations.

For the costing worksheets, a red triangle in the corner of a cell signifies a written comment, which can be viewed by placing the cursor over the triangle.

Finally, the examples of the worksheets in the figures below have the data filled in for demonstration purposes; thus, the numbers from an actual model application will likely not mirror those seen in the example figures.

1.3 Notes on Data Collection

The observation of clinical procedures by someone who is not directly involved in providing care requires ethical review to ensure that client privacy is protected. Before beginning data collection for this tool, it is advised to consult local human research protection guidelines and procedures and obtain informed consent from everyone from or about whom data will be collected (i.e., clients, providers, and key informants).

2. Calculating Costs

This section of the manual covers the inputs necessary to estimate the total average cost for a facility to provide services for circumcising an adult male (ages 15–49) or a newborn (age 0–1 month) male. The following inputs will need to be entered for each type of facility in which the circumcision is performed:

- **Facility circumcision share:**
  1. The sum of the adult and newborn circumcision cases per year, divided by
  2. The number of total (equivalent) cases in the facility per year.

  This calculation results in a percentage share for male circumcision, which is used to determine the portion of indirect costs (such as facilities, laboratory equipment, ambulances, hospital administration, etc.) that are attributed to male circumcision.

- **Total percentage of complications:**
  1. It is important to know the complications that can occur with adult or newborn circumcisions and the frequency of their occurrence, as complications add to the cost of the procedure through the need for increased personnel time, drugs, and supplies.
  2. For the purposes of the MC Model, there are four possible complications: hemorrhage, sepsis, other 1, and other 2. Other 1 and other 2 allow for the entering of important country-specific circumcision complications.
  3. The percentage of normal (uncomplicated) circumcisions for adults is calculated by dividing the number of normal adult circumcisions by the total number of adult circumcision cases. The percentage of normal circumcisions for newborns is calculated the same way.
  4. A default value for percentage of circumcisions that hemorrhage is provided, based on published work by USAID to cost male circumcision in Lesotho and Swaziland. Alternatively, the amount (as a number) of circumcisions at that facility that were complicated by hemorrhage can be entered.
  5. Finally, a default value for percentage of circumcisions with sepsis is provided, based on a statement by the American Academy of Pediatrics, which can be changed.
  6. These percentages are used to calculate a weighted average unit cost, including the costs for personnel, drugs, and supplies to treat complications. In this way, the total average cost of an adult or newborn circumcision accounts for the spectrum of normal and complicated circumcisions.

- **Personnel**:
  1. The time needed by each staff member directly involved in pre-operative care, the actual adult or newborn circumcision (with or without complications), and post-operative follow-up, multiplied by
  2. The corresponding salary rate for each directly involved staff member.

- **Drugs and supplies**:
  1. The number of treatment units for each consumable and non-consumable drug or supply needed for an adult or newborn circumcision (with or without complications), multiplied by
  2. The unit cost per consumable or non-consumable drug or supply.

- **Training:**
  1. The total number of training days for all staff needing training (doctors, nurses, and counsellors), multiplied by

2. The cost per staff member per training day to hold the workshop. This includes the costs for supplies, refreshments, and the trainer. If it is anticipated that the training will be held off-site from the circumcision facility, travel and facility costs can be entered.
3. The contribution of training costs to the unit cost for an adult or newborn circumcision is calculated by dividing the annual training costs by the total annual number of circumcisions performed in the facility.

- **Information, education, and communication (IEC) campaign:**
    1. The sum of the costs for each mode of communication used in a national male circumcision education campaign.
    2. This cost is not included in the unit cost calculation but instead is transferred to the impacts section as the "annual demand creation cost" to be added to the total annual net cost of the design of the male circumcision program.

- **Indirect capital costs:**
    1. The purchase price or construction cost of each capital good (such as facility buildings, generators, vehicles, lab equipment) is multiplied by
    2. The facility circumcision share. This product is then divided by the amortization period, or expected duration that the good will last, to get the annualized depreciation value of each capital good.
    3. The contribution of capital cost to the unit cost for an adult or newborn circumcision is calculated by summing the annualized depreciation values for all capital goods and then dividing by the total annual number of male circumcision cases for the facility.

- **Indirect overhead costs:**
    1. For maintenance and utility costs (building and vehicle maintenance and utility costs), the annual cost for each is multiplied by the facility circumcision share to get the estimated cost. The sum of these estimated costs is then divided by the total number of male circumcision cases to obtain the contribution of maintenance and utility costs to the unit cost for an adult or newborn circumcision in that facility.
    2. For support staff (pharmacy techs, housekeeping, etc.) costs, the annual salary, including benefits for each category of support staff, is multiplied by the number of that category staff needed for the facility, and then that product is multiplied by the facility circumcision share. The sum of these labor costs for each category of support staff is then divided by the total annual number of male circumcisions in that facility to get the contribution of support staff salary costs to the unit cost for an adult or newborn circumcision.
    3. The contribution of management and supervision costs to the unit cost for a newborn or adult circumcision is calculated in the same way.

These inputs have been listed in the order in which they appear in the MC Model. They are contained in the following spreadsheets: "Country Information," "Facility Information," "Share of Facility Time," "Cost Inputs—Personnel" and "Direct Cost—Personnel," "Direct Cost—Drugs & Supplies" and "Cost Inputs—Drugs & Supplies," "Direct Cost—Training" (training), "National IEC Campaign," "Indirect Cost—Capital," and "Indirect Cost-Overheads." The "Unit Cost Calculation" spreadsheet sums the total direct and indirect costs for adult or newborn circumcision and then applies the weighting for normal and complicated circumcisions to show the total average cost for an adult or newborn circumcision.

2.1 Menu

Begin the male circumcision costing with the Menu worksheet by clicking on the “Menu” tab at the bottom of the Excel workbook. Below is an example of the Menu worksheet:

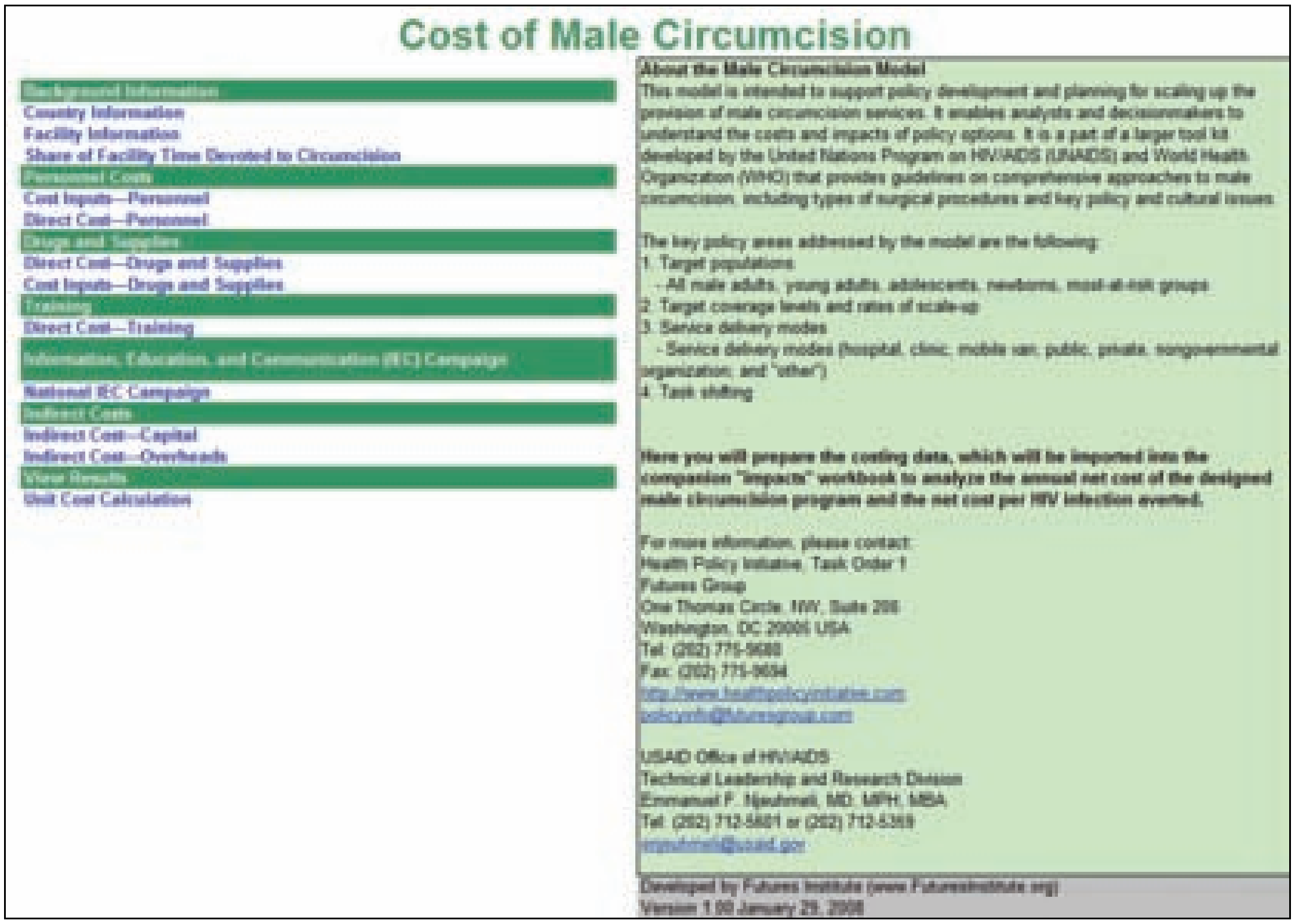

An introduction about the model is on the right, and a menu listing the worksheets in which data must be entered is on the left (categorized by headings in green).

The worksheets for entering data will be listed in blue font. The worksheet title is a hyperlink to the actual worksheet.

2.2 Country Information

Begin by entering data into the Country Information worksheet, which will look like the following:

**Country Information**		Return to Menu
Essential cells that should be completed		
Default data cells that can be changed if information is available		
Country	Swaziland	
Local Currency	E	
Exchange rate	7.32	= 1 US$

1. Enter the name of the country for which the costing will be done.
2. Enter the currency.
3. Enter the exchange rate to U.S. dollars.

Once the information is entered, click on the "Return to Menu" button to be returned to the menu.

2.3 Facility Information

In the next step, choose the type of facility to be costed. This will determine the reference for all of the costing that is to follow. The worksheet will look like the following:

**Facility Information**		Return to Menu
Essential cells that should be completed		
Default data cells that can be changed if information is available		
Name of Facility		
District		
Type/ownership of facility	Public Hospital	

1. From the drop-down menu, choose the type of facility.
2. If preferred, enter in the name of the facility and the district.

All inputs in the workbook will then refer to that specific facility. In the example above, the selected facility type is Public Hospital. A workbook for each type of facility in the MC program should be completed and saved separately.

However, each workbook does not need to be started from scratch. Default data are available for most of the inputs and can be used as a guide. Also, once a workbook for the first type of facility is completed, it can be used as a template to modify as needed for the second type of facility. Save each new workbook with a new name reflecting the facility type.

2.4 Share of Facility Time

Below is an example of the "Share of Facility Time" worksheet:

**Share of Facility Time Devoted to Circumcision**					Return to Menu
Essential cells that should be completed					
Default data cells that can be changed if information is available					
**Facility**					
Number of in-patient days per year	12,000				
Number of out-patient visits per year	60,000				
Equivalency ratio	4.6				
Equivalent cases	115,242				
Number of total circumcision cases per year	270	91%			
Number of adult circumcision cases per year	260				
Number of neonatal circumcision cases per year	10				
Facility circumcision share	0.23%	Adult	%	Neonatal	%
Number of normal (adult/neonatal) circumcisions per year	91.8%	238	91%	10	99%
Number of complications (hemorrhage) per year		6	2.2%	0	0.3%
Number of complications (sepsis) per year		16	6.3%	0	0.3%
Number of complications (other 1) per year		-	0.0%	-	0.0%
Number of complications (other 2) per year		-	0.0%	-	0.0%

Begin by determining the share of facility time devoted to circumcision. Enter in the following:

1. The number of in-patient days per year
2. The number of out-patient visits per year
3. The number of total circumcision cases per year
4. The number of adult circumcision cases per year
5. The number of newborn circumcision cases per year

The facility circumcision share is derived from dividing the number of total circumcision cases per year by the number of equivalent cases in the hospital each year. Given that circumcision is an outpatient procedure, an equivalent case in terms of resources used at the facility is another outpatient procedure or an inpatient procedure that is multiplied by an “equivalency ratio” that translates one in-patient day to a certain number of out-patient visits. In the “Share of Facility Time” worksheet, there is a default equivalency ratio of 4.6—the basis of which can be seen by placing the cursor on the red triangle at the corner of the dark blue box showing 4.6. Therefore, the number of equivalent cases is calculated by multiplying the number of in-patient cases by the equivalency ratio and then adding that to the number of out-patient visits.

Default values for the percent of hemorrhage and sepsis complications for adult and newborn circumcisions are provided; these default values can be overwritten by typing in a different value for the number of complications per year for adult and/or newborn circumcisions.

2.5 Cost Inputs—Personnel

Click on the “Cost Inputs—Personnel” label from the menu page or the “Cost Inputs— Personnel” tab at the bottom of the Excel worksheet. The worksheet will look like the following:

Cost Inputs- Personnel | Return to Menu

Essential cells that should be completed

Default data cells that can be changed if information is available

CLINICAL STAFF SALARY

Personnel Type	Salary per Year	Benefits per Year	Salary (incl. Benefits) per Year	In-Service Training	Salary + In-Service Training	Hours worked per Week	Weeks worked per Year	Cost per Hour Worked
OPD Nurse	$9,843	$984	$10,827	$1,083	$11,910	40	48	$6.20
Counselor	$3,236	$324	$3,560	$356	$3,916	40	48	$2.04
Surgical Nurse	$12,002	$1,200	$13,202	$1,320	$14,522	40	48	$7.56
Nurse anaesthetist	$12,002	$1,200	$13,202	$1,320	$14,522	40	48	$7.56
Nurse assistant	$3,236	$324	$3,560	$356	$3,916	40	48	$2.04
Auxiliary//Attendant	$3,236	$324	$3,560	$356	$3,916	40	48	$2.04
Anaesthetist	$14,634	$1,463	$16,098	$1,610	$17,708	40	48	$9.22
Physician	$14,634	$1,463	$16,098	$1,610	$17,708	40	48	$9.22
Clinical Officer	$14,634	$1,463	$16,098	$1,610	$17,708	40	48	$9.22
Consultant Urologist	$14,634	$1,463	$16,098	$1,610	$17,708	40	48	$9.22
Lab technician	$12,002	$1,200	$13,202	$1,320	$14,522	40	48	$7.56
Other 1	$0	$0	$0	$0	$0	40	48	$0.00
Other 2	$0	$0	$0	$0	$0	40	48	$0.00
Other 3	$0	$0	$0	$0	$0	40	48	$0.00
Other 4	$0	$0	$0	$0	$0	40	48	$0.00

1. Select the personnel type from the drop-down menu in the “Personnel Type” column.
2. Enter the annual salary for each of the staff categories.

The MC Model assumes that benefits are 10 percent of salary costs, that the cost of in-service training is 10 percent of the staff salary with benefits, and that each person works 40 hours per week for 48 weeks per year. These values can be changed directly in the workbook.

It is here and in the following worksheet that the issue of task shifting can be addressed. The mix of personnel involved in the circumcision can be chosen by selecting different configurations of personnel categories. Correspondingly, adjust the time used by each personnel type in the "Cost Inputs—Personnel" worksheet. **Then, for each task-shifting scenario generated, save the workbook and name it accordingly**. For instance, in the Public Hospital example, create file names such as "Public Hospital, no task shifting" and "Public Hospital, task shifting physician to clinical officer."

2.6 Direct Cost—Personnel

Go to the "Direct Cost—Personnel" worksheet. The portion of the spreadsheet to review and/or enter data will look like the following:

Direct Cost- Personnel — Return to Menu

Essential cells that should be completed

Default data cells that can be changed if information is available

PERSONNEL TIME REQUIREMENTS PER INDIVIDUAL INTERVENTION (IN MINUTES)

Personnel Type	Pre-circumcision	Circumcision	Neonatal Circumcision	Post-Circumcision - Normal	Complication - Hemorrhage	Complication - Sepsis	Complication - Other 1	Complication - Other 2	Circumcision Follow-up	Number of hours needed per normal circumcision	Number of hours needed per normal neonatal circumcision
**FACILITY**											
OPD Nurse										--	--
Counselor	20.00									0.33	--
Surgical Nurse	8.00	25.00	25.00		10.00	20.00				0.55	0.42
Nurse anaesthetist		20.00	20.00		10.00					0.33	0.33
Nurse assistant		25.00	25.00	8.00					8.00	0.68	0.55
Auxiliary//Attendant					5.00					--	--
Anaesthetist										--	--
Physician	5.00	25.00	25.00	8.00	10.00					0.63	0.55
Lab technician	10.00									0.17	--
Other 1										--	--

STAFF UNIT COST CALCULATION

Personnel Type	Cost per Hour Worked	Staff Unit cost per normal circumcision	Staff Unit cost per normal neonatal circumcision	Staff Unit cost per circumcision w hemorrhage	Staff Unit cost per neonatal circumcision w hemorrhage	Staff Unit cost per per circumcision w sepsis	Staff Unit cost per per neonatal circumcision w sepsis	Staff Unit cost per per per circumcision w other 1	Staff Unit cost per per per neonatal circumcision w other 1	Staff Unit cost per per per circumcision w other 2	Staff Unit cost per per per neonatal circumcision w other 2
OPD Nurse	$6.20	-	-	-	-	-	-	-	-	-	-
Counselor	$2.04	0.68	-	0.68	-	0.68	-	0.68	-	0.68	-
Surgical Nurse	$7.56	4.16	3.15	5.42	4.41	6.68	5.67	4.16	3.15	4.16	3.15
Nurse anaesthetist	$7.56	2.52	2.52	3.78	3.78	2.52	2.52	2.52	2.52	2.52	2.52
Nurse assistant	$2.04	1.39	1.12	1.39	1.12	1.39	1.12	1.39	1.12	1.39	1.12
Auxiliary//Attendant	$2.04	-	-	0.17	0.17	-	-	-	-	-	-
Anaesthetist	$9.22	-	-	-	-	-	-	-	-	-	-
Physician	$9.22	5.84	5.07	7.38	6.61	5.84	5.07	5.84	5.07	5.84	5.07
Lab technician	$7.56	1.26	-	1.26	-	1.26	-	1.26	-	1.26	-
Other 1	$0.00	-	-	-	-	-	-	-	-	-	-
		$15.9	$11.9	$20.1	$16.1	$18.4	$14.4	$15.9	$11.9	$15.9	$11.9

1. Select the personnel type from the drop-down menu in the "Personnel Type" column.

Default values for the number of minutes required by each staff member for pre-operative counselling, the circumcision, and any post-operative care are listed. Review these default values, and make any changes as necessary.

Notice that the number of hours needed to perform an adult or newborn circumcision with or without complications and the total staff time requirements calculations will be done automatically by the excel program, based upon the number of minutes of personnel time listed in the cells that are dark blue.

The “Direct Cost—Personnel” worksheet also draws from the previous sheet on “Cost Inputs—Personnel” to calculate the staff unit cost per normal or complicated adult or newborn circumcision. The “Cost Inputs—Personnel” worksheet calculates each staff member’s hourly rate (including benefits and training), which is then multiplied in the “Direct Cost—Personnel” worksheet by the number of hours needed to perform an adult or newborn circumcision with or without complications to calculate the staff unit cost.

2.7 Direct Cost—Drugs & Supplies

Go to the “Direct Cost—Drugs & Supplies” worksheet. Below is an excerpt from that worksheet:

Direct Costs- Drugs & Supplies Return to Menu

Essential cells that should be completed

Default data cells that can be changed if information is available

Intervention	Description of treatment line	Percent of intervened receiving this line at Facility	Number	Times per day	Days per case/ episode	Treatment units per case/ episode	Unit cost	Cost per case or episode	Average cost per patient at Facility
**CONSUMABLES**									
**Pre-circumcision**									
Pre-circumcision	Rapid test	100%	1	1	1	1	$2.46	$2.46	$2.46
Pre-circumcision	Gloves, examination, non-sterile, disposable, pair	100%	1	1	1	1	$0.05	$0.05	$0.05
**Circumcision**									
Circumcision	Gloves, surgeons, sterile disposable, pair	100%	4	1	1	4	$0.21	$0.83	$0.83
Circumcision	Gloves, examination, non-sterile, disposable, pair	100%	2	1	1	2	$0.05	$0.10	$0.10
Circumcision	Surgical Mask	100%	3	1	1	3	$0.04	$0.11	$0.11
Circumcision	Surgical cap (disposable)	100%	3	1	1	3	$0.33	$0.98	$0.98
Circumcision	Goggles (for surgery)	0%	0	1	1	0	$0.00	$0.00	$0.00
Circumcision	Surgical scrub Betadine	100%	30	1	1	30	$0.02	$0.55	$0.55
Circumcision	Hibiscrub	0%	0	1	1	0	$0.00	$0.00	$0.00
Circumcision	Salvalon	0%	0	1	1	0	$0.01	$0.00	$0.00
Circumcision	Povidone antiseptic solution	100%	40	1	1	40	$0.00	$0.15	$0.15
Circumcision	Gauze pad, sterile, 8ply 100x100mm	100%	12	1	1	12	$0.01	$0.08	$0.08
Circumcision	Gauze pad, sterile, 12ply 76x76mm	0%	0	1	1	0	$0.01	$0.00	$0.00
Circumcision	Lignocaine 2%, injection 20ml	100%	20	1	1	20	$0.12	$2.36	$2.36
Circumcision	Needle 21 guage	100%	1	1	1	1	$0.03	$0.03	$0.03
Circumcision	Needle 18 guage	100%	1	1	1	1	$0.03	$0.03	$0.03
Circumcision	Syringe, 10ml, disposable	100%	1	1	1	1	$0.03	$0.03	$0.03
Circumcision	Ketamine, injection 50mg/ml	0%	0	1	1	0	$0.12	$0.00	$0.00
Circumcision	Thiopental, injection 1g + diluent	0%	0	1	1	0	$0.00	$0.00	$0.00
Circumcision	IV canula (Jelo radiopaque) 18 guage	0%	0	1	1	0	$1.67	$0.00	$0.00
Circumcision	Glucose 5% in water + set, 500ml	0%	0	1	1	0	$0.96	$0.00	$0.00
Circumcision	Blade, 22, 23 or 24	100%	1	1	1	1	$0.02	$0.02	$0.02
Circumcision	Suture, catgut chromic 3/0, 150cm	0%	0	1	1	0	$7.24	$0.00	$0.00
Circumcision	Suture, catgut chromic 4/0, 150cm	100%	1	1	1	1	$0.68	$0.68	$0.68
Circumcision	Suture, silk, 2 x 0.75m	0%	0	1	1	0	$5.11	$0.00	$0.00
Circumcision	Gauze pad, Jelonet (paraffine vaseline)	0%	0	1	1	0	$0.17	$0.00	$0.00
Circumcision	Gauze pad, Inadine	100%	1	1	1	1	$0.17	$0.17	$0.17
Circumcision	Elastoplast/Dermaplast 25mm/9m	50%	10	1	1	5	$0.17	$0.85	$0.43
Circumcision	Micropore	50%	10	1	1	5	$0.17	$0.85	$0.43
Circumcision	Tegaderm (3M) 1624	0%	0	1	1	0	$4.38	$0.00	$0.00
Circumcision	Bandage (elastic)	5%	1	1	1	0.05	$0.06	$0.00	$0.00
Circumcision	Iboprufen, Brufen 400mg	0%	1	3	5	0	$0.00	$0.00	$0.00
Circumcision	Paracetamol, tablets 500mg	100%	1	3	7	21	$0.04	$0.82	$0.82
Circumcision	Amoxycillin, 500mg	0%	0	3	7	0	$0.01	$0.00	$0.00
Circumcision	Cloxacillin, 500mg	0%	0	4	7	0	$0.04	$0.00	$0.00
**Neonatal Circumcision**									
Neonatal Circumcision	Gloves, surgeons, sterile disposable, pair	100%	4	1	1	4	$0.21	$0.83	$0.83
Neonatal Circumcision	Gloves, examination, non-sterile, disposable, pair	100%	2	1	1	2	$0.05	$0.10	$0.10
Neonatal Circumcision	Surgical Mask	100%	3	1	1	3	$0.04	$0.11	$0.11

In this worksheet, all of the necessary input information has default values, displayed in the dark blue cells. To calculate the average cost per patient for each of the drugs and supplies used in a circumcision, it is necessary to know:

1. The percent of cases receiving that drug or supply
2. The number of that drug or supply needed per case
3. The number of times per day that drug or supply needs to be taken or used
4. The number of days for which that drug or supply needs to be used during the procedure and for follow-up

The worksheet is broken down into consumable and non-consumable drugs and supplies and further broken down by adult or newborn circumcision and normal or complicated circumcision.

The “Direct Costs—Drugs & Supplies” worksheet draws the unit cost per drug or consumable from the “Cost Inputs—Drugs & Supplies” worksheet to calculate the average cost per patient for each drug and supply used for a circumcision.

2.8 Cost Inputs—Drugs & Supplies

Go to the “Cost Inputs—Drugs & Supplies” worksheet. The following is an excerpt from that worksheet:

Cost Inputs- Drug & Supplies Return to Menu

Essential cells that should be completed

Default data cells that can be changed if information is available

Drug or Supply, form, strength	Supplier	Stock Number	Pack Price (local currency)	Pack Price (US$)	Pack Price (in US$)	Units per Pack	Unit Cost
Amoxycillin, 500mg			52.71		$7.20	500	$0.01
Ampicillin, tablets 500mg					$0.00	100	$0.00
Bandage (elastic)			4.90		$0.67	12	$0.06
Blade, 22, 23 or 24			18.00		$2.46	100	$0.02
Cloxacillin, 500mg			142.08		$19.41	500	$0.04
Elastoplast/Dermaplast 25mm/9m			125.12		$17.09	100	$0.17
Erythomycin, capsule 250mg			220.00		$30.05	100	$0.30
Gauze pad, sterile, 12ply 100x100mm			6.36		$0.87	100	$0.01
Gauze pad, sterile, 8ply 100x100mm			4.74		$0.65	100	$0.01
Gauze pad, sterile, 12ply 76x76mm			4.74		$0.65	100	$0.01
Gauze pad, Inadine			12.75		$1.74	10	$0.17
Gauze pad, Jelonet (paraffine vaseline)			12.75		$1.74	10	$0.17
Gloves, surgeons, sterile disposable, pair			76.00		$10.38	50	$0.21
Gloves, examination, non-sterile, disposable, pair			18.90		$2.58	50	$0.05
Glucose 5% in water + set, 500ml			7.00		$0.96	1	$0.96
Goggles (for surgery)					$0.00	1	$0.00
Hibiscrub			135.00		$18.44	5,000	$0.004
Hibitane (1L)			135.00		$18.44	5,000	$0.004
Iboprufen, Brufen 400mg					$0.00	1	$0.00
Ketamine, injection 50mg/ml			8.52		$1.16	10	$0.12
Lignocaine 2%, injection 20ml			17.29		$2.36	20	$0.12
Micropore			125.12		$17.09	100	$0.17
Needle 18 guage			19.85		$2.71	100	$0.03
Needle 21 guage			19.30		$2.64	100	$0.03
Surgical Mask			13.00		$1.78	50	$0.04
Paracetamol, tablets 500mg			28.65		$3.91	100	$0.04
Paracetamol, syrup							$0.50
Povidone antiseptic solution			135.00		$18.44	5,000	$0.004
Saline					$0.00	1	$0.00
Salvalon			79.89		$10.91	2,000	$0.01
Surgical cap (disposable)			23.80		$3.25	10	$0.33
Surgical scrub Betadine			135.00		$18.44	1,000	$0.02
Suture, catgut chromic 4/0, 150cm			59.88		$8.18	12	$0.68
Suture, catgut chromic 3/0, 150cm			52.97		$7.24	1	$7.24
Suture, catgut plain 2/0, 150cm			48.09		$6.57	1	$6.57
Suture, silk, 2 x 0.75m			37.38		$5.11	1	$5.11
Suture, silk, 2 x 0.75m					$0.00	1	$0.00
Syringe, 10ml, disposable			22.95		$3.14	100	$0.03
Syringe, 5ml, disposable			15.38		$2.10	1	$2.10
Syringe, 2ml, disposable			13.75		$1.88	1	$1.88
Tegaderm (3M) 1624			32.04		$4.38	1	$4.38
Thiopental, injection 1g + diluent					$0.00	1	$0.00
**Non-consumable supplies**							
Surgical gown			128.15		$17.51	1	$17.51
Stretch pants medium			123.49		$16.87	1	$16.87
Stretch pants large			128.15		$17.51	1	$17.51
Shoe covers			17.15		$2.34	50	$0.05

In this worksheet, enter the following:

1. The price per pack in local currency or in US$. Note that the MC Model is set up to accept the price in local currency (which is converted to US$ by the exchange rate) over US$, if both are entered for the same good.
2. The units per pack.

The unit cost is then calculated by dividing the price per pack (in US$) by the units per pack.

2.9 Direct Cost—Training

Go to the “Direct Cost—Training” worksheet. The worksheet looks like the one below:

Direct Cost- Training	Return to Menu
Essential cells that should be completed	
Default data cells that can be changed if information is available	
**Training of surgical staff**	
Number of training days (doctors)	1
Number of training days (nurses)	2
Number of doctors to be trained	1
Number of nurses to be trained	1
Number of staff days (doctors)	1
Number of staff days (nurses)	2
**Cost of workshop**	
Cost per person per training day	$68
Number of training days	3
subtotal	$205
**Cost of staff time (included in salary costs)**	
Training time already included in salary cost?	Yes
Hours of Doctors time	8
Hours of Nurses time	16
Cost of doctors staff time	$0
Cost of nurses staff time	$0
subtotal	$0
**Total**	**$205**
**Training of counselors**	
Number of training days (counselors)	3
Number of counselors to be trained	1
Number of staff days (counselors)	3
**Cost of workshop**	
Cost per person per training day	$68
Number of training days	3
subtotal	$205
**Cost of staff time**	
Training time already included in salary cost?	Yes
Hours of counselors time	24
Cost of counselors staff time	$0
**Total**	**$205**
Annual Training Costs	**$410**
Contribution of Training Costs to Unit cost	**$1.52**

Enter in the following:

1. The number of training days for doctors
2. The number of training days for nurses
3. The number of training days for counselors
4. The number of doctors to be trained
5. The number of nurses to be trained
6. The number of counselors to be trained

7. The cost per person per training day [This will be the same for doctors/nurses and for counselors, as it is the cost for holding the training (refreshments, office supplies, facilitator, etc). If the training will be held off-site from the facility where the circumcision takes place, facility rental and travel costs will have to be factored into the cost per person per training day.]

Next, choose if the training time is already included in the salary cost:

1. Click on the box next to “training time already included in staff cost,” which is highlighted in light blue.
2. Choose “yes” or “no” from the drop-down menu.
3. If you choose “no,” the cost of the training time for doctors, nurses, and counselors will be drawn by the MC Model from the previous worksheet.

2.10 National IEC Campaign

Click on the “National IEC Campaign” label in the Menu page or the “National IEC Campaign” tab at the bottom of the Excel Workbook to access a worksheet that will look like the following:

National IEC Campaign	Return to Menu
Essential cells that should be completed	
Default data cells that can be changed if information is available	
Graphic Design of pamphlet	$273.22
Printed Material (per pamphlet)	$0.41
Number of pamphlets printed	10,000
Pamphlet sub-total	$4,371.58
Visual - 1 Billboards	$6,830.60
Number of billboards per year	2
Billboard sub-total	$13,661.20
Television Ad (production fee)	$10,000.00
Television Ad (30 sec airtime slot)	$95.90
Number of television slots per year	0
Television sub-total	$0.00
Newspaper (1/4 page ad/week)	$185.11
Number of weeks newspaper ad runs	4
Newspaper sub-total	$740.44
Radio (production fee)	$27.32
Radio (30 sec airtime slot)/day	$32.24
Number of radio slots per year	50
Radio sub-total	$1,639.34
Press Kit (color pages in gloss w/ folder)	$3.14
Number of press kits	50
Press kit sub-total	$157.10
**Total**	**$20,569.67**

Enter (in US$ when applicable) the following:

1. Cost of graphic design of the pamphlet
2. Cost of printed material (per pamphlet)
3. The number of pamphlets printed
4. Cost of the billboards
5. The number of billboards
6. Production fee for the television ad

7. Cost of a 30-second slot of television airtime
8. The number of 30-second slots needed
9. Cost of a ¼ page newspaper ad per week
10. The number of weeks the ad will run
11. Production fee for radio ad
12. Cost of a 30-second slot of radio airtime
13. The number of 30-second slots needed
14. Cost of a press kit
15. The number of press kits made

2.11 Indirect Costs—Capital

Go to the "Indirect Costs—Capital" worksheet to find a spreadsheet similar to the following:

Indirect Cost- Capital (annualized) — Return to Menu

Essential cells that should be completed

Default data cells that can be changed if information is available

Hospital

Capital Cost	Amortization period (years)	Estimated construction cost/ purchasing price	Male Circumcision Share	Annualized depreciation
Autoclave	7	*$15,574*	0.23%	$5.21
Beds	7	*$204,918*	0.23%	$68.59
Communications Equipment	2		0.23%	$0.00
Dental Equipment	7		0.23%	$0.00
Examination & Surgery Equipment	5	*$533,141*	0.23%	$249.82
Facilities	25	*$1,000,000*	0.23%	$93.72
Generator and Incinerator	7		0.23%	$0.00
Laboratory Equipment	7	*$161,352*	0.23%	$54.00
Office Furniture	7		0.23%	$0.00
Other	7	*$52,919*	0.23%	$17.71
Pharmacy Equipment	7		0.23%	$0.00
Phisiotherapy Equipment	7		0.23%	$0.00
Radiology-Imaging Equipment	7		0.23%	$0.00
Refrigerators, Coolers	7	*$13,251*	0.23%	$4.44
Vehicles - 4 wheel drive	5	*$38,074*	0.23%	$17.84
Vehicles - Ambulance	5	*$30,697*	0.23%	$14.38
Vehicles - Car	5	*$8,197*	0.23%	$3.84
Vehicles - Van	5	*$17,760*	0.23%	$8.32
Vehicles - Minivan	5	*$46,045*	0.23%	$21.58
**CAPITAL COSTS PER FACILITY**		**$2,121,927**		**$559**
Contribution of Capital cost to Unit cost				**$2.07**

Enter the following:

1. Estimated construction cost or purchasing price for each category of capital goods

Then review the default values for the amortization period of each category of capital goods and make any changes if more accurate information is available. The annualized depreciation of each category of capital goods is calculated by the MC Model by multiplying the construction cost or purchasing price by the male circumcision share and then dividing that product by the amortization period. The sum of the annualized depreciation values for each category of capital goods is then divided by the total annual number of circumcisions performed in the facility to arrive at the contribution of capital cost to the unit cost for an adult or newborn circumcision.

2.12 Indirect Costs—Overheads

Click on the "Indirect Costs—Overheads" tab at the bottom of the Excel workbook to find the following worksheet:

Indirect Cost—Overheads — Return to Menu

Essential cells that should be completed

Default data cells that can be changed if information is available

MAINTENANCE AND UTILITY COSTS

Staff category	Annual cost (percent of new value)	Costs	Facility<br>Male Circumcision Share	Estimated cost
Building Maintenance Cost	*100%*	$128,240	0.23%	$300
Utilities Cost	*100%*	$128,240	0.23%	$300
Emergency Vehicle Maintenance	*100%*	$100,788	0.23%	$236
**MAINTENANCE AND UTILITY COSTS PER FACILITY**				**$837**
Contribution of Maintenance and Utility Cost to Unit cost				**$3.1**

SUPPORT PERSONNEL

Staff category	Salary per year	Benefits per Year	Facility<br>Salary (incl. Benefits) per Year	Required FTE per facility	Male Circumcision Share	Labor costs
Senior pharmacy technician	*$9,843*	*$984*	$10,827	*1*	0.23%	$25
Pharmacy technician	*$6,928*	*$693*	$7,621	*2*	0.23%	$36
Lat technologist	*$6,928*	*$693*	$7,621	*1*	0.23%	$18
Assistant HR officer	*$6,928*	*$693*	$7,621	*1*	0.23%	$18
Technical officer	*$6,928*	*$693*	$7,621	*3*	0.23%	$54
Senior health assistant	*$4,887*	*$489*	$5,376	*1*	0.23%	$13
Health assistant	*$3,236*	*$324*	$3,560	*2*	0.23%	$17
Radiographic assistant	*$3,236*	*$324*	$3,560	*0*	0.23%	$0
Lab assistant	*$3,236*	*$324*	$3,560	*0*	0.23%	$0
Driver	*$2,146*	*$215*	$2,360	*5*	0.23%	$28
Accountant	*$4,887*	*$489*	$5,376	*1*	0.23%	$13
Assistant accountant	*$3,236*	*$324*	$3,560	*5*	0.23%	$42
Accounts clerk	*$1,498*	*$150*	$1,648	*0*	0.23%	$0
Clerical assistant	*$1,498*	*$150*	$1,648	*4*	0.23%	$15
Ward attendant	*$1,498*	*$150*	$1,648	*7*	0.23%	$27
Hospital assistant	*$1,248*	*$125*	$1,372	*32*	0.23%	$103
Office assistant	*$1,248*	*$125*	$1,372	*18*	0.23%	$58
Guard	*$0*	*$0*	$0		0.23%	$0
Housekeeping	*$0*	*$0*	$0		0.23%	$0
Reception	*$0*	*$0*	$0		0.23%	$0
Records	*$0*	*$0*	$0		0.23%	$0
Supply clerk	*$0*	*$0*	$0		0.23%	$0
Maintenance	*$0*	*$0*	$0		0.23%	$0
Mgmt officer	*$0*	*$0*	$0		0.23%	$0
Food preparer	*$0*	*$0*	$0		0.23%	$0
Porter	*$1,248*	*$125*	$1,372	*6*	0.23%	$19
**TOTAL SUPPORT SALARY COSTS PER FACILITY**						**$484**
Contribution of Salary Cost to Unit cost						**$1.8**

MANAGEMENT/SUPERVISION COSTS

Staff category	Salary per year	Benefits per Year	**Hospital**<br>Salary (incl. Benefits) per Year	Required FTE per facility	Male Circumcision Share	Labour costs
District Hospital Administrator	*$14,634*	*$1,463*	$16,098	*1*	0.23%	$38
Health Inspector	*$12,002*	*$1,200*	$13,202	*2*	0.23%	$62
Asst Health Inspector	*$0*	*$0*	$0	*0*	0.23%	$0
**SUBTOTAL**						$100
Travel Expenses (Transportation, accomodation, etc.)						$0
**SUPERVISION COST PER FACILITY**						**$100**
Contribution of Supervision Cost to Unit Cost						**$0.37**

Enter (in US$ when applicable) the following:

1. Annual maintenance and utility costs
2. The salary per year for each category of support staff
3. The required full-time equivalent for each category of support staff
4. The salary per year for each category of management/supervision staff
5. The full-time equivalent for each category of management/supervision staff

2.13 Unit Cost Calculation

Click on the "Unit Cost Calculation" label on the Menu page or tab of that name at the bottom of the Excel workbook to review the summary of the calculations from the previous worksheets. The summary will look like the following:

	Return to Menu			
Essential cells that should be completed				
Default data cells that can be changed if information is available				
**UNIT COST CALCULATION (WITH COMPLICATIONS weighted average)**				
**Direct costs**	**Adult**		**Newborn**	
Consumables costs	$11.89	32%	$9.67	32%
Non-consumable supplies costs	$0.23	1%	$0.22	1%
Personnel costs	$16.11	43%	$11.89	39%
Training costs	$1.52	4%	$1.52	5%
Subtotal	*$29.74*	80%	*$23.30*	76%
**Indirect costs**	**Adult**		**Newborn**	
Capital costs	$2.07	6%	$2.07	7%
Maintenance and utility costs	$3.10	8%	$3.10	10%
Support personnel costs	$1.79	5%	$1.79	6%
Management and supervision costs	$0.37	1%	$0.37	1%
Subtotal	*$7.33*	20%	*$7.33*	24%
**TOTAL**	$37.07	100%	$30.64	100%
	1.4%	$ 0.50		
**UNIT COST CALCULATION (NO COMPLICATIONS)**				
**Direct costs**	**Adult**		**Newborn**	
Consumable supplies costs	$11.63	32%	$9.63	32%
Non-consumable supplies costs	$0.23	0.6%	$0.23	0.7%
Personnel costs	$15.86	43%	$11.87	39%
Training costs	$1.52	4%	$1.52	5%
Subtotal	*$29.23*	*80%*	*$23.24*	*76%*
**Indirect costs**	**Adult**		**Newborn**	
Capital costs	$2.07	6%	$2.07	7%
Maintenance and utility costs	$3.10	8%	$3.10	10%
Support personnel costs	$1.79	5%	$1.79	6%
Management and supervision costs	$0.37	1%	$0.37	1%
Subtotal	*$7.33*	*20%*	*$7.33*	*24%*
**TOTAL**	$36.57	100%	$30.58	100%

No data entry is needed on this worksheet, but it is useful to look at it closely to see where the majority of costs are and the comparisons in cost between normal and complicated circumcisions for an adult or newborn.

The total cost cells that are highlighted in orange and with red font indicate the total average cost for an adult or newborn circumcision at the type of facility you chose for reference. These are the main outputs for the costing section of the MC Model.

These totals are transferred to the impact model for use in calculating the total net cost of the program and the net cost per infection averted.

3. Calculating Impacts

This section is used to calculate the impact of a male circumcision program on an HIV/AIDS epidemic and the associated cost per infection averted. The impact model requires several pieces of information, including the following (where the subscript "x" denotes the younger age group and the subscript "y" denotes the older age group):

- **Country-Specific Data: Demography**
    1. Demographic data are used for population trends that capture the numbers of individuals who are, and will be, newborns or in the sexually active age group of 15–49 that is most at risk for contracting HIV. The data series includes adult population ages 15–49, adult population growth rate, crude birth rate, crude death rate_x, crude death rate_y, and proportion surviving to age 15.
    2. Demographic data are also used to break down the population trends by gender and age group to make projections according to the target populations for a male circumcision program. The data series includes male population_x as a proportion of the 15–49 group, male population age 15 as a proportion of the 15–49 group, male population age 49 as a proportion of the 15–49 group, female population_x as a proportion of the 15–49 group, male population age 15 as a proportion of the 15–49 group, and proportion of the female adult population.
    3. Current prevalence of male circumcision shows the amount of unmet need for male circumcision in the country.

- **Country-Specific Data: Sexual Behavior**
    1. The proportion of sexual contacts is broken down by gender and age of the sexual partner from age 15 to the upper age of group x, as well as from the lower age of group y to age 49. This specifies a sexual mixing matrix that models when each gender and age group is potentially contracting HIV.
    2. Risk compensation is the percent of behavior change that is reversed when circumcision occurs.

- **Country-Specific Data: HIV Prevalence Trends**
    1. Adult HIV prevalence gives a measure of the scale of the epidemic with the current rate of male circumcision, which is combined with demographic and sexual behavior data to predict the number of new infections.
    2. The HIV prevalence from a population survey that is broken down by gender and age group enables the model to estimate new infections by gender and age group.

- **Review or Revise Epidemiological and Economic Assumptions: Effectiveness of Male Circumcision**
    1. From the literature, an estimate of the reduction in probability of a man who has been circumcised becoming infected with HIV by a female is given for the general population and for the most-at-risk population. This is used to calculate infections averted.
    2. The calculated rates for force of infection (defined below) for those not circumcised are also given by gender and age group and come from the "Fit the Model" section, so they should not be revised. Force of infection is multiplied by prevalence to calculate the proportion of uninfected that becomes infected each year.

- **Review or Revise Epidemiological and Economic Assumptions**: **Epidemiological Assumptions**
    1. Default values for the probability of transmission from mother to child and the fertility reduction due to HIV are given, which are combined with the population trends to calculate the population at risk for contracting HIV through sex and the potential for infections to be averted by male circumcision.
    2. The parameters for survival function are also given, which are used to create a Weibull distribution describing the years since infection with HIV and the cumulative proportion of those infected who have died. This output gives information on how long an infected person lives and can potentially transmit the virus.
    3. The default value for the time step for model calculations is set at .1, so that the model calculates information for each 1/10 of a year. In an epidemic with changing behavior and prevalence trends, calculating the model on a yearly basis is likely not accurate enough. However, calculating at a rate of less than 1/10 of a year is more time consuming and cumbersome for the computer.

- **Review or Revise Epidemiological and Economic Assumptions: Economics Assumptions**
    1. The default value for the discount rate on future expenditures and savings is 3 percent. This rate is used to calculate future expenditures and savings in terms of today's dollars.
    2. The default value of the discounted lifetime cost of antiretroviral therapy (ART)) is US$7,400. This is the savings gained from not having to provide ART for the time that a patient would have needed them, because that patient was circumcised and did not contract HIV. This information is used to calculate the net savings per infection averted.

- **Fit the Model to the Prevalence Trends: Fit the Model**
    1. The "Fit the Model" worksheet is used primarily to determine the force of infection rates by gender and age group. The model uses all of the input data to create an HIV prevalence curve for the general adult population. Based on this curve and any input from a national survey for a single recent year for HIV prevalence by age and gender group, using the Solver function in Excel creates four additional curves: males in the young age group, females in the young age group, males in the older age group, and females in the older age group.
    2. The age/gender curves may not align with the points that represent the data from the national survey(s), as some of the inputs such as sexual behavior and probability of transmission from mother to child are best estimates. To get a better alignment—so that all age/gender curves pass through the national survey points as closely as possible—some of the curve parameters can be manually entered: "r" representing the force of infection (or how steeply the curve rises), "alpha" representing the plateau level of the epidemic, and "e" representing the rate of decline after the peak of the epidemic (or how steeply the curve falls after the plateau).
    3. The sum of squared errors shows how close one is to the data values created by the model from the analysis of all inputs, given that when fitting the model manually, only one or maybe two actual data points are used. Thus, if manually fitting the model, in addition to trying to match the national survey data points, the sum of squared errors should also be minimized. Once a good fit is obtained, the force of infection rate will be taken from the determined model parameters.

- **Set Policy Options: Specify Priority Population Groups and Target Coverage Levels**
    1. Determine what the program will look like by choosing the priority groups to cover: all adult males, 15–24 year old males, adolescent males prior to sexual debut, newborn

males, most-at-risk males, and/or "other" risk groups. Set the year when the male circumcision program starts.

2. For each priority group, verify the percent circumcised in the current year, the target for future percent circumcised, the target year for program completion, and the pace of scale-up. These variables will shape how quickly and broadly the program is implemented. The impact on the number of future infections averted can then be determined.
3. Note that, if targeting a sub-population like most-at-risk males, the proportion of this group out of all males ages 15–49 must be specified.

- **Set Policy Options: Specify Service Delivery Options**
  1. Break down the male circumcision program by the service delivery mode, as service delivery modes may have different costs.
  2. Specify the user fee for each delivery mode, if desired.
  3. The cost per circumcision is transferred to this page from the costing worksheet. Together, this information is used to calculate the cost of the male circumcision program per year and the net cost per HIV infection averted.

These inputs have been listed in the order in which they are found in the main menu page of the model.

3.1 Menu

Begin the Impacts section with the menu page. As with the Cost section, the menu serves as a directory for navigation through the program. Below is a snapshot of what the menu for the Impacts section looks like:

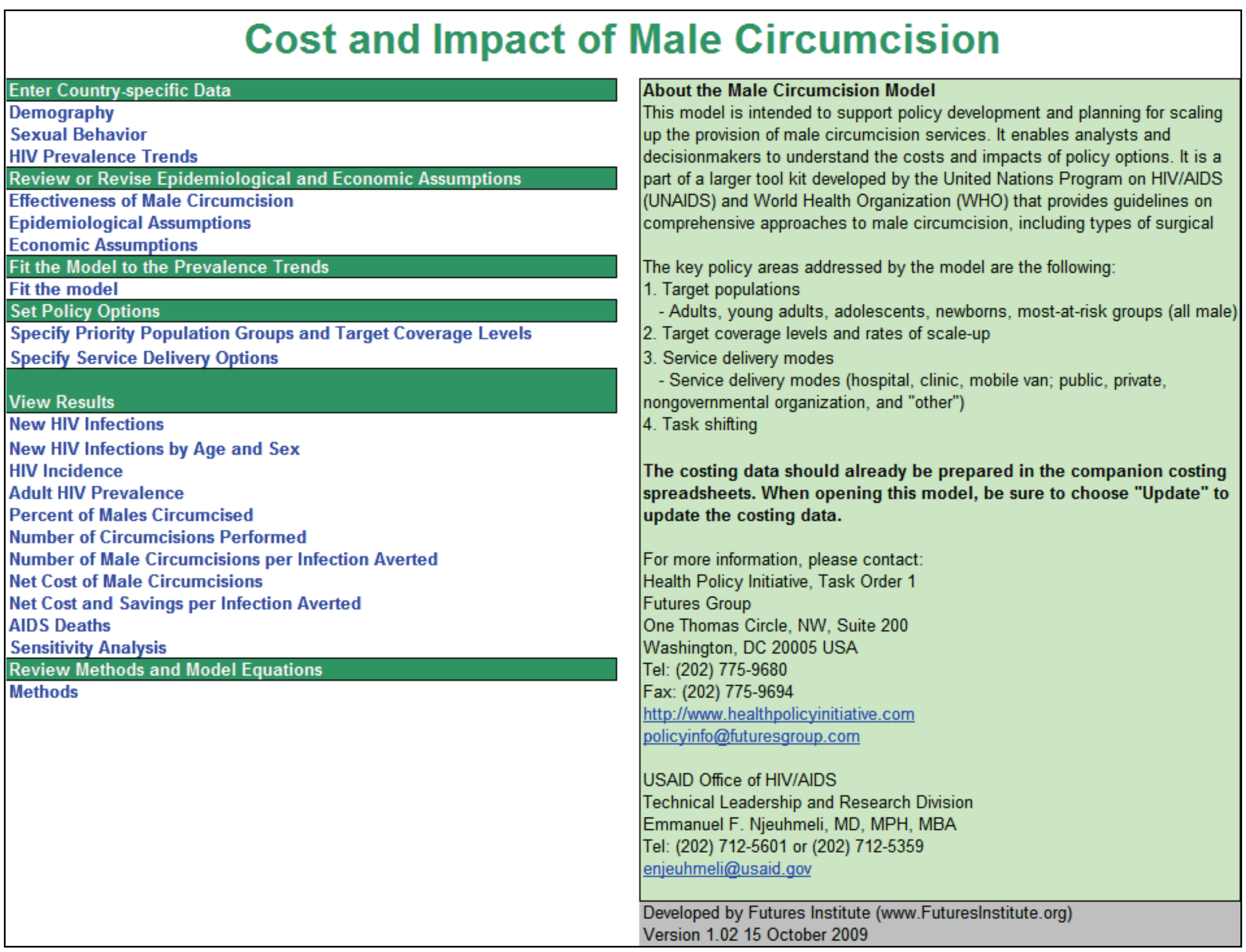

3.2 Demography

From the menu page, click on “Demography” to see a worksheet like the following:

Demographic Data — Return to Menu

Indicator	Value in 2008	Notes
Adult population (15-49)	1,077,908	
Adult population growth rate	2.42%	
Upper age limit of young age group	29	
Male pop aged 15-29 as proportion of Males 15-49	0.6009	
Male pop aged 15 as proportion of Males 15-49	0.0426	
Male pop aged 49 as proportion of Males 15-49	0.0123	
Female pop aged 15-29 as proportion of Females 15-49	0.6070	
Male pop aged 15 as proportion of Males 15-29	0.0708	
Male crude birth rate (per 1000 population aged 15-49)	24.1384	
Crude death rate 15-29	0.35%	
Crude death rate 30-49	1.45%	
Proportion surviving to age 15	0.92	
Proportion of adult population female	0.493	
Current prevalence of male circumcision	10.2%	

**Adjusting the Population Projection**		
Estimated adult population in 2008	1,077,363	<--This is the current estimate of the adult population in 2008.
Adjustment factor for 1961 population	0.92	<--Change this number to adjust the starting population
Crude birth rate in 1945 (per 1000 population aged 15-49)	103.22	

1. Enter in the data for each indicator from the country data.
2. Notice the lower limit for the age groups is 15 years old, and the upper limit is 49 years old. However, the adult population will be divided into two age groups, younger and older, by choosing the upper limit of the young age group. For example, if “34” is chosen, then your ages will be 15–34 for the younger age group and 35–49 for the older age group. The age groups designated here will be used throughout the program; the age groups change not only for the “Crude death rate” labels in the “Demographic” screen but also in the “Sexual Behavior” and “Fit the Model” screens to accommodate the designations. Thus, it is important to consider right from the outset the definition of “younger” and “older” age groups.
3. The “Estimated adult population in 2007” may not match the “Adult population (15–49)” in 2007 that was entered from country data, because the MC Model begins its calculations in 1961 and projects from there (so that there is a good estimate of 15 year olds before the HIV epidemic begins). Therefore, to make the model more accurate, manipulate the “Adjustment factor for 1961 population” up or down to make the “Estimated adult population in 2007” as close as possible to the “Adult population (15–49)” in 2007.
4. Further adjust the model’s population projection by entering in the crude birth rate in 1945. Because the model looks at ages 15–49, the crude birth rate in 1945 is a good indicator of the 15-year-old population in 1961.

3.3 Sexual Behavior

From the menu page, click on “Sexual Behavior” to see a worksheet similar to the one below:

Sexual Contacts | **Return to Menu**

Proportion of Sexual Contacts by Age of Partner

	Females 15-29	Females 30-49	Total Females
**Males 15-29**	0.21	0.22	0.43
**Males 30-49**	0.28	0.29	0.57
**Total Males**	0.49	0.51	1.00 <--------- The sum should be 1.00

**Risk Compensation**	
**Percent of behavior change that is reversed when circumcised**	0%
**What population is affected by risk compensation?**	All circumcised men

1. Enter in the best estimate for the proportion of sexual contacts by age of partner. There are four boxes to fill in, and their sum must equal 1.00. Information on the proportion of sexual contacts by age of partner can be derived from information on the proportion of the population married, proportion of the population sexually active, and Demographic and Health Survey or other survey data on age difference in married partners.
2. If it appears that there is a reversion to sexual behaviors that existed prior to the HIV epidemic, enter the estimate for risk compensation. Below “Percent of behavior change that is reversed when circumcised,” also designate to which group the percentage estimate applies. Select one of the categories from the drop-down menu that appears when clicking on the box next to “What population is affected by risk compensation?” Choose either all circumcised men, newly circumcised men, or all men.

3.4 HIV Prevalence Trends

Click on “HIV Prevalence Trends” on the main menu to display a screen like the one below:

HIV Prevalence Data

Return to Menu

Year	Adult HIV Prevalence %
1985	0.7%
1986	1.0%
1987	1.5%
1988	2.2%
1989	3.3%
1990	4.7%
1991	6.7%
1992	9.2%
1993	12.1%
1994	15.3%
1995	18.3%
1996	21.0%
1997	23.1%
1998	24.5%
1999	25.4%
2000	25.9%
2001	25.9%
2002	25.7%
2003	25.3%
2004	24.7%
2005	24.1%
2006	23.4%
2007	22.7%

Spectrum projection with no ART

This input usually comes from EPP or Spectrum. It is used to fit the model to the estimated prevalence trend.

**Population Survey**	
Year of population survey	2004
**HIV Prevalence from Population Survey**	
Male 15-29	10.7%
Male 30-49	34.4%
Female 15-29	25.1%
Female 30-49	35.8%
**HIV PREVALENCE**	
Male 15-29	10.7%
Male 30-49	34.2%
Female 15-29	24.9%
Female 30-49	35.5%
**POPULATION**	
Male 15-29	328,308
Male 30-49	218,013
Female 15-29	322,697
Female 30-49	208,891
**WEIGHTED AVERAGE**	24.50%
New Weighted average	24.69%

1. Enter in adult HIV prevalence from 1985 to 2007. These data may come from an Estimation and Projection Package (EPP) worksheet, a Spectrum file, or other national source. Use the adult HIV prevalence rates that result when coverage of ART is excluded, as including ART increases the overall HIV prevalence rate and hence (artificially) new infections.
2. Choose a recent year for which survey data are available.
3. Enter in the HIV prevalence rate from the survey for males and females in the specified age groups.

3.5 Effectiveness of Male Circumcision

At the main menu, click on "Effectiveness of Male Circumcision" to access the following worksheet:

Effectiveness of male circumcision — Return to Menu

**Reduction in annual probability of infection when circumcised**		
**Female to male**		
- General population	60%	Sources: 1, 2, 3
- Most-at-risk population	69%	Source: 4
- Other population 1	69%	
- Other population 2	69%	
- Other population 3	69%	
**Male to female**	0%	

Sources:
1. Auvert B, Taljaard D, Lagarde E, Sobngwi-Tambekou J, Sitta R, et al. (2005) Randomized, controlled intervention trial of male circumcision for reduction of HIV infection risk: The ANRS 1265 trial. PLoS Med 2(11): e298.
Protective effect = 60% (32%-76%)
2. Gray RH, Kigozi G, Serwadda D, Makumbi F, Watya S, Nalugoda N et al. Male circumcision for HIV prevention n men in Rakai, Uganda: a randomized trial. Lancet 2007; 369: 767-66.
Protective effect = 55% (22%-75%)
3. Bailey RC, Moses S, Parker CB, Agot K, Maclean I, Krieger JN, et al. Male circumcision for HIV prevention in young men in Kisumu, Kenya: a randomized controlled trial. Lancet 2007; 369: 643-56.
Protective effect = 60% (32%-77%)
4. Weiss HA, Quigley MA, Hayes R. Male circumcision and risk of HIV infection in sub-Saharan Africa: a systematic review and meta-analysis. AIDS 2000, 14:2361-2370.
Protective effect among most-at-risk men = 71% (59%-80%)

**Calculated force of infection for those not circumcised (For information only, no inputs required)**	
Male 15-29	0.13
Female 15-29	2.05
Male 30-49	0.92
Female 30-49	2.27

1. Review the default values for the general population, the most-at-risk population, and other populations (of choice) for the reduction in probability of becoming infected by a circumcised male from sex with an infected female. The default data for the general population and most-at-risk population come from the literature and will probably not change unless there is country-specific research that is contradictory. The default value for the "Other" groups can be changed to a more accurate estimate of the reduction; note that when the "Other" populations are specified on the "Specify Priority Population" worksheet (described below), the titles carry over to the "Effectiveness of Male Circumcision" worksheet.

3.6 Epidemiological Assumptions

Click on “Epidemiological Assumptions” on the main menu to find the following worksheet:

Epidemiological Assumptions — Return to Menu

Probability of transmission from mother-to-child	0.32
Fertility reduction due to HIV	0.80

Parameters for survival function

alpha	3.5
beta	12.2

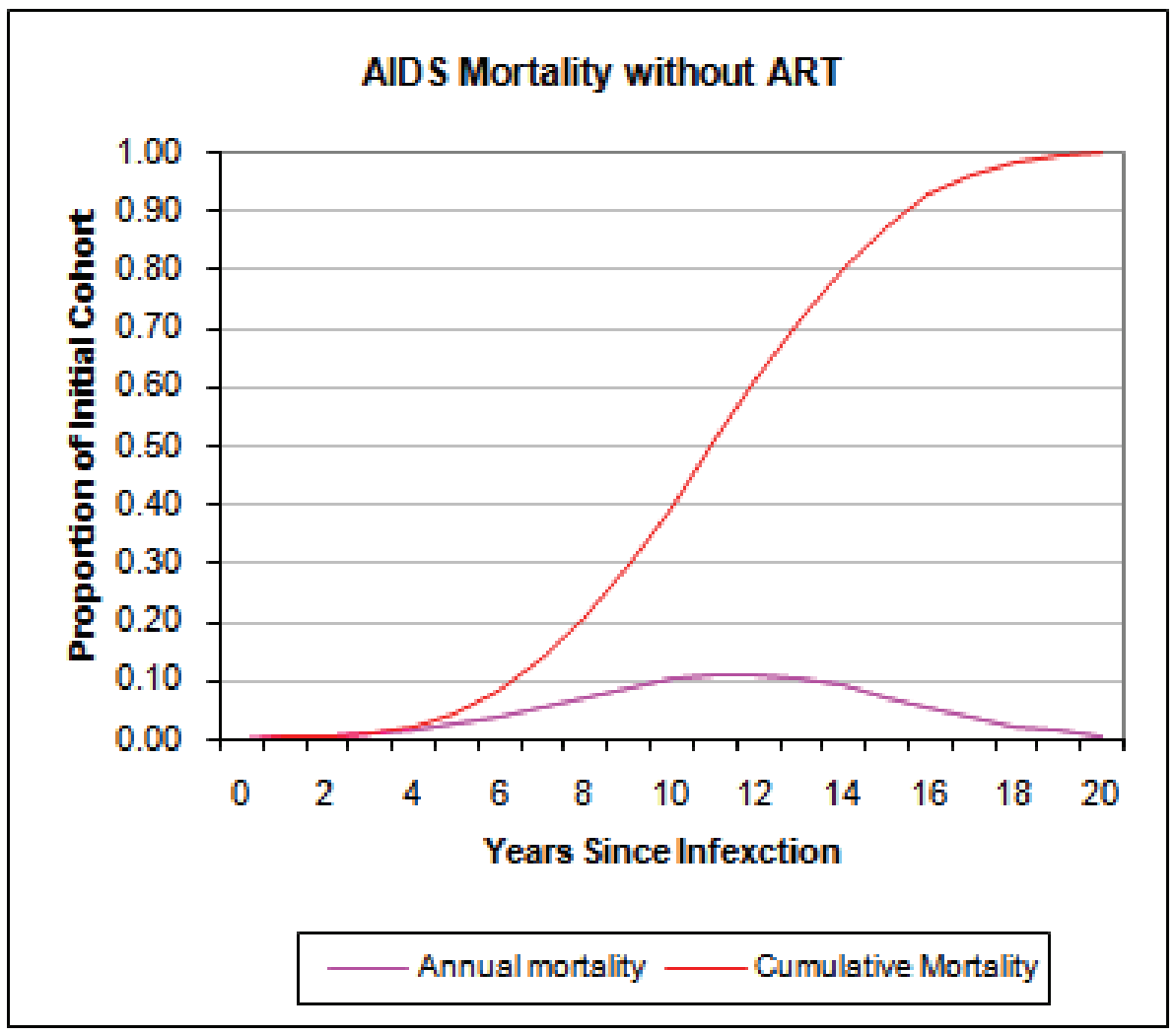

Years Since Infection	Proportion Dying from AIDS	Cumulative Proportion Dead
0	0.00	0.00
1	0.00	0.00
2	0.00	0.00
3	0.01	0.01
4	0.01	0.02
5	0.02	0.04
6	0.04	0.08
7	0.05	0.13
8	0.07	0.20
9	0.09	0.29
10	0.10	0.39
11	0.11	0.50
12	0.11	0.61
13	0.10	0.71
14	0.09	0.80
15	0.07	0.87
16	0.05	0.92
17	0.03	0.96
18	0.02	0.98
19	0.01	0.99
20	0.01	1.00

Time step for model calculations	0.1

<--- This should remain at 0.1 for normal use.

1. Review the default values for the probability of transmission from mother to child and fertility reduction due to HIV. These estimates are based on literature but may be changed if more accurate data for the country is available.
2. The alpha and beta parameters for the survival function are set to 3.5 and 12.2 and should remain so.
3. The time step for model calculation is set at .1 and should remain so for normal use.

3.7 Economic Assumptions

From the menu, click on the label "Economic Assumptions" to access the following page:

Economic Assumptions — Return to Menu

Discount rate	3%	Default value
Discounted lifetime cost of ART	$7,400	

1. Review the discount rate, and change it as necessary.
2. Review the discounted lifetime cost of ART, and change it as necessary.

3.8 Fit the Model

Click on "Fit the Model" on the menu page to see the following spreadsheet:

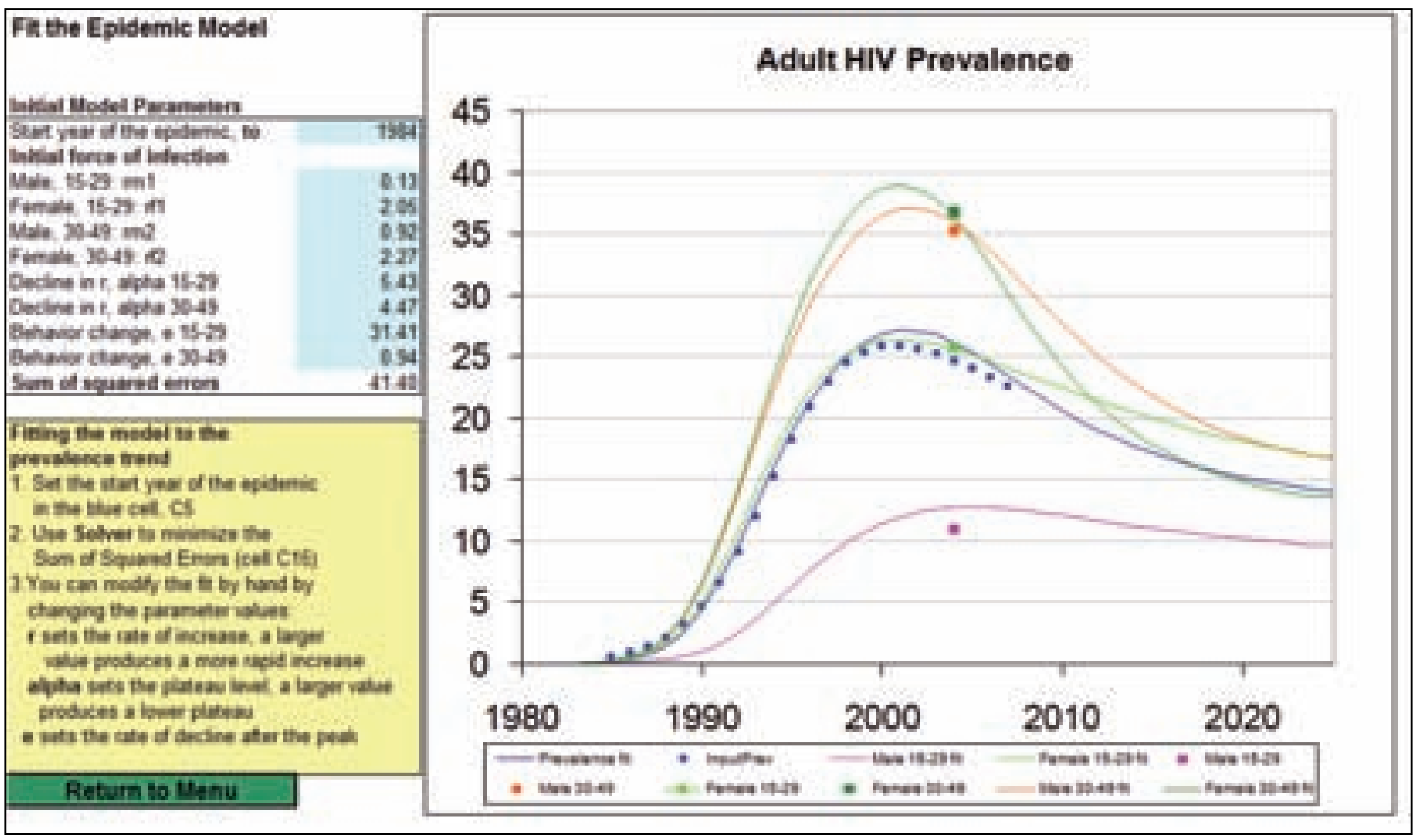

1. Set the start year of the epidemic. There will be a blue dotted curve representing the adult HIV prevalence entered previously.
2. Then select "Solver" from the Excel menu to fit curves for the age/gender groups.
3. The curves appear in different colors for each age/gender group, and a point(s) in the matching color represents the national survey data that were entered for each specific year(s).
4. If the curves do not match up to the data points very well, try to manually fit the curves by changing the model parameters: force of infection, the plateau level for the epidemic, or behavior change. Look for a close fit for all curves and a low sum of squared errors.

3.9 Specify Priority Population Groups and Target Coverage Levels

Click on the "Priority Population and Coverage Circumcision" tab to be directed to the following worksheet:

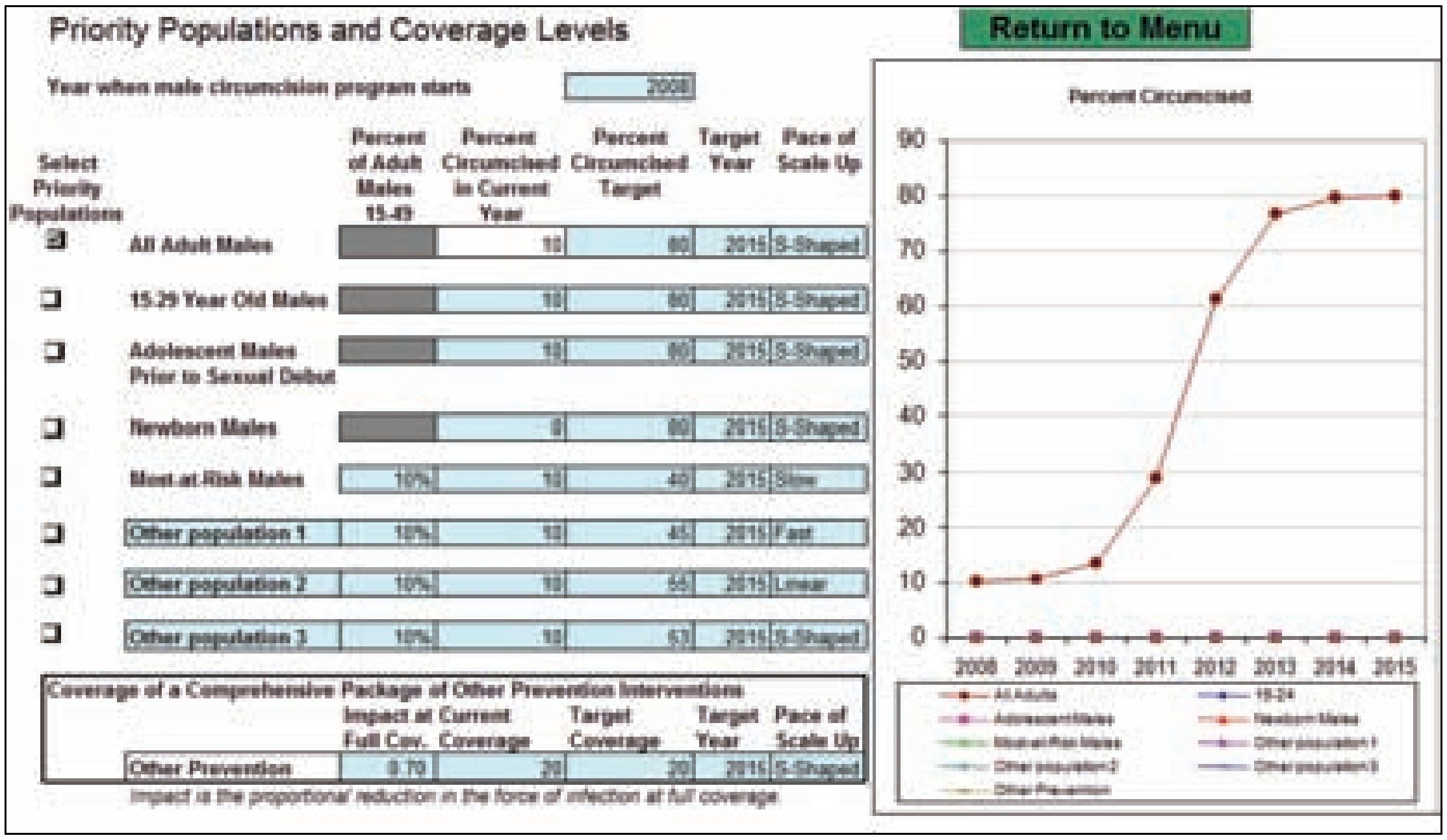

This is the worksheet for use in determining what the program will look like.

1. Select the year in which the male circumcision program will begin.
2. Choose the priority population or populations to be targeted. If choosing "All Adult Males," do not select "15–24 Year Old Males" or "Most-at-Risk Males" as well, as they are included in the category "All Adult Males." A population(s) not on the list can be chosen by typing a label(s) into the "Other population" box(es).
3. If choosing "Most-at-Risk Males" or designating another population as "Other," specify the percent of the adult male population (ages 15–49) made up of most-at-risk males or that "Other" group.
4. For each priority population selected, verify the initial percent circumcised in the current year.
5. For that priority population, then also select the percent circumcised target.
6. Choose the target year for program completion.
7. Choose the pace of scale-up for the program for that priority population: slow, fast, linear, or s-shaped by selecting from the drop-down box.
8. Finally, designate the coverage of a comprehensive package of other prevention interventions. Specify the impact at full coverage, the current coverage, the target coverage, the target year, and the pace of scale-up. This allows for consideration of a scenario where other prevention interventions are scaled up.
9. As the variables for the program are chosen, the graph on the right showing percent circumcised will change to reflect the choices.

3.10 Specify Service Delivery Options

This worksheet helps to determine how the program is delivered. From the main menu, select "Specify Service Delivery Options" to see the following worksheet:

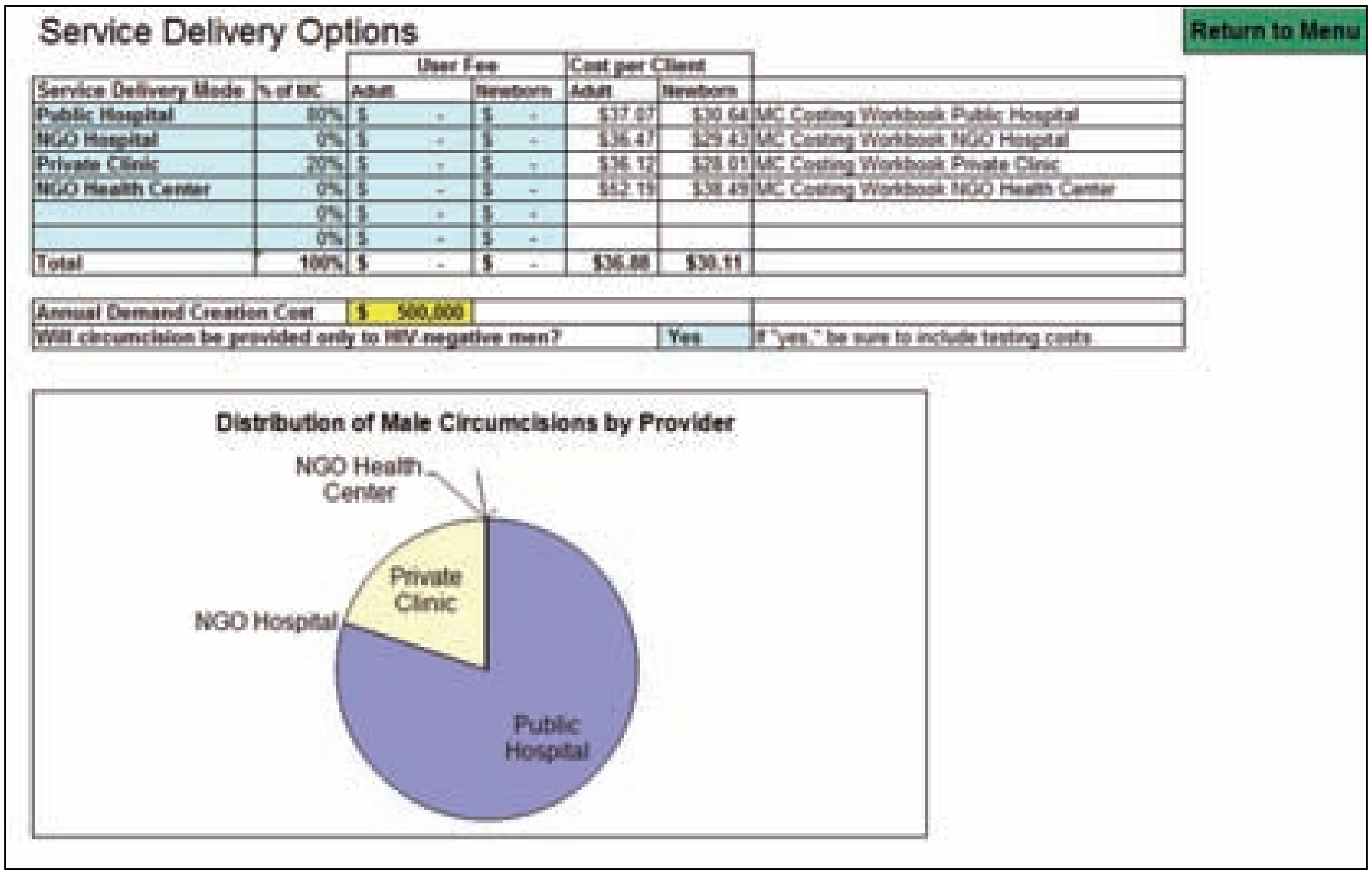

Service Delivery Options

Return to Menu

Service Delivery Mode	% of MC	User Fee Adult	User Fee Newborn	Cost per Client Adult	Cost per Client Newborn	
Public Hospital	80%	$ -	$ -	$37.07	$30.64	MC Costing Workbook Public Hospital
NGO Hospital	0%	$ -	$ -	$36.47	$29.43	MC Costing Workbook NGO Hospital
Private Clinic	20%	$ -	$ -	$36.12	$28.01	MC Costing Workbook Private Clinic
NGO Health Center	0%	$ -	$ -	$52.19	$38.49	MC Costing Workbook NGO Health Center
	0%	$ -	$ -			
	0%	$ -	$ -			
Total	100%	$ -	$ -	$36.88	$30.11	

Annual Demand Creation Cost	$ 500,000	
Will circumcision be provided only to HIV-negative men?	Yes	If "yes," be sure to include testing costs

1. Choose the various service delivery modes through which the program will be delivered. The service delivery modes should dovetail with the costing worksheets that were prepared by provider type. Under "Service Delivery Mode" above, six slots are shown, which allow the flexibility to enter up to six delivery modes.
2. Enter the percentage of total male circumcisions performed through each service delivery mode.
3. Enter a user fee for each delivery mode if there is one.
4. Review the costs for an adult or newborn circumcision by service delivery mode, which are calculated in the costing model.
5. Review the Annual Demand Creation Cost, which is calculated in the "National IEC Campaign" worksheet in the costing model.
6. Choose if circumcisions will only be provided to HIV-negative men. If "yes," make sure to include testing costs in the costing section.

The inputs are now complete, and the projection output displays can be viewed.

3.11 View Results

The results of the projection can be reviewed by clicking in the main menu on one of the possible outputs listed under "View Results":

1. New HIV Infections
2. New HIV Infections by Age and Sex
3. HIV Incidence
4. Adult HIV Prevalence
5. Percent of Males Circumcised
6. Number of Circumcisions Performed
7. Number of Male Circumcisions per Infection Averted
8. Net Cost of Male Circumcisions
9. Net Cost and Saving per Infection Averted
10. AIDS Deaths

In addition to these output variables, a Sensitivity Analysis can be performed, which will allow for varying assumptions in the model one at a time and then observing the resulting changes in infections averted, number of circumcisions per infection averted, net cost per infection averted, and cost savings per infection averted.

To conduct a sensitivity analysis, click on the "Sensitivity Analysis" label from the "View Results" section on the menu page. The spreadsheet will look like the following:

Sensitivity Analysis | **Return to Menu**

Perform Sensitivity Analysis

	Parameter Values	Results (2009-2025) Infections Averted	Number of Circumcisions per Infection Averted	Net Cost per Infection Averted	Cost Savings per Infection Averted
Base Case		51,518	7.3	$689	$10,569
Effectiveness	30%	25,059	14.7	$1,410	$9,848
	60%	51,518	7.3	$689	$10,569
	75%	65,218	5.8	$545	$10,713
Reduction in M->F Transmission	***0%***	51,518	7.3	$689	$10,569
	30%	67,444	5.7	$508	$10,750
Discount Rate	***3%***	51,518	7.3	$689	$10,569
	5%	51,518	7.3	$741	$10,517
	7%	51,518	7.3	$798	$10,460
Lifetime Cost of ART	$8,000	51,518	7.3	$689	$7,311
	$11,000	51,518	7.3	$689	$10,311
	$14,000	51,518	7.3	$689	$13,311
	Minumum	25,059	5.7	$508	$7,311
	Base Case	51,518	7.3	$689	$10,569
	Maximum	67,444	14.7	$1,410	$13,311

Default parameter values used in the sensitivity analysis are listed in light blue. They can be changed or kept as they are. For review, the default assumptions in the model are the following:

- Effectiveness of 60 percent
- Reduction in M->F Transmission of 0 percent
- Risk compensation of 0 percent
- Discount rate of 3 percent
- Lifetime cost of ART of US$7,400

After completing the entries, click on the "Perform Sensitivity Analysis" button to review the output, including infections averted, the number of circumcisions per infection averted, the net cost per infection

averted, and the cost saving per infection averted for each of the five parameters and the different parameter values. In yellow, the minimum effect or the maximum effect that can be achieved with a change in one of the parameter values is shown.

3.12 Methodology

To review the detailed methodology behind the MC Model, go to the main menu and click on “Methods.”

Health Policy Initiative, Task Order 1
Futures Group
One Thomas Circle, NW, Suite 200
Washington, DC 20005 USA
Tel: (202) 775-9680
Fax: (202) 775-9694
Email: policyinfo@futuresgroup.com
http://ghiqc.usaid.gov
http://www.healthpolicyinitiative.com
