# Supplementary material for: Cost comparison of the ShangRing device versus Mogen clamp for early infant male circumcision in Sub-Saharan Africa
Source: PLoS One. 2026 Oct 13;21(10):e0360356. doi: 10.1371/journal.pone.0360356 (PMC13660899; doi:10.1371/journal.pone.0360356)
Supplement: Supplementary file 2 [file pone.0360356.s002.pdf]

Goals Manual

A Model for Estimating the Effects of Interventions and Resource Allocation on HIV Infections and Deaths

Spectrum Versions 4.43 and Higher

Futures Institute

August 2011

Table of Contents

Contents

Table of Contents 1

I. Introduction 3

What is Goals? 3

Why Use the Goals Model? 4

Steps in Using the Goals Model 5

II. Quick Start Tutorial 7

Basic Steps in Using Spectrum 7

Step 1. Install Spectrum 7

Step 2. Select the language 7

Step 3. Start Spectrum 7

Step 4. Create a population projection 8

Step 5. Display the Goals menus 9

Step 6. Configure 9

Step 7. Epidemiology 11

Step 8. Specify behaviors 13

Step 9. Specify intervention coverage for behavioral interventions 17

Step 10. Biomedical interventions 17

Step 11. Vaccines 18

Step 12. Advanced options 18

Step 13. Results 19

Step 14. Additional Results 21

Step 15. Save the projection 21

Step 16. Creating alternate projections 22

Step 17. Uncertainty analysis 22

III. Fitting the Epidemic 25

IV. Description of Methods in the Goals Model in Spectrum 27

Introduction 27

Structure 27

Sexual Transmission of HIV 28
Transmission among Injecting Drug Users 31
Impact of Biomedical Interventions 31
Impact of Behavior Change Interventions 32
Progression of HIV-Infected Population 34
Allocation of New ART Patients by CD4 Count 35
Demographic Processes 35
Parameter Values 36
References 40

I. Introduction

What is Goals?

The **Goals** model is intended to support strategic planning at the national level by providing a tool to link program goals and funding. The model can help answer several key questions:

- How much funding is required to achieve the goals of the strategic plan?
- What goals can be achieved with the available resources?
- What is the effect of alternate patterns of resource allocation on the achievement of program goals?

The **Goals** model does not provide all the answers. It is intended to assist planners in understanding the effects of funding levels and allocation patterns on program impact. The model can help planners understand how funding levels and patterns can lead to reductions in HIV incidence and prevalence and improved coverage of treatment, care and support programs. It does not, however, calculate the "optimum" allocation pattern or recommend a specific allocation of resources between prevention, care and mitigation.

Goals is intended for use by national programs to explore the effects of different funding levels and patterns on national goals. It is generally implemented by a multi-disciplinary team composed of participants with various areas of expertise (demography, epidemiology, health finance, planning) representing different aspects of society (government, civil society, private sector, donors). A technical team works together to implement the model for the first time. Then the model is used in interactive workshops with planners and stakeholders to explore the effects of different program configurations on the provision of care and support and the prevention of new HIV infections. Through this interaction participants gain a better understanding of the dynamics of funding and impact. This prepares them to develop realistic budgets and goals that reflect their priorities. A typical application may take two weeks to set up the model, which can then be used with occasional updating to support annual planning exercises and ad hoc studies.

Designing and implementing national program HIV/AIDS is complex and important. Prevention, treatment, and palliative care costs are a significant component of the health budget. In most national strategic plans, although the activities to be undertaken are clearly outlined, these activities are not tied to specific prevalence goals the countries want to attain. By projecting estimates of future program needs, **Goals** can help planners respond to changes in intervention, care, and drug therapy funding. It should be noted that when trying to provide high-quality HIV/AIDS services, political, legal, and ethical considerations sometimes take priority over economic issues in determining the most cost-effective resource allocation pattern.

Designing and incorporating an HIV/AIDS program into strategic plan goals is a complicated process requiring skilled and dedicated personnel, appropriate technologies, legislation, infrastructure, and adequate funds. Note that, although **Goala** can be used to show how the distribution of funds will affect HIV/AIDS prevalence and coverage, it does not imply that program goals can be achieved merely by allocating the necessary funds.

Why Use the Goals Model?

The **Goals** model may be used for several purposes. Millions of dollars are spent annually to prevent HIV infection without a thorough understanding of the most effective way to allocate these funds. Therefore, one reason to use the **Goals** model is to estimate the impact of budget decisions on the achievement of HIV/AIDS program goals. By anticipating the consequences and trade-offs of allocation options, **Goals** can support the decision making process and may include the following activities:

- Estimating reductions in HIV prevalence
- Estimating increases in coverage of essential care and treatment services.
- Allocating resources between prevention and care programs.
- Setting priorities for high-risk populations.

By understanding how resources need to be spent to achieve proposed goals, these simulations can help planners choose goals that are attainable and useful. For example, information on the magnitude of the economic effects of STIs on the prevalence and incidence of HIV may move the allocation of resources away from other sectors and toward the health sector.

Another important use of the **Goals** model is the examination of alternative resource allocation strategies. Rather than simply project current expenditures into the future, the analyst can ask, “How much funding is required to achieve national coverage for the most cost-effective interventions? How much funding is required to provide palliative care to everyone who needs it? How many pregnant women can be treated with ARV to prevent maternal-to-child transmission? Would the allocation of additional expenditures to ART have a significant impact on HIV prevalence goals?"

The **Goals** model is not intended to replace the tools and techniques used in day-to-day operations. It is intended to support policy dialogue about the allocation of resources and its contribution to achieving the goals of the HIV/AIDS national strategic plan. It is designed to support discussion of long-term issues, such as reduction in HIV incidence and prevalence, the implications of initiating ART, alternative options in preventing mother-to-child transmission, and the funding requirements to meet national HIV and STI health goals.

Steps in Using the Goals Model

There are six major steps involved in using **Goals**:

1. Form a national team to implement the model. The model needs to be implemented by a national team that can be trained in the use of the model and can apply it to the national strategic plan. This team will generally receive some initial training in the use of the model and then extensive training as the model is set up and used. Ideally the model will be implemented by a multi-disciplinary team composed of participants with various areas of expertise (demography, epidemiology, health finance, planning) representing different aspects of society (government, civil society, private sector, donors).

2. Collect data on HIV/STI prevalence, sexual behavior, and the costs of prevention and care programs. The **Goals** model contains a large amount of information obtained from published studies on the cost and impact of prevention and care programs. This information can be used or replaced with locally available data. It also requires national data on the population size and distribution, adult HIV and STI prevalence and sexual behavior (e.g., condom use and number of partners).

3. Adapt the model to the national strategic plan. The model is designed to show the consequences of allocating funds to various prevention, care and treatment programs. To do this, the activities in the strategic plan need to be linked (or mapped) to the categories in the model. This may require adding some line items for activities that are in the plan but not in the model, or mapping the budget categories in the plan to those used in the model.

4. Enter data specific to **Goals**. Once the data described in step 2 are collected for the current year and decisions are made about funding options and adapting the model to the activities in the strategic plan, the data are entered into the **Goals** model.

5. Conduct resource allocation workshops. In most applications the model will be used in a workshop with decision makers. The workshop will be an interactive session where participants will try out different resource allocation strategies and observe the consequences. Participants may use the model to examine different types of issues, such as "Which prevention interventions are most cost-effective?" "How many people are receiving palliative care with the current allocation?" "How many people can be treated with ART with available funds? How would that change if drug prices were lower?" As various options are tested with the model the participants will gain a better understanding of the trade-offs involved and the amount of funding required to achieve the goals.

6. Follow-up on workshop outcomes. A variety of workshop outcomes are possible. Ideally the model is applied as part of the overall strategic planning process. In this case the model may continue to be used as goals are revised and funding plans are developed. The workshop may result in a new budget for the plan, or a commitment to raise additional funds to pay for essential programs. Reports and presentations may need to be prepared in order to disseminate the results to national decision makers, donors and program partners.

II. Quick Start Tutorial

This Quick Start tutorial describes how to:

- Create a demographic projection using data from the United Nations Population Division.
- Create an HIV/AIDS projection using **Goals**.
- Display various HIV/AIDS indicators such as the number of people infected, the number of new infections, AIDS cases, AIDS deaths, the number of people needing ART and the number of orphans.

Basic Steps in Using Spectrum

Step 1. Install Spectrum

The Spectrum program can be downloaded from the Futures Institute web site at www.futuresinstitute.org. Spectrum will run on any computer running Windows 95 or later Windows versions. It requires about 30MB of hard disk space.

To install Spectrum from a file downloaded from the internet, just double click on the file named “SpecInstall.exe”. This will start the installation program. Just follow the instructions on the screen to complete the installation.

If you have trouble installing Spectrum it may be that you do not have the appropriate permission to install programs on your computer. In that case you should contact your IT support office to do the installation for you.

Step 2. Select the language

The first time you run Spectrum all the displays will be in English. You can change to another language by selecting the Spectrum Menu Button (the rainbow at the top left of the screen), then “Options” and “Environment” from the Spectrum menu. Then select the language you want to use and click on the “Ok” button.

Step 3. Start Spectrum

Start the Spectrum program by selecting it from the “Start” menu on your computer. When the program starts you will see an opening screen giving you the option to “create a new projection” or “open an existing projection.”

Step 4. Create a population projection

When you create a new projection you will see the "Projection manager" dialogue box, shown below.

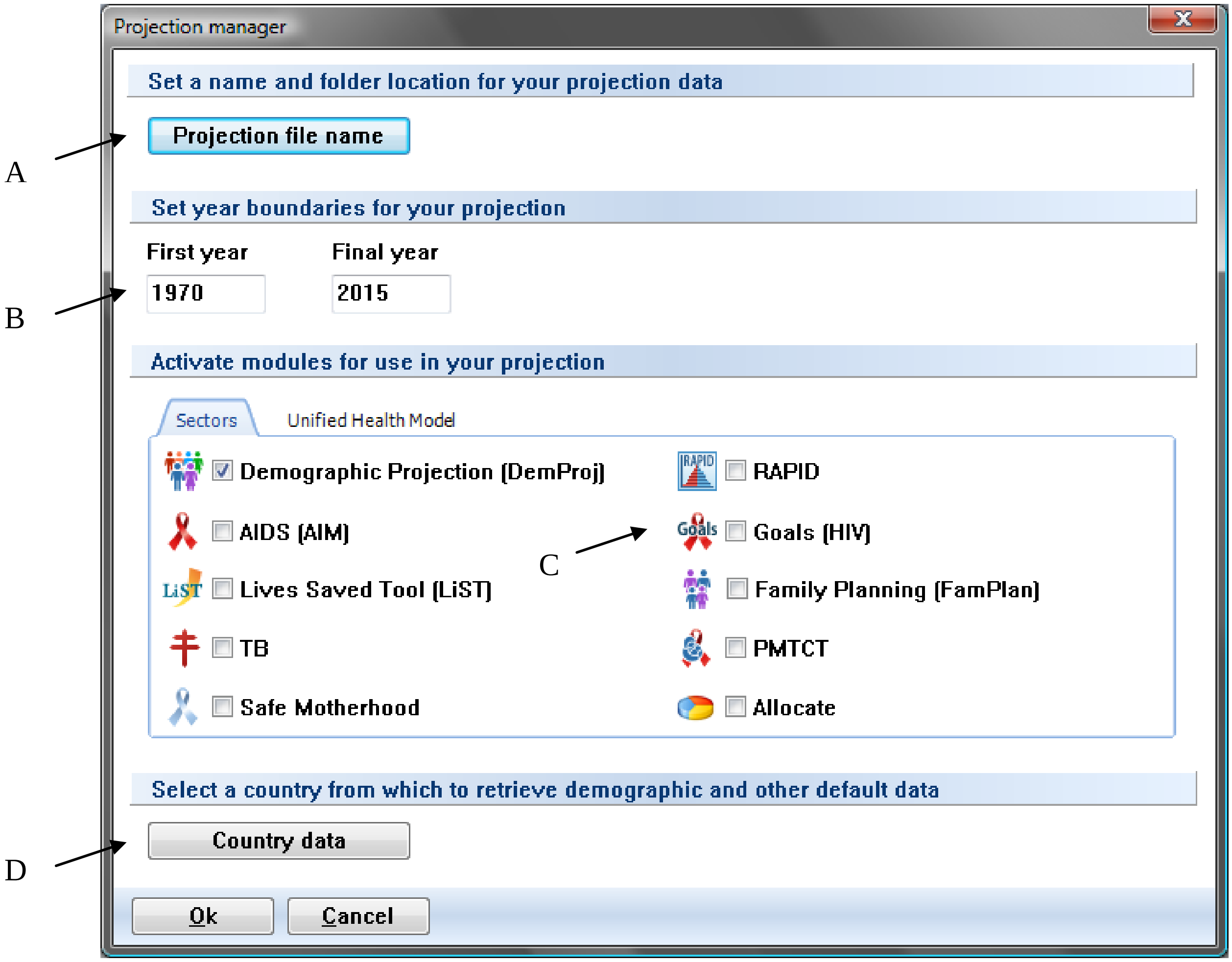

Follow these easy steps to complete the "Projection manager" screen:

A. Click the **Projection file name** button and enter a file name for the projection.

B. The **First year** and **Final year** will be set to 1970 and 2015 by default. You can change these to whatever you want, but the first year should be before the start of the AIDS epidemic since **Goal** needs to simulate the epidemic from the beginning.

C. Click the check box next to **Goals (HIV)** to add the **Goal** module to the projection.

D. Then click the **Country Data** button. A list of countries will appear. Once you select your country from the list Spectrum will automatically load all the demographic data you need for your projection.

In addition Spectrum will load data on HIV incidence, and number of people receiving ART, cotrimoxazole and PMTCT services based on data published by UNAIDS and WHO. It is important that users compare these inputs with their program data and make any revisions that might be necessary. In particular, the data provided on PMTCT services must be re-distributed by the actual regimens used in the country.

E. When you are done click the **OK** button.

Step 5. Display the Goals menus

Select **Modules** from the Spectrum menu and click the **Goal** icon to display the **Goal** menu as shown below.

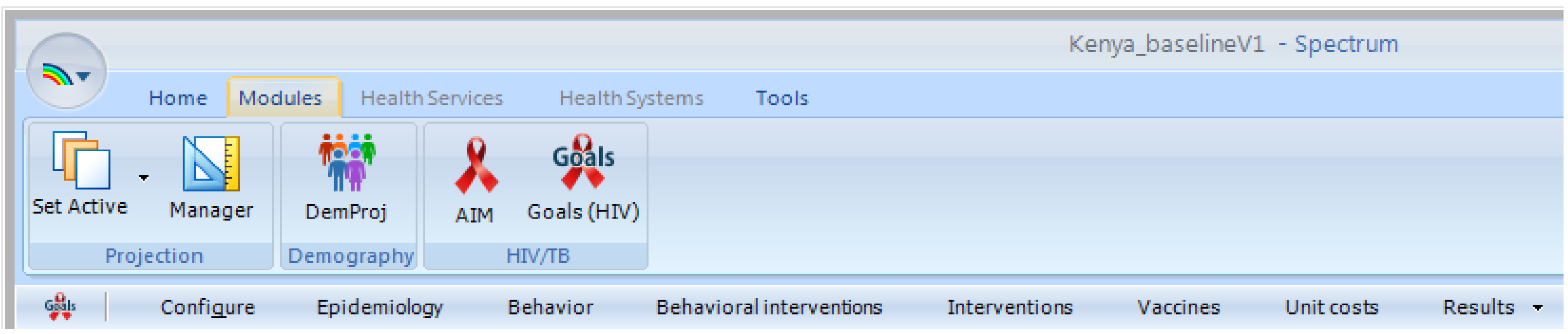

To produce the projection you just need to proceed through the first five menu items one-at-a-time: **Configure, Epidemiology, Behavior, Behavioral interventions and Interventions.** You can also look at **Vaccines** and **Unit costs** but those sections are not required. The following sections explain each of these items in detail.

All Spectrum editors have **duplicate** and **interpolate** functions to make it easier to enter all the data required. To use these features just select a range of data with your mouse and either click the **Duplicate** or **Interpolate** buttons or right click with the mouse and select **Duplicate** or **Interpolate** from the menu.

- **Duplicate** copies the data from the first cell of the range to all the other cells in the range. It is useful when you want values to remain constant over time.
- **Interpolate** calculates values for the cells between the first and last cell in the range by interpolation. Spectrum will perform a linear interpolation if you click the Interpolate button. It you right click you will see a menu allowing you to choose the interpolation method: linear, S-shaped, exponential, and front loaded.

Step 6. Configure

Click on the **Configure** menu item to see the program data editor. It will look like this:

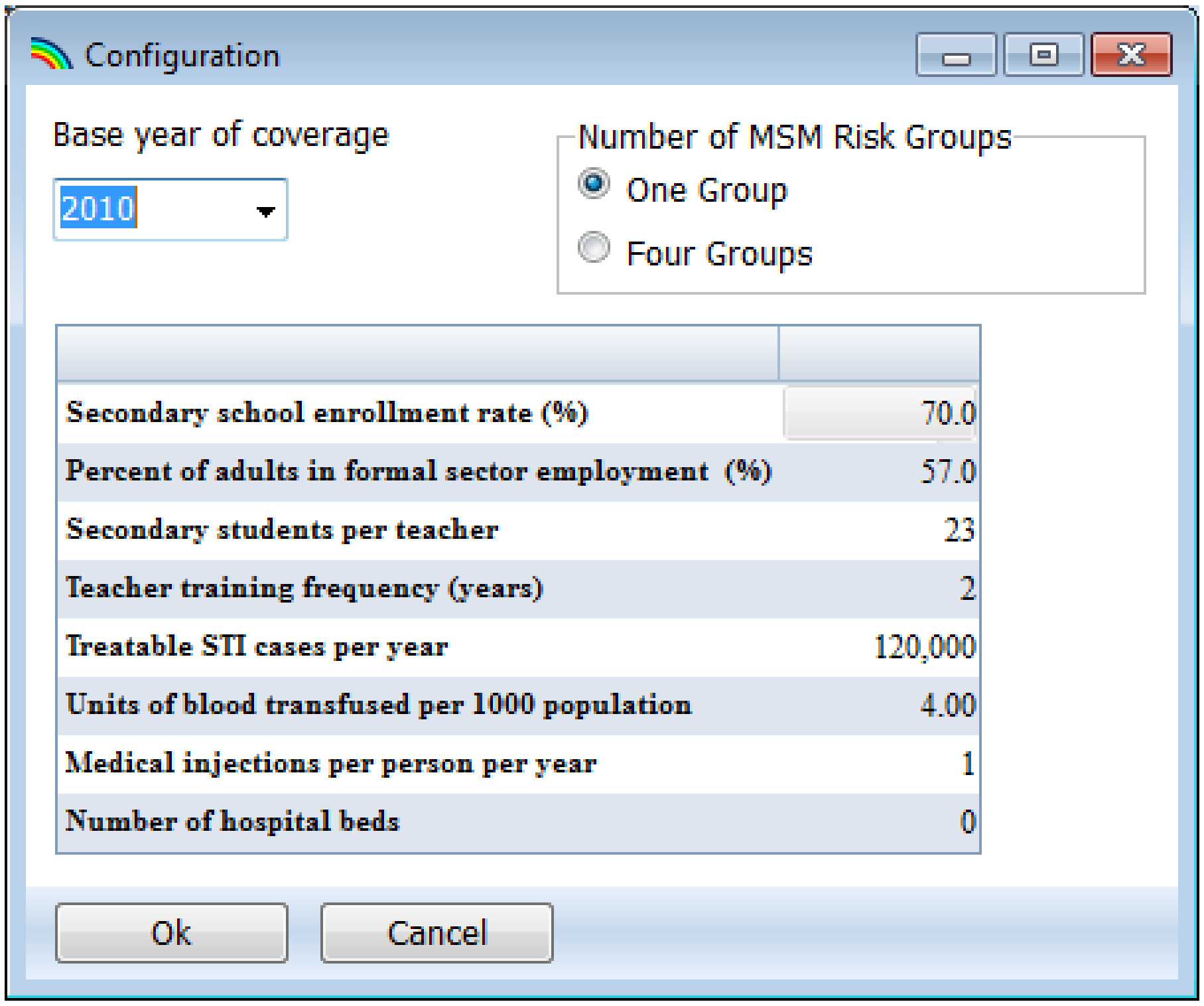

The entries are shown below. You only need to provide values for those inputs that will be used. For example, the 'Base year of coverage', the 'Secondary school enrollment rate' and the 'Percent of adults in formal sector employment' are all required to estimate the impact of prevention interventions. The other entries are required only for cost calculations.

- **Base year of coverage**: You directly enter key behaviors (condom use, number of partners, age at first sex, needle sharing) for all years up to and including the 'Base year of coverage'. These behaviors will be calculated for all years after the 'Base year of coverage' based on intervention coverage and impact. Thus, you should usually set this to be the current year or the last year for which you have coverage data for behavioral interventions (condom promotion, outreach, VCT, etc.)
- **Number of MSM risk groups:** For most applications this should be set to 'One group'. However, if you have enough information you can divide the MSM group into four activity groups based on the frequency of partner change: Low, Medium, High and MSM/IDU.
- **Secondary school enrollment.** The percentage of children of secondary school age enrolled in secondary school. This is used to determine the impact of population eligible for school-based AIDS education.
- **Percent of adults in formal sector employment.** The percentage of all adults 15-49 who are employed in the formal sector. This is used to determine the population potentially reachable through work place prevention programs.

- **Secondary students per teacher.** The number of secondary students per teacher. This is used to determine the number of students that can be reached by one teacher trained in HIV education.
- **Teacher training frequency (years).** The number of years between refresher training courses for secondary school teachers.
- **Treatable STI cases per year.** The number of cases of treatable STIs each year. This is used to determine the costs of STI treatment programs.
- **Units of blood transfused per 1000 population.** The number of units of blood transfused each year for every 1000 people in the population. This is used to determine the costs of blood screening programs.
- **Medical injections per person per year.** The average number of medical injections per person per year. This is used to determine the costs of programs to supply disposable or autodestruct syringes.
- **Number of hospital beds.** The total number of hospital beds in the country. This is used to calculate the cost of universal precautions.

When you are finished entering data, click the **Ok** button.

Step 7. Epidemiology

Next select ‘Epidemiology’ from the Goals menu and you will see an editor like this one:

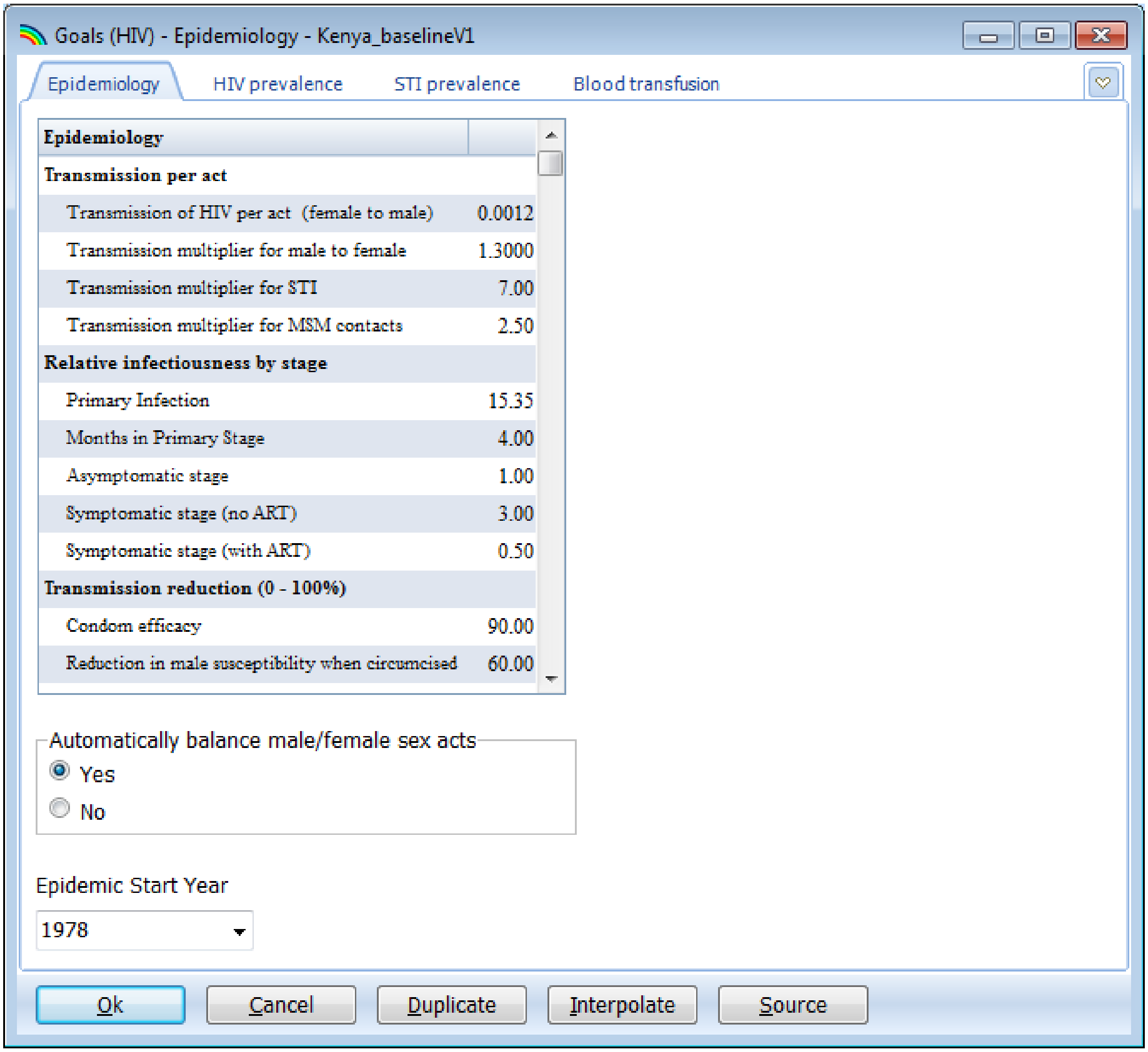

Under **Epidemiology** tab, you can review and set the key epidemiological parameter values. In many cases you can accept the default values provided. More detail on the source of these values is available in the Method section of this manual.

You should set the 'Epidemic Start Year' to the first year of the epidemic. This is the first year when prevalence is significantly larger than zero. Often this can be approximated by 2-3 years before first AIDS case report.

Under the **HIV Prevalence** tab you can enter any survey or surveillance data available on HIV prevalence in the different risk groups. For example if you have a national household survey that measured HIV prevalence you would be able to enter values for that year for 'Total Males', 'Total Females', and 'Adults'. You should only enter data for the years in which it was measured. You do not need to interpolate to fill in intervening years. Zeros in this editor are equivalent to

'No Data'. The data you enter here are used to compare against the model results in order to see how when the model fits the actual data. These data are not used in the model calculations.

Under the **STI Prevalence** tab you can enter information on the prevalence of STIs (sexually transmitted infections). Unlike the data on HIV prevalence, the data on STI prevalence are used in the calculations. Therefore you must enter data for all years and all risk groups. As described in the Methods section of this manual, the presence of STIs increases the probability of HIV transmission.

Under the **Blood transfusion** tab you can enter information on the number of people infected with HIV through contaminated blood transfusions. In most countries this accounts for only a small portion of new infections and, therefore, this section can be ignored. However, in a few countries this has been a key source of new infection and should be addressed here.

Step 8. Specify behaviors

Select **Behavior** from the Goals menu and you will see a tabbed editor like this:

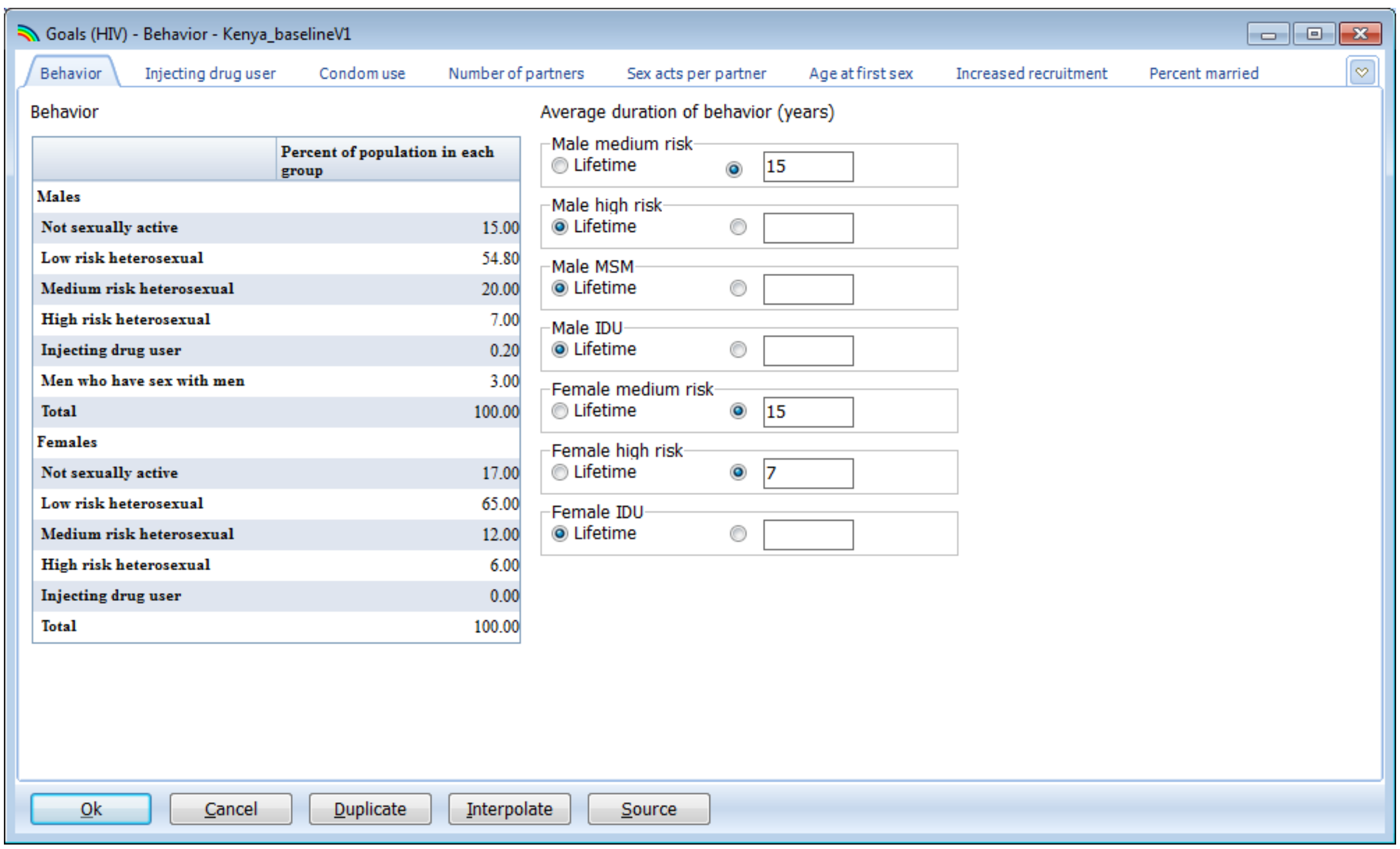

This editor is used to provide information on key behaviors. The main tabs are as follows:

- **Behavior**: Here you specify the distribution of the population by risk group. For both males and females the risk group proportions must sum to 100.0. Information on the sizes of risk groups may be available from national surveys, behavioral surveillance surveys, or other size estimation activities. This information is available for most countries since it is used in preparing national prevalence estimates. A summary is provided below.

Estimates of population sizes by region

(MSM and clients as % of male pop; FSW as % of female pop)

**Men having sex with men**		High risk
E. Asia	3 – 5%	<2.8%
S & SE Asia	6 – 12%	1.2-7.2%
E. Europe	6 – 15%	1-5.5%
Latin America	6 – 20%	0.8-4.8%

**Injecting drug users**	
E. Europe and C. Asia	0.06 – 5.21%
Asia and the Pacific	0.021 – 1.33%
M East, N. Africa and sub-Saharan Africa	0.05 – 2.07%
Latin America & Caribbean	0.29 – 1.15%

**Female sex workers**	
Sub-Saharan Africa	0.4 – 4.3%
Asia	0.2 – 2.6%
Russian Federation	0.1 – 1.5%
E. Europe	0.4 – 1.4%
W. Europe	0.1 – 1.4%
Latin America	0.2 – 7.4%

**Clients of sex workers**	
All	9 – 10%
Central Africa	13 – 15%
Eastern and southern Africa	10 – 11%
Asia	5 – 7%
Latin America	5 – 7%

Source: Sexually Transmitted Infections, 2006 Jun; 82 (suppl)3; Mathers B et al Lancet 2008, 372:1733-1745.

- Also in this editor you specify the average time spent in the risk group. For most risk groups the duration will be 'Lifetime'. However, for some risk groups, most notably 'Female high risk' duration in the risk group is usually only 2-10 years. In these cases you should click the radio button next to the box and enter the average number of years in the risk group in the box. The table below summarizes information on time in risk group from a number of studies.

Estimates of time in most-at-risk populations by region

Average duration of female sex work, by region

Region	Duration of behavior in years
Africa	5.5 (4 studies)
Asia/Oceania	2.9 (12 studies)
North America	10.2 to 11.0 (3 studies)
Europe	8.4 to 10.0 (10 studies)
Latin America	11.2 to 12.0 (6 studies)

Average duration of injecting drug use, by region

Region	Duration of behavior in years
Africa	5.6 (1 study)
Asia	8.7 (6 studies)
Oceania	17 (1 study)
Europe	13.9 (1 study)
North America	9.5 (1 study)
South America	21 or 19.6 (9 studies)

Source: Fazito E et al. Forthcoming

- **Injecting drug user**: In this editor you enter the percent of IDU that share needles and the force of infection for male and female IDU. The force of infection is used to determine the spread of HIV among IDU. The value can vary between 0 and 1. There is no direct way to determine this input. You need to try different values and examine the resulting IDU prevalence curves to determine the correct values. Typical values in countries with serious HIV epidemics among IDU start at 0.35 – 0.50 and usually decline with time as the highest risk population becomes infected first.
- **Condom use:** Here you enter the proportion of sex acts in each risk group in which condoms are used. You have two options for specifying condom use: 'Use interpolated values' requires you to enter values for each year of the analysis while 'Use logistics curve' allows you to create curves to describe the changes in condom use over time. The curves are displayed in the lower portion of the screen. The parameters of the curves are:
    - **Initial value.** Condom use at the beginning of the epidemic. Usually this is nearly zero.
    - **Final value.** The percentage of acts covered by condom use today.
    - **Growth rate.** The speed at which condom use increased from the initial value to the final value. The higher the value the more quickly the transition happens. Typical values would be between 0.5 – 0.8.

- **Growth location.** The parameter determines when most of the change took place. A low value, such as 0.1, will cause the growth to be recent, a higher value, such as 0.9, will cause it to start earlier.
- **Years to final value.** The number of years it takes to get from the initial value in the first year of the projection to the final value today. This determines when the curve stabilizes at the final value.

- **Number of partners.** The average number of different sexual partners per year for each risk group. This is generally 1 for low risk groups and higher for the medium and high risk populations.
- **Sex acts per partner.** The number of sex acts per partner per year. Note that this is not the total number of sex acts per year but the number of acts per partner. For low risk populations, with one partner, it will be the same as the number of sex acts per year. For groups with more than one partner the number of sex acts per year will be the average number of partners multiplied by the number of acts per partner. The number of sex acts and partners should be balanced within risk groups. Thus, within the medium and high risk populations the total number of sex acts for men and women should be the same. The total number of sex acts is the number of partners multiplied by the number of acts per partner multiplied by the proportion of the population in the risk group.
- **Age at first sex.** The average age at first sex. This is usually available from national household surveys such as Demographic and Health Surveys.
- **Increased recruitment.** The proportion of those becoming sexually active who enter each risk group is initially determined by the proportion of the total population in that risk group. However, since those in the highest risk groups will have higher mortality than those in the lowest risk groups, the proportion of the population in the high risk groups will decline over time. In some cases, when the high risk behavior is defined by an occupation, such as a sex worker, truck driver or miner, new people will be recruited to replace anyone who dies from AIDS. Thus the proportion in these risk groups may not decline over time due to replacement. In this editor you can specify the proportion of mortality that is replaced by more recruitment into the high risk population group.
- **Percent married.** In this editor you specify the proportion of those in each risk group that are married or in a long term union. For the low risk population this will be 1.0. For higher risk populations it will usually be less than one but not zero.

Step 9. Specify intervention coverage for behavioral interventions

The behavioral interventions editor looks like this:

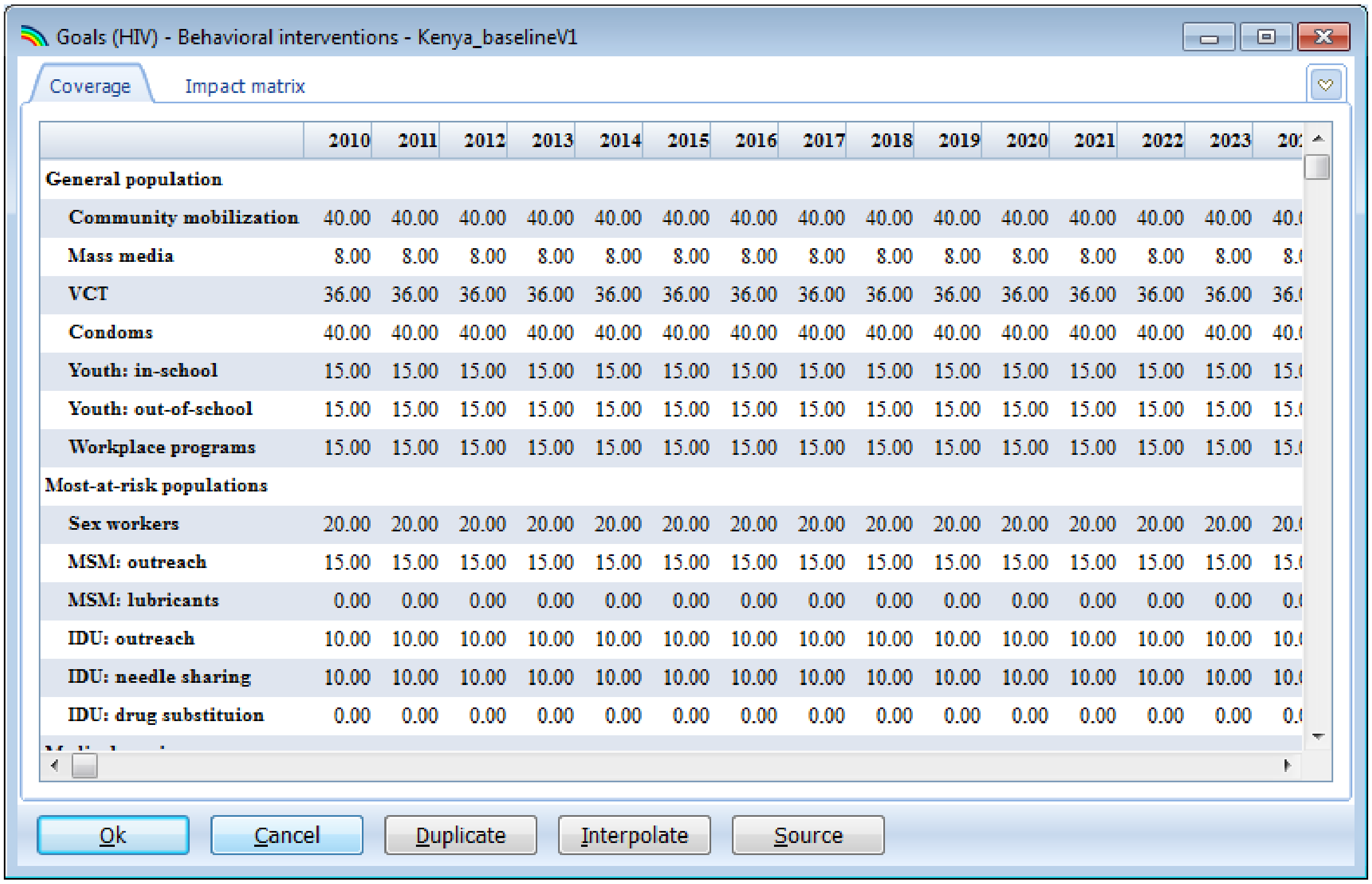

	2010	2011	2012	2013	2014	2015	2016	2017	2018	2019	2020	2021	2022	2023	20:
General population															
Community mobilization	40.00	40.00	40.00	40.00	40.00	40.00	40.00	40.00	40.00	40.00	40.00	40.00	40.00	40.00	40.
Mass media	8.00	8.00	8.00	8.00	8.00	8.00	8.00	8.00	8.00	8.00	8.00	8.00	8.00	8.00	8.
VCT	36.00	36.00	36.00	36.00	36.00	36.00	36.00	36.00	36.00	36.00	36.00	36.00	36.00	36.00	36.
Condoms	40.00	40.00	40.00	40.00	40.00	40.00	40.00	40.00	40.00	40.00	40.00	40.00	40.00	40.00	40.
Youth: in-school	15.00	15.00	15.00	15.00	15.00	15.00	15.00	15.00	15.00	15.00	15.00	15.00	15.00	15.00	15.
Youth: out-of-school	15.00	15.00	15.00	15.00	15.00	15.00	15.00	15.00	15.00	15.00	15.00	15.00	15.00	15.00	15.
Workplace programs	15.00	15.00	15.00	15.00	15.00	15.00	15.00	15.00	15.00	15.00	15.00	15.00	15.00	15.00	15.
Most-at-risk populations															
Sex workers	20.00	20.00	20.00	20.00	20.00	20.00	20.00	20.00	20.00	20.00	20.00	20.00	20.00	20.00	20.
MSM: outreach	15.00	15.00	15.00	15.00	15.00	15.00	15.00	15.00	15.00	15.00	15.00	15.00	15.00	15.00	15.
MSM: lubricants	0.00	0.00	0.00	0.00	0.00	0.00	0.00	0.00	0.00	0.00	0.00	0.00	0.00	0.00	0.
IDU: outreach	10.00	10.00	10.00	10.00	10.00	10.00	10.00	10.00	10.00	10.00	10.00	10.00	10.00	10.00	10.
IDU: needle sharing	10.00	10.00	10.00	10.00	10.00	10.00	10.00	10.00	10.00	10.00	10.00	10.00	10.00	10.00	10.
IDU: drug substituion	0.00	0.00	0.00	0.00	0.00	0.00	0.00	0.00	0.00	0.00	0.00	0.00	0.00	0.00	0.

In the 'Coverage' tab you specify the coverage of each intervention, where coverage is the percentage of the eligible population reached by the intervention. The first year of the editor is the year you specified in the 'Configuration' editor as the 'Base year of coverage'. The impact of these interventions on the key behaviors is determined by the increase in coverage from the first year. If coverage is constant then behaviors will not change from the base year.

The 'Impact Matrix' tab you can view and change any of the values in the impact matrix. It is described in the Methods section of this manual.

Step 10. Biomedical interventions

In the 'Biomedical interventions' editor you can specify future coverage of male circumcision, ART, PrEP and microbicides.

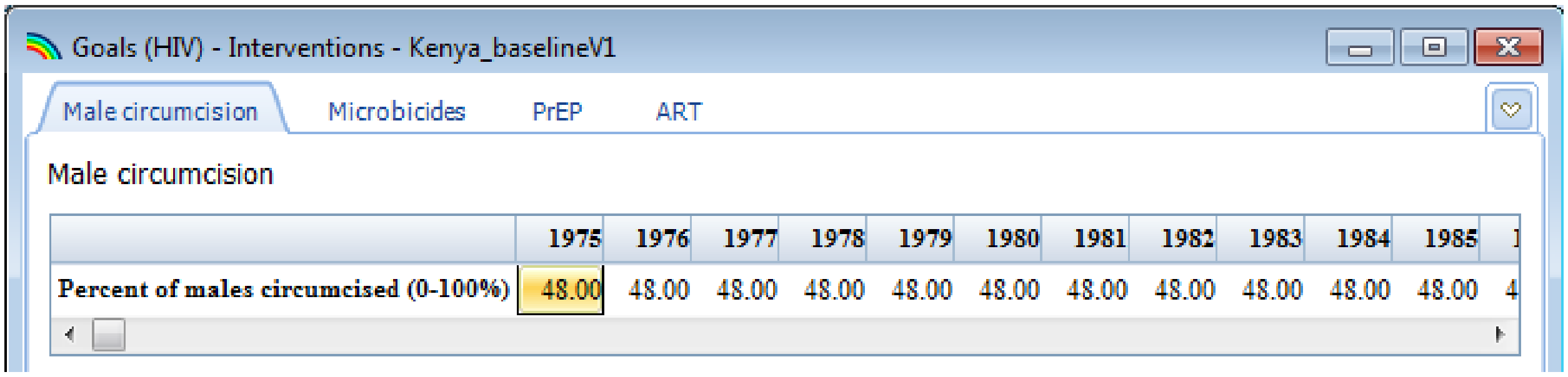

- **Male circumcision:** Enter the proportion of males that are circumcised. This figure is usually constant from the first year of the projection until 2010 and then may change after 2010 if there is a program to expand medical male circumcision.
- **Microbicides.** To test the effect of a new microbicide on HIV transmission enter the expected coverage by risk group. The efficacy of microbicides is specified in the 'Epidemiology' editor.
- **PrEP.** To test the effect of a PrEP (pre-exposure prophylaxis) on HIV transmission enter the expected coverage by risk group. The efficacy of PrEP is specified in the 'Epidemiology' editor.
- **ART.** Here you can enter the number of adults receiving ART by year. Alternatively you can enter the coverage of ART (the percentage of those eligible receiving treatment. Typically you would enter the number on ART for the historical period and either numbers or percentages for the future. Do not enter both numbers and percents in the same year. The entry on migration from first to second line is the proportion of those on first line ARVs moving to second line regimens each year. This is used only for the calculation of the costs of treatment.
- **Eligibility for treatment.** Enter the CD4 count threshold that defines eligibility for treatment by year.

Step 11. Vaccines

If you want to consider the potential impact of future vaccines you can specific the efficacy, target population, mode of action, cost and coverage in the Vaccine editor.

Step 12. Advanced options

The Goals model interacts with both the demographic model (DemProj, which supplies key demographic information) and the HIV/AIDS model (AIM) which used incidence from Goals to calculate other HIV indicators such as the impact of PMTCT (Programs to Prevent Mother-to-Child Transmission) and child indicators. The AIM model uses a similar approach that tracks the HIV-positive population by CD4 count as described in the Methods section of this manual. If you want to change any of the parameters of the CD4 model, such as time in each category or the mortality with or without ART by CD4 category, you can do that by selecting the AIM module and then selecting 'Advanced options' form the AIM menu.

The **Advanced options** menu item gives you access to the default parameter values used in the projection. These parameter values are based on special research and surveys and will not be available for most countries. In most cases the default values should be used. This section allows you to see these default patterns, and change them if you have the information to do so. The patterns are grouped into four categories:

- **Transition parameters**. These include the amount of time a typical HIV+ adult spends in each CD4 category, HIV-related mortality by CD4 category without ART, HIV-related on ART by CD4 count at the initiation of treatment, the annual increase in CD4 counts when on ART, survival on ART for children, the patterns of progression from new infection to death for children, and the effects of HIV infection on fertility.
- **Treatment and PMTCT costs**. This section contains unit costs for key components of treatment and PMTCT. It is only required to produce estimates of the financial resources required.
- **Impacts**. This section contains the assumptions for a very simple calculation of the impact of HIV on the number of TB cases.
- **Orphans**. This section is used to indicate the relative fertility of the HIV+ population in concentrated epidemics in order to provide a better estimate of the number of orphans caused by AIDS.

Step 13. Results

Select the **Results** menu item to see a drop down menu with the categories of HIV/AIDS indicators. They are:

- Model fitting
- Population
- New infections
- Current infections
- AIDS deaths
- Vaccinations
- Benefits
- Cost
- Summary

Each submenu allows you to display results for the entire population or by risk group. Once you choose an indicator the following screen will appear:

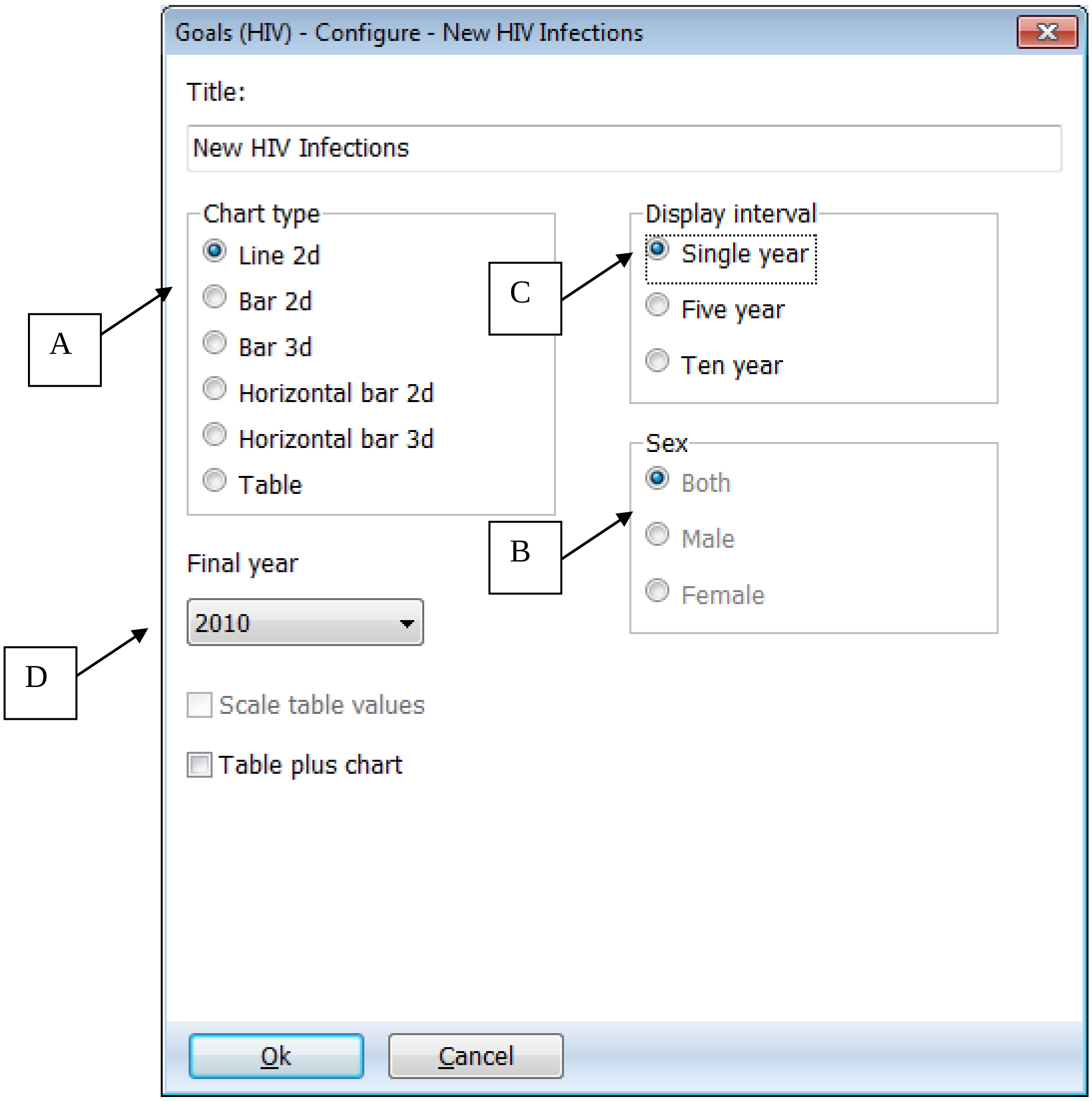

On this screen you can set the options for displaying the results.

A. **Chart type.** Select the type of chart you wish to display.
B. **Sex.** By default this is set to display both sexes, but you can change it to male or female only.
C. **Display interval.** By default this is set to display every year.
D. **Final year.** By default this is set to the final year of your projection or the last selection you made.

Once you have set the options, click **Ok** at the bottom of the screen. This will display a chart showing the indicator you have chosen, according to the variables you have selected on the previous screen. The following is an example of a line graph:

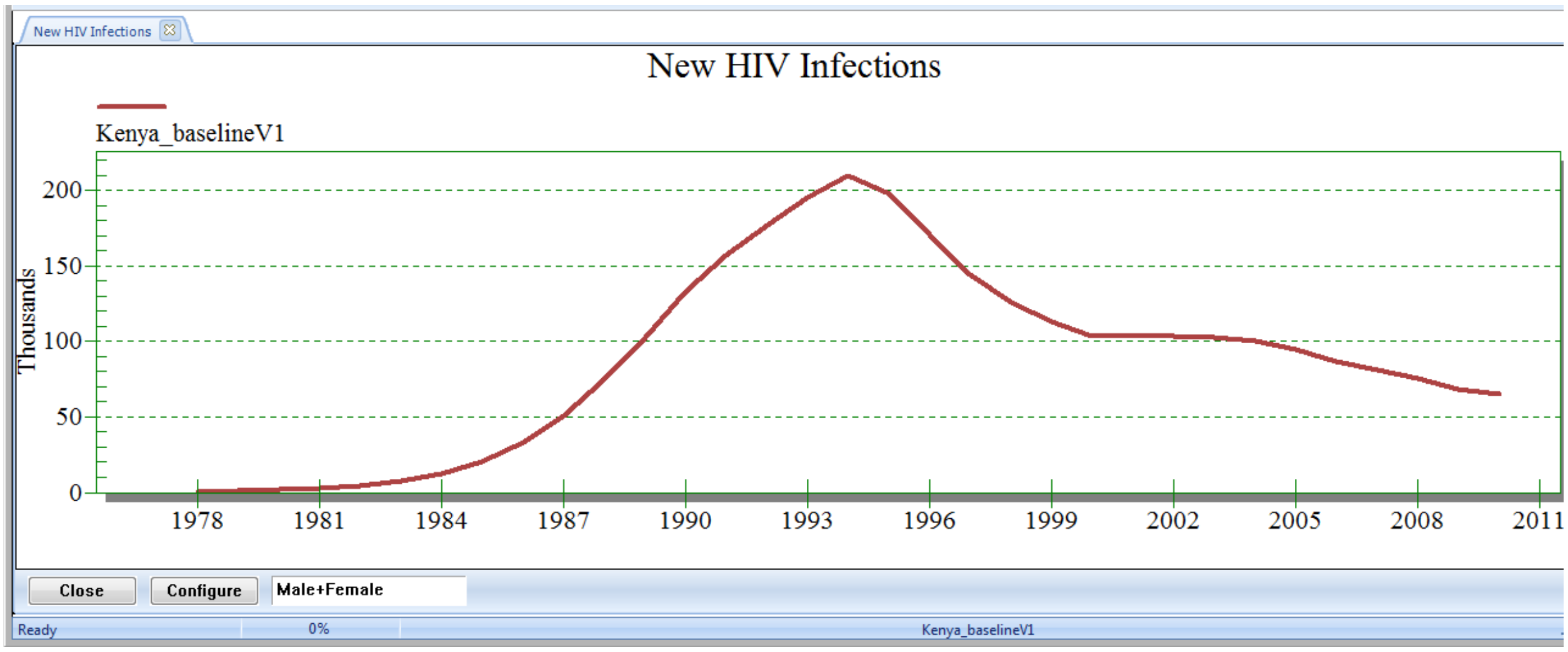

You can open up to 10 projections at one time and display the results in the same chart. The name of each projection that you open will appear at the bottom of the screen.

Step 14. Additional Results

Within Spectrum **Goals** is linked to a demographic projection model (DemProj) and an HIV/AIM module (AIM). You can access additional outputs by clicking on the icons for these modules and selecting 'Results' from their menus.

- **DemProj**. This is the demographic projection component. When you set up a new projection, the demographic inputs will be taken from estimates and projections from the United Nations Population Division. You can change any of these inputs. They include the base year population by age and sex, the total fertility rate, life expectancy without AIDS and international migration. A full set of demographic outputs is available including: total population, population by age and sex, and birth and death rates.
- **AIM**. This module uses an exogenous incidence estimate to project key HIV/AIDS indicators such as the number of new infections, number of AIDS deaths, total number living with HIV, the need for treatment, the need for PMTCT and the number of orphans.

Step 15. Save the projection

Save the projection by click the Spectrum menu button and selecting **Save** or **save As** or by selecting **Home** and clicking the **Save** icon. .

Step 16. Creating alternate projections

You can compare alternate projections by opening two or more files that have exactly the same inputs except for one indicator that you wish to examine. For example you might want to see the effect on new infections of alternate scale-up patterns for certain interventions. The easiest way to do this is to start by opening the base file. Then open the same file again. When you try to do this Spectrum will recognize that you are trying to open the same file twice. It will ask you if you want to go ahead and do this or if you want to rename the projection as you load it. If you choose to rename it, you can provide a new name, such as 'Expanded ART'. Then you will have two projections opens that are exactly the same. You can then edit the 'Expanded ART' projection and change the projected ART coverage. Then you can display the number of AIDS deaths to see the effect of expanded coverage.

When multiple projections are open Spectrum will display the names of the projections at the bottom of the screen and show an asterisk next to the active projection. This is the projection that will appear when you edit the data. To edit a different projection, click the **Set Active** button (when the **Modules** menu item is selected) and select the projection to edit.

Step 17. Uncertainty analysis

Goals has an experimental uncertainty analysis procedure that allows you to determine the uncertainty range for most of the key indicators as a result of uncertainty about the impact of behaviors and biomedical interventions and the epidemiological parameters. This procedure is still under development. It can be used now to examine uncertainty due to future increases in the coverage of behavioral interventions, but procedures for examining uncertainty due to epidemiological parameters are not yet completed.

To start an uncertainty analysis, select **Tools** from the main menu and then click the **Uncertainty Analysis** icon under the Goals section. You will see a display like the one below.

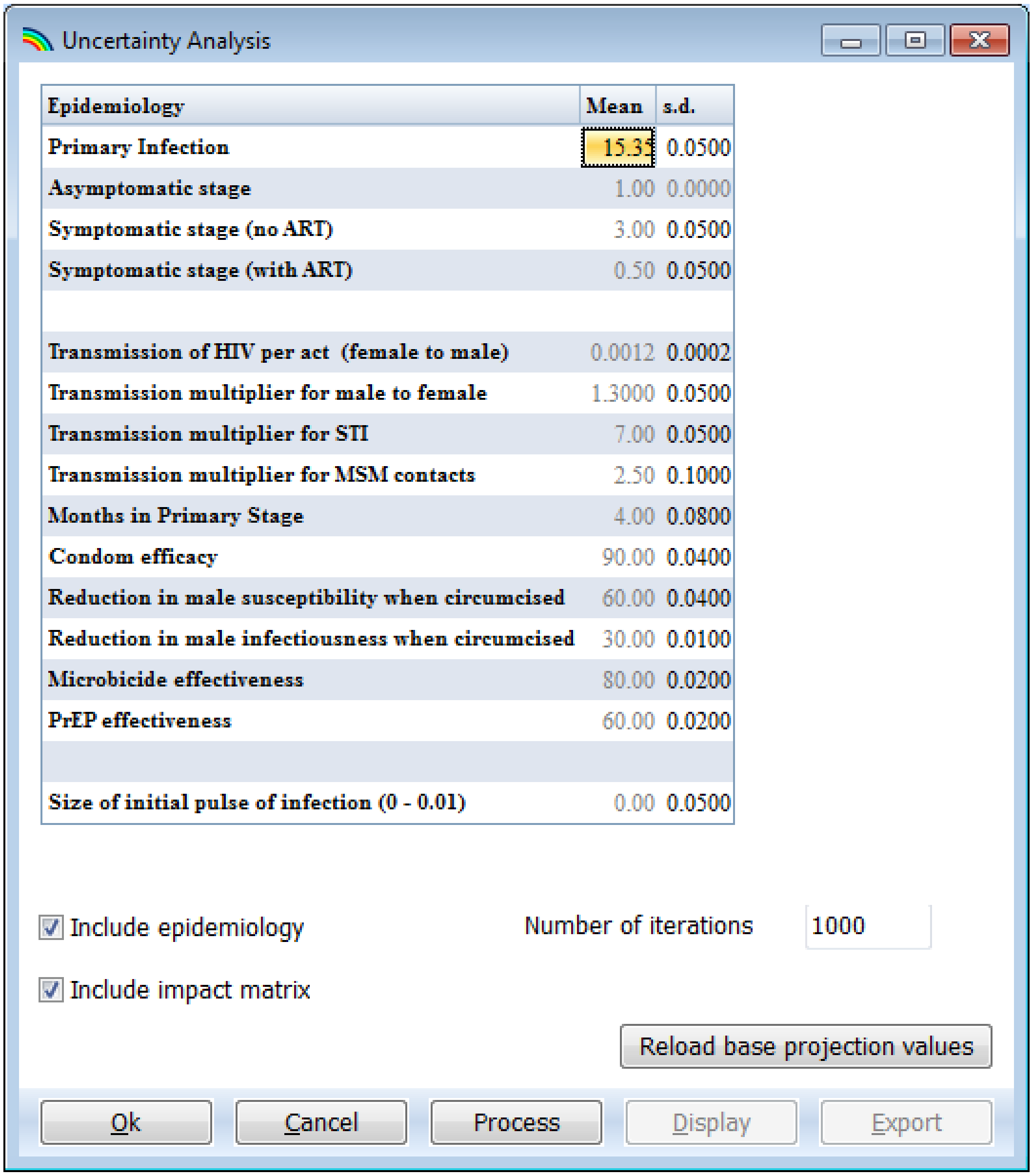

The column labeled '**s.d.**' shows the standard deviation (as a proportion of the mean value) used in the uncertainty analysis. You can change any of these standard values if you wish to try a larger or smaller range. The uncertainty analysis will randomly select parameter values for each of these indicators for each iteration.

To include the uncertainty due just the impact of behavioral interventions select 'Include impact matrix'. You can also include the effects of uncertainty in the epidemiology parameters but note that this will change the historical fit of the model as well. Thus it is more useful for finding appropriate values for model fitting than exploring the future uncertainty in Goals projections.

By default the number of iterations is set to 1000. It will take 40-60 minutes to generate 1000 runs. You can test the procedure by changing this to a smaller number but should generate 1000 curves for your final analysis.

When you are ready to go, click the ‘Process’ button to start the analysis. When it is finished select the ‘Display’ button to select indicators to display the average curve and the 95% plausibility bounds. A summary table, shown below, is available to display the ranges for all indicators for a selected year. The year is selected with the drop down box at the bottom of the display.

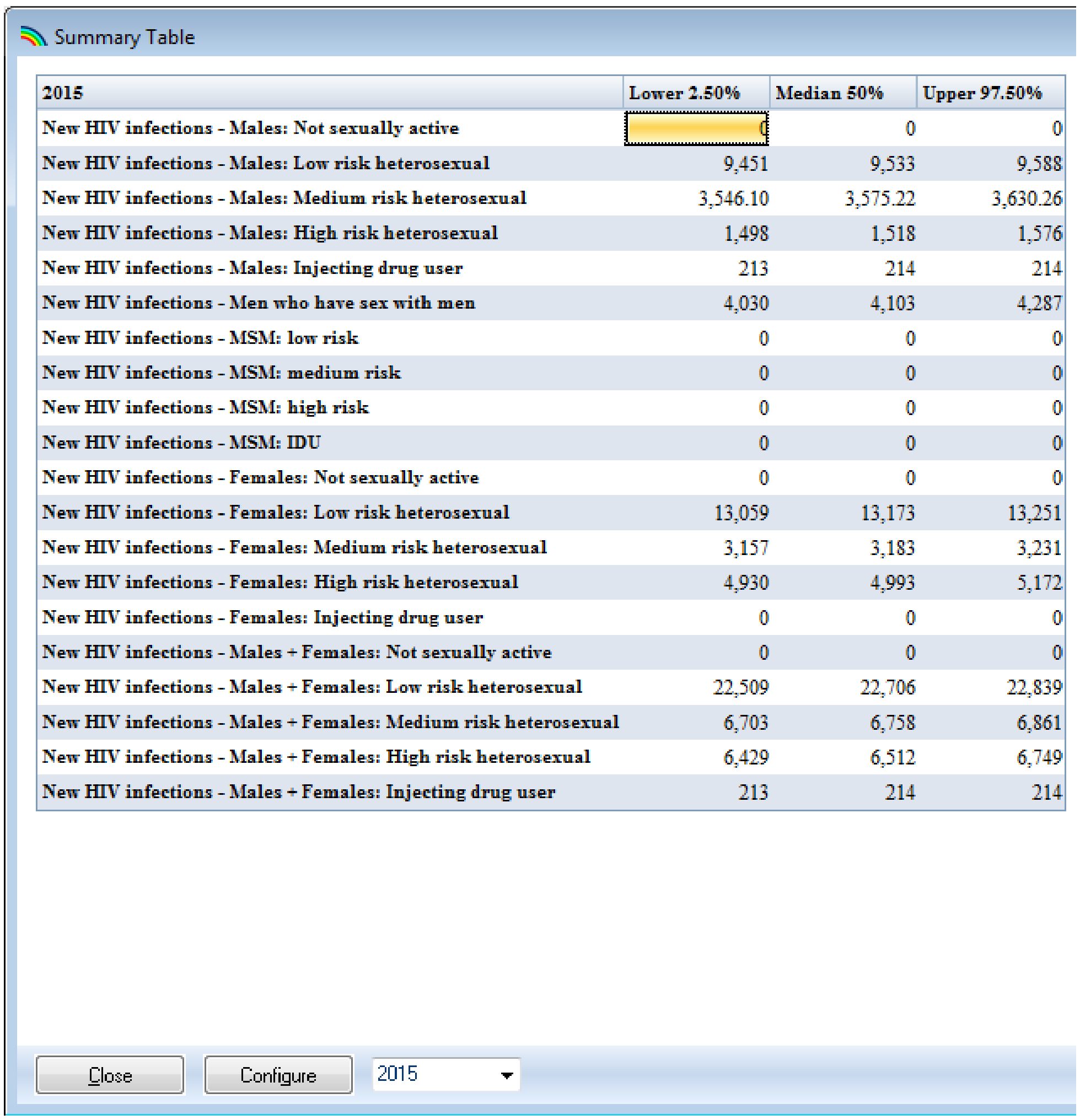

2015	Lower 2.50%	Median 50%	Upper 97.50%
New HIV infections - Males: Not sexually active	0	0	0
New HIV infections - Males: Low risk heterosexual	9,451	9,533	9,588
New HIV infections - Males: Medium risk heterosexual	3,546.10	3,575.22	3,630.26
New HIV infections - Males: High risk heterosexual	1,498	1,518	1,576
New HIV infections - Males: Injecting drug user	213	214	214
New HIV infections - Men who have sex with men	4,030	4,103	4,287
New HIV infections - MSM: low risk	0	0	0
New HIV infections - MSM: medium risk	0	0	0
New HIV infections - MSM: high risk	0	0	0
New HIV infections - MSM: IDU	0	0	0
New HIV infections - Females: Not sexually active	0	0	0
New HIV infections - Females: Low risk heterosexual	13,059	13,173	13,251
New HIV infections - Females: Medium risk heterosexual	3,157	3,183	3,231
New HIV infections - Females: High risk heterosexual	4,930	4,993	5,172
New HIV infections - Females: Injecting drug user	0	0	0
New HIV infections - Males + Females: Not sexually active	0	0	0
New HIV infections - Males + Females: Low risk heterosexual	22,509	22,706	22,839
New HIV infections - Males + Females: Medium risk heterosexual	6,703	6,758	6,861
New HIV infections - Males + Females: High risk heterosexual	6,429	6,512	6,749
New HIV infections - Males + Females: Injecting drug user	213	214	214

III. Fitting the Epidemic

Once all the inputs have been entered in **Goals** you need to fit the model to the epidemic. This involves comparing the model output to the surveillance and survey data and, if necessary, adjusting some of the inputs to better match available data. You can easily compare the model results to available data using the 'Model fitting' selection under the 'Results' menu. When you select this option you will see a display like the following:

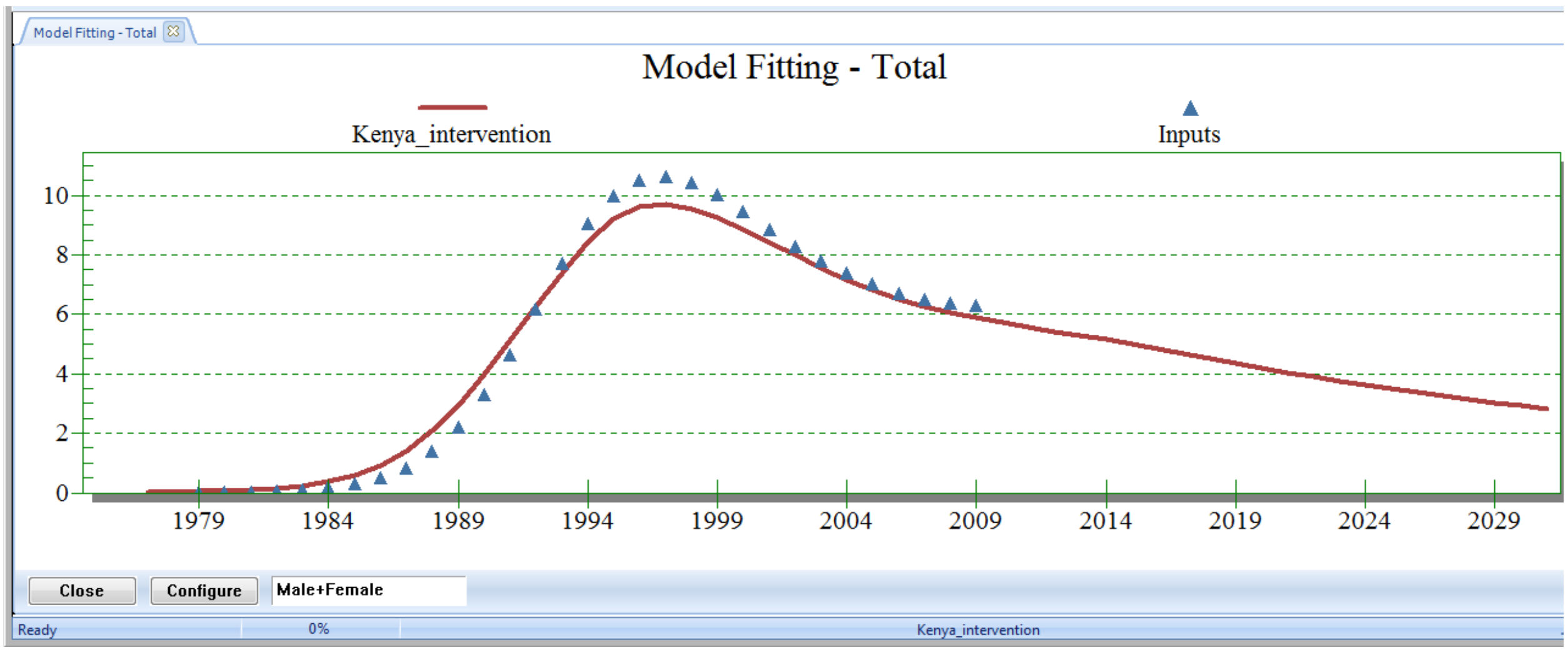

This display compared adult HIV prevalence as estimated by **Goals** in the red line, to the data on prevalence that you entered into the 'HIV prevalence' tab of the 'Epidemiology' editor, shown with the triangles. This particular display shows prevalence among all adults, but you can also display prevalence for any risk group by making an appropriate selection under 'Configure'. Note that you can also examine trends in prevalence for all risk groups at once by selecting 'Results – Current Infections – Prevalence all risk groups'.

If the model does not fit the data well there are a number of changes that you can make to improve the fit.

- **Size of initial pulse of infection**. A larger or smaller value will provide a faster or slower start to the epidemic.
- **Epidemic start year**. Changes will allow the epidemic to start earlier or later.
- **Transmission of HIV per act (female to male)**. A larger value will raise the entire prevalence curve while a smaller value will produce lower prevalence.
- **Primary infection**. A larger value will raise the prevalence curve while a smaller value will reduce it.
- **STI prevalence**. The pattern of decline in STI prevalence will affect the shape of the prevalence curve.

- **IDU force of infection**. To fit the IDU prevalence trend, adjust the male and female force of infection in the Behavior - IDU editor.
- **Condom use**. Changing the trend in condom use will change the shape of the prevalence curve.
- **Number of partners**. Raising the number of partners for medium or high risk groups will increase prevalence among those groups.
- **Acts per partner**. Raising the values will increase prevalence. Coital frequency among low risk population is usually measured at 60 – 110 acts per year. Since recent data on coital frequency are often lacking you may wish to try different values in this range to adjust prevalence among the low risk population.
- **Percent married**. Higher values of percent married will produce higher prevalence in the low risk population and low values will produce lower prevalence in the low risk group.

IV. Description of Methods in the Goals Model in Spectrum

Introduction

The Goals model is intended to support national and international planning for HIV programs by projecting the expected impact and cost of combinations of prevention and treatment programs. It contains a transmission model that calculates the number of new HIV infections over time as a result of sexual and injecting drug transmission. It links to the AIM model to also calculate new child infections due to mother-to-child transmission. Goals can be used to investigate the impact and cost of scaling up different interventions at different rates. The model is intended to support planning efforts by providing information to answer key questions such as:

- How much can we reduce the number of new infections by scaling up a defined package of interventions?
- How much will it cost?
- What goals can be achieved with expected resources?
- How would alternate patterns of resource allocation affect the impact and cost?
- What is the expected impact of new methods of HIV prevention such as pre-exposure prophylaxis (PrEP) or vaccines?

The Goals model is implemented as a module within the Spectrum model. It interacts with other modules in Spectrum. The demographic projection module, DemProj, performs demographic calculations and provides information on the size of the adult population and the number of people becoming adults, aging out of the 15-49 age group and dying from non-AIDS causes. The AIM module uses incidence as calculated by Goals to project the additional indicators such as new child infections due to mother-to-child transmission.

This paper describes the structure and equations of the Goals model.

Structure

The Goals model simulates an HIV epidemic in an adult population aged 15-49. The focus is on this population because most new infections occur in this age group and most survey data on sexual behavior refer to this age group. The adult population is disaggregated by sex and risk group. People enter the model at age 15 and are assumed to not be sexually active until they reach the median age at first sex. Then they are allocated to one of the five risk groups, as shown in Figure 1. The categories were chosen because they are epidemiologically important and information on the proportion of population in the three of the categories (stable couples, multiple partners and sex worker and clients) is available from national surveys.

The risk groups are defined by the behaviors specified in each application, so almost any risk structure might be modeled. However, in practice the standard risk groups are those shown in Figure 1. *Stable couples* are men and women reporting a single partner in the last year. The *multiple partner* category refers to those who report more than one partner in the last year but are not in any of the higher risk categories. *Sex workers and clients* refers to female sex workers and male clients. *MSM* refers to men who have sex with men. *IDU* refers to injecting drug users. While any individual may belong to more than one group, people are classified according to their highest risk.

Figure 1. Risk Structure of Goals

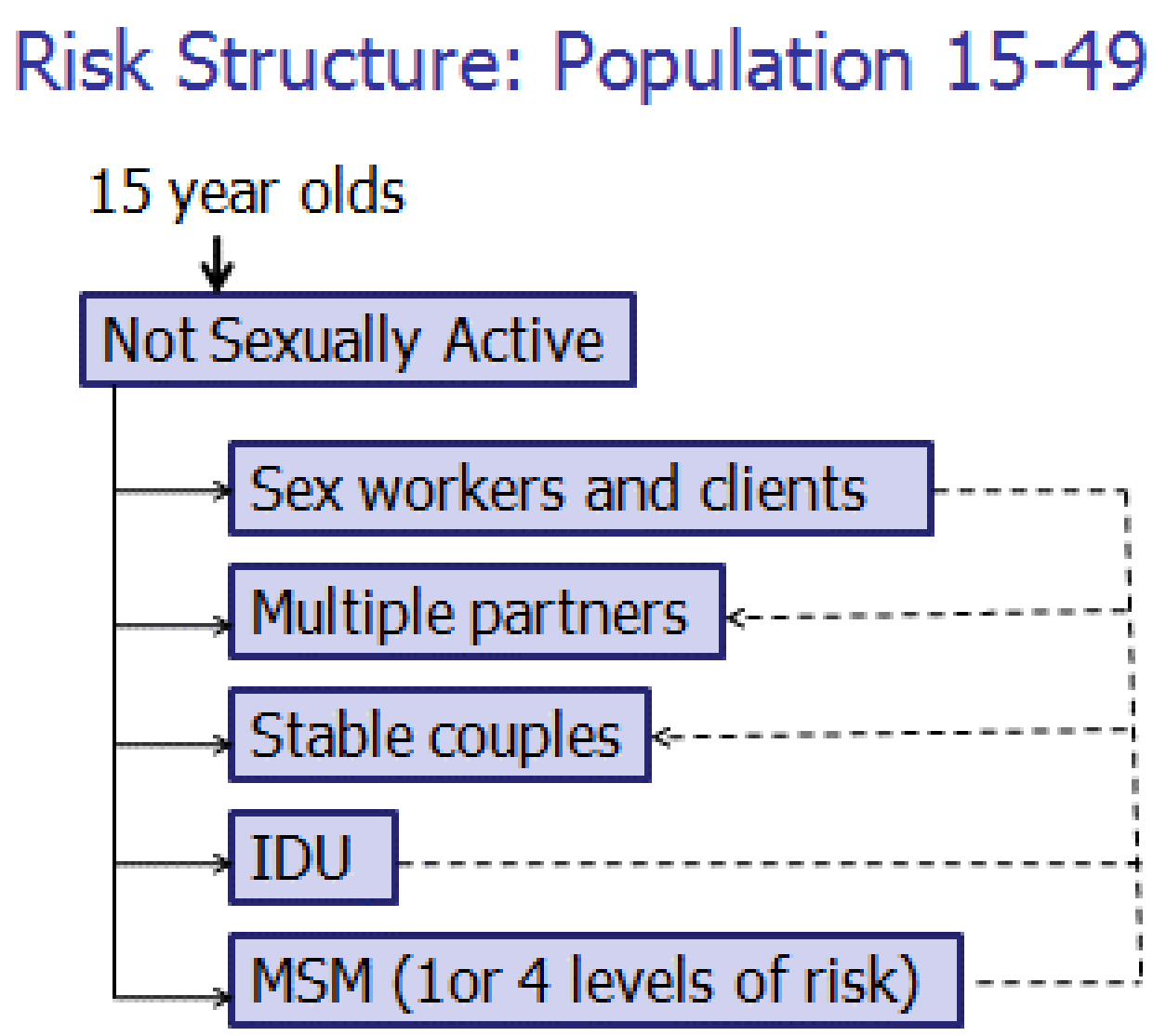

Once a person joins a risk group he or she remains in that risk group until aging out at age 50, dying from non-AIDS cases, dying from AIDS or changing behavior. Behavior change allows people to move from one risk group to a lower risk group. Duration in a risk group may be specified as lifetime or as an average number of years. For example, duration in sex work might be set to 5 or 10 years. Those leaving the highest risk groups (sex work, MSM, IDU) move to the medium risk group (casual sex) and those leaving the casual sex group move to the stable couples risk group.

Sexual Transmission of HIV

Transmission of HIV from an infected partner to an uninfected partner depends on the characteristics of both partners and the partnership, as shown in Figure 2. For the susceptible partner the important characteristics are the number of partners, if male whether he is circumcised, and whether or not new prevention methods, such as PrEP or vaccines, are used. Transmission from the infected partner is affected by the stage of infection (primary, asymptomatic, or symptomatic), whether the partner is receiving ART and whether the partner has been vaccinated with an HIV vaccine that reduces infectiousness. Characteristics of the partnership that influence transmission are the number of acts per partner per year, whether either partner has a sexually transmitted infection, the type of contact (heterosexual, MSM or needle sharing) and condom use.

Figure 2. Characteristics determining transmission of HIV

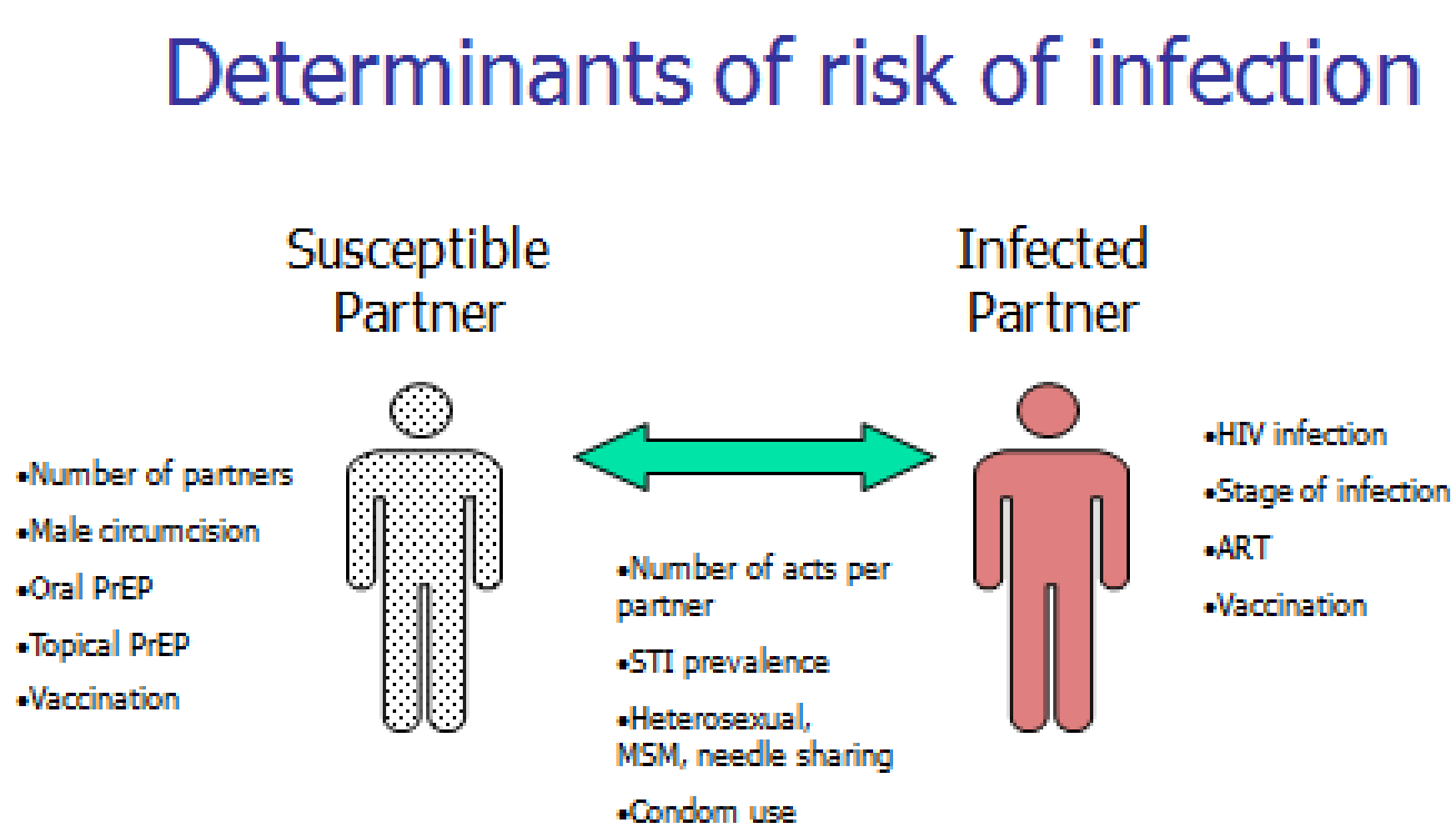

New infections, *I*, are calculated as the susceptible population, X, multiplied by the probability of becoming infected, ι.

$$I_{s,k,t} = X_{s,k,t} \times \iota_{s,k,t}$$

Where

s	= Sex, male or female
k	= Risk group
t	= Time

The probability of transmission to an uninfected partner during one year, ι, is given by the equation below.

$$1-\left[P_{s',k,t}\times\left(1-r\times R_t\times MC_{k,t}\times C_{k,t}\times V_{k,t}\times S_{k,t}\times \mathrm{Pr}_{k,t}\right)^a+\left(1-P_{s',k,t}\right)\right]^n$$

Where

$P_{s',k,t}$	= HIV prevalence in the partner population of risk group k at time *t*
r	= Base probability of HIV transmission per act
R_t	= Multiplier for the effect of stage of infection
$C_{k,t}$	= Multiplier for effect of condom use
$MC_{k,t}$	= Multiplier for effect of male circumcision
$S_{k,t}$	= Multiplier for effect of sexually transmitted infections
$Pr_{k,t}$	= Multiplier for effect of PrEP
$V_{k,t}$	= Multiplier for effect of HIV vaccines
a	= Number of acts per partner per year
n	= Number of partners per year

Prevalence in the partner population is defined differently for each risk group. For sex workers or clients and those with multiple partners, it is the prevalence of the population of the opposite sex in the same risk group. For MSM it is the prevalence in the MSM group. For stable couples the prevalence in the partner population is a weighted average of the prevalence in each risk group *P* multiplied by the proportion of contacts with that risk group *A*.

$$P_{s,k,t}=\sum_k P_{s',k,t}\times A_{s,k,t}$$

For those in stable relationships the proportion of contacts with each risk group is calculated from the number of people in each risk group N_k and the proportion married *m*.

$$A_{s,k,t}=\sum_k m_{s,k}\times N_{s,k,t}\div\sum_k N_{s,k,t}$$

The effect of stage of infection is calculated as a weighted average of infectiousness by stage *i* and the proportion of the population in each stage.

$$R_t=\sum_i\sum_k\sum_s Y_{i,k,s,t}\times IM_i\div\sum_i\sum_k\sum_s Y_{i,k,s,t}$$

Where

$Y_{i,k,s,t}$	= HIV+ population in stage *i* risk group k sex *s* at time *t*.
IM_i	= Infectiousness multiplier of stage *i*

The stages are primary infection, asymptomatic, symptomatic (CD4 count < 200 cells/μl) and on ART.

The impact of sexually transmitted infections (STIs) is calculated from the prevalence of STIs in either partner and the increase in transmission when STIs are present.

$$S_{k,t} = 1 + Sm \times Sp_{k,t}$$

Where

Sm = Multiplier on the probability of transmission when either partner has an STI
$Sp_{k,t}$ = Proportion of risk group k with an STI at time *t*

Transmission among Injecting Drug Users

The probability of transmission through drug injections is determined by a force of infection variable that expresses the rate of contact between sharing groups, *f*, the prevalence of HIV among IDU *P*, the multiplier for stage of infection *R* and any effects of new technologies such as vaccines *V* or PrEP *Pr*.

$$f_t \times P_{k,t} \times R_t \times \mathrm{Pr}_{k,t} \times V_{k,t}$$

Impact of Biomedical Interventions

The impacts of biomedical interventions are included in the transmission equation by the multipliers for condoms, *C*, male circumcision, *MC*, PrEP, *Pr*, and vaccines, *V*. The calculation of each multiplier follows the same form, (1 – effectiveness) multiplied by the percentage of the population covered.

$$C_{k,t} = 1 - Ce \times Cc_{k,t}$$

Where

Ce = Condom effectiveness
$Cc_{k,t}$ = Proportion of risk group k using condoms at time *t*

Similarly,

$$MC_{k,t} = 1 - MCe \times MCc_{k,t}$$
$$\Pr_{k,t} = 1 - \Pr e \times \Pr c_{k,t}$$
$$V_{k,t} = 1 - Ve \times Vc_{k,t}$$

Impact of Behavior Change Interventions

The impact of behavior change is expressed in the transmission equation by the variables for condom use, *Cc,* and the number of partners, *n*. Behavior change interventions can also affect age at first sex, which determines when people become sexually active, and needle sharing among IDUs, which determined the proportion of injecting drug users that is susceptible to infection.

The impact of interventions on each of these behaviors is determined by an impact matrix that describes the impact of each intervention on each behavior for each risk group. The impact matrix is based on an extensive literature search which is described in detail elsewhere[1].

The impact matrix describes behavior changes for each behavior and each risk group as shown in Tables 1-4 below.

For condoms, Table 1, impact is calculated as a reduction in the non-use of condoms in order to allow for the aggregation of impacts when several interventions are present. Thus, condom use is calculated as one minus the non-use of condoms in the base year multiplied by the product across all interventions of the increase in coverage of each intervention and it impact on condom non-use:

$Cc_t = 1 - (1 - Cc_o) \times \prod_i (\text{Coverage}_{i,t} - \text{Coverage}_{i,0}) \times \text{CondomImpact}_i$

Table 1. Reduction in Non-Use of Condoms when Exposed to Behavioral Interventions

**Intervention**	**Reduction in non-use of condoms**				
	**High**	**Medium**	**Low**	**MSM**	**IDU**
**Community mobilization**		-10	-2.5		
**Mass media**		-11.6	-17		
**VCT**	-44.2	-23.4	-16.1		
**Condoms**	-85.1	-55.1	-5.3		
**Youth: in-school**		-15.7			
**Youth: out-of-school**		-16.6			
**Workplace programs**		-17.1	-1.0		
**Outreach**	-42.5	0		-36.3	-17.9

**MSM: lubricants**	0	0
**IDU: needle sharing**	-41.5	0
**IDU: drug substitution**	0	0

The impact on the number of sexual partners (Table 2) is determined as the number of partner in the base year multiplied by the product of the increase in coverage and the impact of each intervention.

$n_t = n_0 \times \prod_i (\text{Coverage}_{i,t} - \text{Coverage}_{i,0}) \times \text{Number of Partners Impact}_i$

Table 2. Reduction in Number of Partners per Year when Exposed to Behavioral Interventions

	**Reduction in # of partners**				
	**High**	**Medium**	**Low**	**MSM**	**IDU**
**Community mobilization**					
**Mass media**		-4.2			
**VCT**		-13.1			
**Condoms**		-13			
**Youth: in-school**		-18.3			
**Youth: out-of-school**		-18.3			
**Workplace programs**		-35.5			
**Outreach**	-11.2			-10.1	-1.2
**MSM: lubricants**					
**IDU: needle sharing**					
**IDU: drug substitution**	-46				

The impact on age at first sex, Table 3, is calculated as age at first sex in the base year multiplied by the product of the increase in coverage and the impact of each intervention.

$AFS_t = AFS_o \times \prod_i (\text{Coverage}_{i,t} - \text{Coverage}_{i,0}) \times \text{Age at First Sex Impact}_i$

Table 3. Increase in Age at First Sex When Exposed to Behavioral Interventions

**Community mobilization**	-0.3
**Mass media**	
**VCT**	
**Condoms**	
**Youth: in-school**	0.1
**Youth: out-of-school**	0.1
**Workplace programs**	
**Outreach**	
**MSM: lubricants**	
**IDU: needle sharing**	

IDU: drug substitution

Similarly the impact on needle sharing, Table 4, is calculated as the proportion sharing needles in the base year multiplied by the product of the increase in coverage and the impact of each intervention.

NS_t = NS_o x $\prod_i$ ($Coverage_{i,t}$ – $Coverage_{i,0}$) x Needle Sharing $Impact_i$

Table 4. Reductions in Unsafe Injecting Behaviors When Exposed to Behavioral Interventions

	Needle Sharing
**Outreach**	-17.9
**IDU: needle sharing**	-50.7
**IDU: drug substitution**	-65.0

Progression of HIV-Infected Population

The HIV+ population is tracked by CD4 count. The model structure has seven CD4 compartments as shown below. The compartments were selected on the basis of eligibility criteria and mortality patterns.

We assume that most newly infected people start with CD4 counts above 500, although some portion, p, can start at 350-499. The transition probabilities λ1, λ2, λ3, λ4, λ5 and λ6 represent the probability of progressing from one CD4 category to the next. In each category there is some probability of death from HIV-related causes, designated as μ1, μ2, μ3, μ4, μ5, μ6 and μ7 as well as a chance of death from non-AIDS causes, μ0 (not shown in the figure). The probability of HIV-related death increases as CD4 counts decrease.

The number of people in the different CD4 count categories represents the HIV-infected population not on ART. The number of people eligible for treatment is the number in each CD4 count category that is below the recommended level for initiating ART.

Depending on the eligibility criterion and the level of first line ART coverage, a percentage of those eligible for treatment will start first line ART (c1, c2, c3, c4, c5, c6, c7). Those on ART are categorized by their CD4 count at the initiation of treatment. The model does not track the temporal decline of CD4 counts of those on treatment. Those on first line ART have a probability of failure depending on their CD4 count at initiation, α1, α2, α3, α4, α5, α6 and α7.

The number starting ART each year is determined by the assumed coverage and the number of people in the eligible for treatment. We assume that those starting on ART will be distributed

among the eligible CD4 categories such that an equal percentage of people in each eligible CD4 category initiate treatment.

Figure 1. Model of HIV-Infected Population, Eligibility for ART and HIV-related Mortality

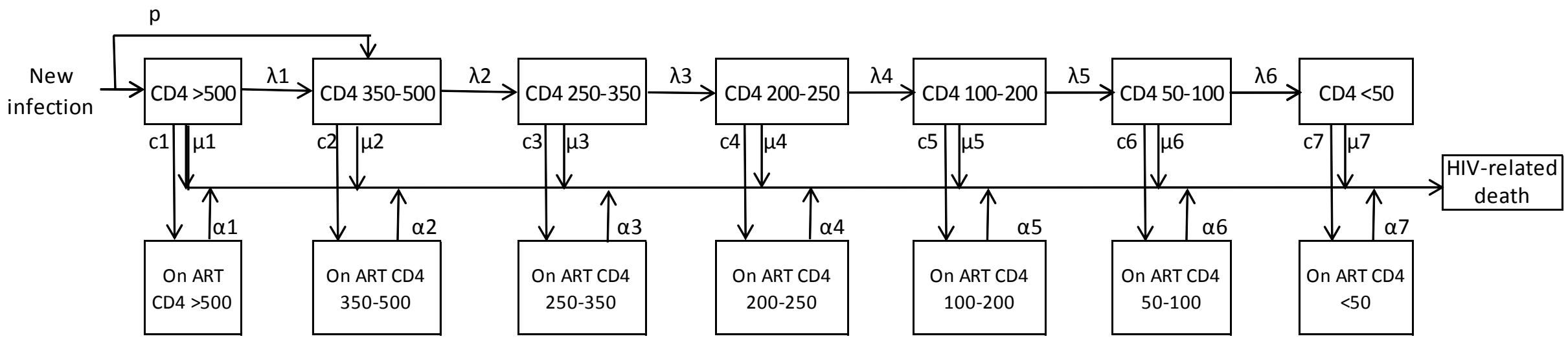

Notes:
λ1, λ2, λ3, λ4, λ5, λ6 are the annual probabilities of transitioning to the next lower CD4 group
μ1, μ2, μ3, μ4, μ5, μ6, μ7 are the annual probabilities of HIV-related mortality by CD4 group
α1, α2, α3, α4, α5, α6, α7 are the annual mortalities on ART by CD4 count

Allocation of New ART Patients by CD4 Count

The number of people on ART in each year is an input to Spectrum. It is used to determine the number of people newly starting ART in each year in order to achieve the specified number of patients. New ART patients need to be allocated across CD4 categories. Information from treatment cohorts on the distribution of new patients by CD4 count is based on a time when almost all countries defined eligibility for treatment as those with CD4 counts under 200. As most countries are now moving to the new WHO guidelines as <350, past patterns may not indicate future patterns.

We have considered two options for allocating new ART patients by CD4 count:

1. Start the same proportion on ART from each eligible CD4 category
2. Allocate new ART patients on the basis of expected AIDS mortality without ART.

When comparing the two methods with data from southern Africa treatment sites it appears that the first method (allocating an equal proportion from all eligible CD4 categories) tends to under-estimate the number starting at low CD4 counts and over-estimate those starting at higher CD4 counts. Weighting the allocations by expected AIDS mortality produced the opposite results, there are too many starting at low CD4 counts and too few at higher CD4 counts. An average of the two methods produces a pattern that matches the data reasonably well. Therefore, Spectrum calculates allocations according to both methods and averages the results.

Demographic Processes

People enter the model when they reach age 15. The number of people reaching age 15 in each year is provided to Goals from the demographic projection component of Spectrum. Similarly,

people age out of the adult population in Goals when they reach the age of 50. Those aging out at 50 are assumed to have the same characteristics as the rest of the adult population in terms of risk group and HIV status.

In addition to aging, adults in Goals are also subject to non-AIDS mortality which is supplied to Goals by the demographic projection model in Spectrum based on age-specific non-AIDS mortality rates.

Parameter Values

Values of key parameters and the sources are shown in Table 5.

Parameter	Value	Source
Transmission of HIV per act (female to male)	0.0011	Baggeley *et al*[2]., Gray *et al.*
Multiplier on transmission per act for		
- Male to female	1.0	
- Presence of STI	8	Galvin and Cohen[3], 2.2-11.3<br>Powers *et a.l*[4]. 5.1-8.2
- MSM contacts	2.6	Vittinghoff *et al*[5].
Relative infectiousness by stage of infection		
- Primary infection	9 – 40	Boily *et a.l*[6]. 9.17 (4.47-18.81)<br>Pinkerton[7]
- Asymptomatic	1	Reference stage
- Symptomatic	7	Boily *et al*[6]. 7.27 (4.45-11.88)
- On ART	0.04 – 0.08	Cohen *et al.*[8]<br>Attia *et al.*[9]
Efficacy in reducing HIV transmission		
- Condom use	0.8	Weller and Davis[10]
- Male circumcision	0.6	Auvert *et al*[11], Gray *et al*. (2007)[12], Bailey *et al.*[13]
- PrEP	0.55 – 0.73	Grant *et al.*[14]<br>Partners PrEP Study
- Microbicide	0.6	Abdool Karim *et al.*[15]

The time in each CD4 count category is shown in Table 7. These values have been determined by starting with values in the published literature and then fitting the model to data from the ALPHA network. Progression from infection to death is slower for young people than older people. The ALPHA network analysis[1] found a median time from infection to AIDS death of 12.8 years for those aged 15-24, 10.6 years for 25-34, 7.5 years for 35-44, and 56 years for 45+. They found no difference by sex once survival was adjusted for age. We have used these data to develop parameter sets for these four age groups.

A recent publication by Johansson *et al.* summarizes data from 6 countries on mortality by CD4 count for those not on ART[16]. They found annual mortality of 0.8 (0.7-0.9) for those with CD4 counts <50 (from one study in Thailand), 0.355 (0.335-0.375) for those with CD4 counts

between 50 and 199 from 6 studies, and 0.109 (0.093-0.125) for those with CD4 counts between 250 and 350 from six studies.

Information on the rate of CD4 cell count decline is available from a several studies, with one providing declines by CD4 category (Table 6).

Table 6. Annual declines in CD4 count among HIV-positive adults

**Study**	**Annual Rate of Decline in CD4 Count**
CASCADE, Wolbers et al.[17]	61 (46-81) Fixed effects<br>74 (31-145) By patient<br>114 (32-229) Last 2 tests
MACS, Mellors et al. JAMA, 2007[18]	64 (8-136)
CNICS, SFMHS, REACH, Rodriguez et al.[19]	50 (46-55)
Williams et al. JID, 2006[20]	85 South Africa<br>65 Zambia
Cape Town, Holmes et al.[21]	>500: 47 (40-54)<br>351-500: 31 (23-38)<br>201-350: 20 (14-27)

For this model the parameter values were determined by using the literature values as a starting point and fitting the model to Weibull curves of progression to mortality by age from the ALPHA network. We assumed that the rate of CD4 decline is constant across all categories and that mortality varies by CD4 category but not by age. The resulting parameter values are shown in Table 7.

Table 7. Number of years in each CD4 count category by age and sex

	Male				Female			
	15 - 24	25 - 34	35 - 44	45 -54	15 - 24	25 - 34	35 - 44	45 -54
> 500	8.02	6.95	3.96	2.47	8.02	6.95	3.96	2.47
350 - 499	3.35	2.43	1.53	0.94	3.35	2.43	1.53	0.94
250 - 349	2.23	1.62	1.02	0.63	2.23	1.62	1.02	0.63
200 - 249	1.12	0.81	0.51	0.31	1.12	0.81	0.51	0.31
100 - 199	2.23	1.62	1.02	0.63	2.23	1.62	1.02	0.63
50 - 99	1.12	0.81	0.51	0.31	1.12	0.81	0.51	0.31

Mortality while on ART has been estimated by the IeDEA (International Epidemiologic Database to Evaluate AIDS) Consortium which has analyzed data from 50,000 patients in East

Africa to determine mortality of patients on ART by CD4 count at treatment initiation. In the future, mortality patterns will become available for other regions: southern Africa, West Africa, Latin America and Asia. The actual patterns used in Spectrum vary by age, sex and time on ART: 0-6 months, 7-12 months and more than 12 months as shown in Table 8.

Table 8. Annual mortality (per 100 person-years) on ART by CD4 count, age, sex and time on ART

**CD4 Count by Time on ART**	**Males**				**Females**			
**0-6 Months**	15-24	25-34	35-44	45+	15-24	25-34	35-44	45+
>500	5.84	4.42	3.91	4.40	4.22	3.19	2.82	3.18
350-500	10.53	7.97	7.05	7.93	7.61	5.76	5.09	5.73
250-349	14.33	10.85	9.59	10.79	10.35	7.83	6.93	7.80
200-249	11.13	8.43	7.45	8.38	8.04	6.09	5.38	6.06
100-199	12.78	9.68	8.55	9.63	9.23	6.99	6.18	6.95
50-99	22.74	17.21	15.22	17.13	16.42	12.43	10.99	12.37
<50	41.69	31.56	27.90	31.40	30.11	22.79	20.15	22.68
**7-12 months**								
>500	1.76	1.17	1.19	1.42	1.17	0.78	0.80	0.95
350-500	2.36	1.57	1.60	1.91	1.58	1.05	1.07	1.28
250-349	2.16	1.44	1.47	1.75	1.44	0.96	0.98	1.17
200-249	1.72	1.14	1.16	1.39	1.15	0.76	0.78	0.93
100-199	2.11	1.40	1.43	1.71	1.41	0.94	0.96	1.14
50-99	2.70	1.80	1.83	2.19	1.81	1.20	1.23	1.46
<50	3.43	2.28	2.33	2.78	2.29	1.52	1.56	1.86
**>12 months**								
>500	1.08	0.72	0.74	0.88	0.73	0.48	0.49	0.59
350-500	1.45	0.97	0.99	1.18	0.97	0.65	0.66	0.79
250-349	1.33	0.89	0.90	1.08	0.89	0.59	0.60	0.72
200-249	1.06	0.70	0.72	0.86	0.71	0.47	0.48	0.57
100-199	1.30	0.87	0.88	1.05	0.87	0.58	0.59	0.71
50-99	1.67	1.11	1.13	1.35	1.11	0.74	0.76	0.90
<50	2.12	1.41	1.44	1.71	1.42	0.94	0.96	1.15

References

[1] Bollinger LA, How can we calculate the "E" in "CEA" *AIDS* 2008, 22(suppl 1): S51-S57.
[2] Baggaley RF, Fraser C. Modelling sexual transmission of HIV: testing the assumptions, validating the predictions. Curr Opin HIV AIDS. 2010; **5**(4): 269-76.
[3] Galvin and Cohen, "The Role of Sexually Transmitted Diseases in HIV Transmission" Nature Reviews Microbiology Volume 3, January 2004, pps. 33-42.
[4] Powers KA, Poole C, Pettifor AE, Cohen MS Rethinking the heterosexual infectivity of HIV-1: a systematic review and meta-analysis The Lancet Published on line August 5, 2008 DOI:10.1016/S1273-3099(08)70156-7.
[5] Vittinghoff E, Douglas J, Judson F, McKirnan D, MacQueen K, Buchbinder SP. Per-Contact Risk of Human Immunodeficiency Virus Transmission between Male Sexual Partners Am J Epidemiol (1999)150:3;306-31 suggests 0.0016/0.0011.
[6] Boily MC, Baggaley RF, Wang L, Masse B, White RG, Hayes RJ, Alary M. Heterosexual risk of HIV-1 infection per sexual act: systematic review and meta-analysis of observational studies *Lancet Infect Dis* 2009; 9: 118-29.
[7] Pinkerton SD. Probability of HIV transmission during acute infection in Rakai, Uganda. AIDS Behav. 2008; **12**(5): 677-84.
[8] Cohen MS, Chen YQ, McCauley M, Gamble T, Hosseinipour MC, Kumarasamy N, *et al.* Prevention of HIV-1 Infection with Early Antriretroviral Therapy N Engl J Med 2011; 10.1056/NEJMoa1105243.
[9] Attia S, Egger M, Muller M, Zwahlen M, Low N. Sexual transmission of HIV according to viral load and antiretroviral therapy: systematic review and meta-analysis. AIDS 2009, 23:1-8.
[10] Weller S, Davis, K. Condom effectiveness in reducing heterosexual HIV transmission (Cochrane Review). In: The Cochrane Library, Issue 1, 2004. Chichester, UK: John Wiley & Sons, Ltd.
[11] Auvert B, Puren A, Taljaard D, Lagarde E, JoëlleTambekou-Sobngwi, RémiSitta. The impact of male circumcision on the female-to-male transmission of HIV : Results of the intervention trial : ANRS 1265. IAS 2005: INSERM, France; 2005.
[12] Bailey RC, Moses S, Parker CB, Agot K, Maclean I, Krieger JN, et al. Male circumcision for HIV prevention in young men in Kisumu, Kenya: a randomised controlled trial. Lancet. 2007; **369**(9562): 643-56.
[13] Bailey RC, Moses S, Parker CB, Agot K, Maclean I, Krieger JN, et al. Male circumcision for HIV prevention in young men in Kisumu, Kenya: a randomised controlled trial. Lancet. 2007; 369(9562): 643-56.
[14] Grant RM, Lama JR, Anderson PL, McMahan V, Liu AY, Vargas L. Preexposure Chemoprophylaxis for HIV Prevention in Men Who Have Sex with Men New Engl J Med 2010, 10.1056/NEJMoa1011205.
[15] Karim QA, Karim SSA, Frohlich J, Grobler AC, Baxter C, Mansoor LE, *et al.* Effectiveness and Safety of Tenofovir Gel, an Antoretroviral Microbicide, for the Prevention of HIV Infection in Women. *Science* 329; 1168-1174 (September 2010).
16 Johansson KA, Robberstad B, Norheim OF. Further benefits by early start of HIV treatment in low income countries: survival estimates of early versus deferred antiretroviral therapy. AIDS Research and Therapy 2010, 7:3.
[17] Wolbers M, Babiker A, Sabin C, Young J, Dorrucci M, Chene G, et al. Pretreatment CD4 cell slope and progression to AIDS or death in HIV-infected patients initiating antiretroviral therapy--the CASCADE collaboration: a collaboration of 23 cohort studies. PLoS Med. 2010; 7(2): e1000239.
[18] Mellors JW, Margolick JB, Phair JP, Rinaldo CR, Detels R, Jacobson LP, et al. Prognostic value of HIV-1 RNA, CD4 cell count, and CD4 Cell count slope for progression to AIDS and death in untreated HIV-1 infection. JAMA. 2007; 297(21): 2349-50.
[19] Rodriguez B, Sethi AK, Cheruvu VK, Mackay W, Bosch RJ, Kitahata M, et al. Predictive value of plasma HIV RNA level on rate of CD4 T-cell decline in untreated HIV infection. JAMA. 2006; 296(12): 1498-506.
[20] Williams BG, Korenromp EL, Gouws E, Schmid GP, Auvert B, Dye C. HIV infection, antiretroviral therapy, and CD4+ cell count distributions in African populations. J Infect Dis. 2006; 194(10): 1450-8.
[21] Holmes CB, Wood R, Badri M, Zilber S, Wang B, Maartens G, et al. CD4 Decline and Incidence of Opportunistic Infections in Cape Town, South Africa: Implications for Prophylaxis and Treatment. JAIDS Journal of Acquired Immune Deficiency Syndromes. 2006; 42(4): 464-9 10.1097/01.qai.0000225729.79610.b7.
